# Supplementary material for: Accelerating scientific discovery with Co-Scientist
Source: Nature. 2026 May 19;655(8122):487–96. doi: 10.1038/s41586-026-10644-y (PMC13345910; doi:10.1038/s41586-026-10644-y)
Supplement: Supplementary file 1 — Supplementary Notes 1–12, Supplementary Figs. 1–9, Supplementary Tables 1–4 and Supplementary References. [file 41586_2026_10644_MOESM1_ESM.pdf]

nature portfolio

https://doi.org/10.1038/s41586-026-10644-y

Supplementary information

Accelerating scientific discovery with Co-Scientist

In the format provided by the authors and unedited

Supplementary Information

Accelerating scientific discovery with Co-Scientist

Juraj Gottweis[1*‡], Wei-Hung Weng[2*‡], Alexander Daryin[1*], Tao Tu[2*], Petar Sirkovic[1*], Artiom Myaskovsky[1*], Grzegorz Glowaty[1*], Felix Weissenberger[1*], Alessio Orlandi[1*], Dan Popovici[3], Anil Palepu[3], Keran Rong[2], Ryutaro Tanno[2], Khaled Saab[2], Fan Zhang[3], Jacob Blum[4], Andrew Carroll[3], Kavita Kulkarni[3], Nenad Tomašev[2], Dina Zverinski[1], Ivor Rendulic[1], Elahe Vedadi[2], Florian Hasler[1], Luka Rimanic[1], Marina Boia[1], Ivan Budiselic[1], Ben Feinstein[3], Mathias Bellaiche[3], Tom Sheffer[3], Jan Freyberg[2], Jeremy Ratcliff[2], Ottavia Bertolli[2], Katherine Chou[3], Avinatan Hassidim[3], Burak Gokturk[1], Amin Vahdat[1], Yuan Guan[4], Vikram Dhillon[5], Eeshit Dhaval Vaishnav[6], Byron Lee[6], Tiago R D Costa[7], José R Penadés[7], Gary Peltz[4], Yossi Matias[3], James Manyika[3], Demis Hassabis[2], Yunhan Xu[2], Pushmeet Kohli[2‡], Annalisa Pawlosky[3‡], Alan Karthikesalingam[2‡], Vivek Natarajan[2‡]

[1] Google Cloud AI Research, Zurich, Switzerland
[2] Google DeepMind, Mountain View, California, USA
[3] Google Research, Mountain View, California, USA
[4] Stanford University School of Medicine, Palo Alto, California, USA
[5] Houston Methodist, Houston, Texas, USA
[6] Sequome, South San Francisco, California, USA
[7] Fleming Initiative and Imperial College London, London, UK

[*]These authors contributed equally: Juraj Gottweis, Wei-Hung Weng, Alexander Daryin, Tao Tu, Petar Sirkovic, Artiom Myaskovsky, Grzegorz Glowaty, Felix Weissenberger, Alessio Orlandi.
[‡]Corresponding to Juraj Gottweis (juro@google.com), Wei-Hung Weng (ckbjimmy@google.com), Pushmeet Kohli (pushmeet@google.com), Annalisa Pawlosky (apawlosky@google.com), Alan Karthikesalingam (alankarthi@google.com), Vivek Natarajan (natviv@google.com).

Supplementary notes

1 Glossary of terminology and concepts

- **Novel repurposing candidate.** An existing drug (a chemical compound that binds to a target), with an established safety profile, proposed for use in a disease or condition for which it is not currently approved or widely used. This approach of repurposing existing drugs differs significantly from traditional drug discovery, which involves identifying novel chemical compounds that can bind to specific targets implicated in a disease state.
- **Novel target.** A biological entity (e.g., gene, protein, pathway) not previously known as a therapeutic target for a specific disease.
- **Novel mechanistic explanation.** A newly proposed pathway, interaction, or biological process that explains a phenomenon (e.g., disease progression, antimicrobial resistance) in a way that is not explicitly described in prior literature.

2 Co-Scientist internal evaluation

2.1 The Elo rating is concordant with high quality Co-Scientist results

The Elo auto-evaluation rating is a key metric that guides the self-improvement feedback loops within Co-Scientist. Therefore, it's necessary to measure and ensure higher Elo ratings correlate with higher quality results. To assess this, we analyzed the concordance between the Elo rating and the system's accuracy on the GPQA benchmark dataset. Ideally, higher Elo ratings should correlate with a higher probability of correct answers.

The GPQA dataset is a challenging, multiple-choice question answering benchmark developed by experts in biology, physics, and chemistry [1]. To ensure that Co-Scientist Elo rating serves as an objective metric reflecting the validity and correctness of results from the system, we utilized questions within the GPQA diamond set, a subset of the GPQA dataset known for its high difficulty, framing each question as a research goal into our AI system to elicit responses. For each question, we first compared each Co-Scientist response against the ground truth answer to evaluate its correctness. Then, we categorized all generated responses across all considered questions based on their Elo rating into discrete buckets: Elo rating of 1001-1050, 1051-1100, 1101-1150, etc. in 50 point increments, until the highest rating achieved. Finally, we calculated the average accuracy for each Elo rating bucket, as the percentage of correct responses within each bucket.

We employed the underlying Gemini 2.0 models in Co-Scientist to create a reference baseline. The reference is necessary because responses within a particular Elo rating bucket are not uniformly distributed across the GPQA questions, some of which are inherently more challenging than others. This non-uniformity could introduce bias into the analysis and potentially lead to erroneous conclusions. We therefore used the reference to generate 32 responses for each GPQA question. The fraction of correct responses from Gemini 2.0 was used as a reference accuracy on that particular question. To determine reference accuracy for a specific Elo bucket, we averaged the reference accuracy of the GPQA questions that had Co-Scientist responses within that bucket. We also computed Co-Scientist accuracy on the GPQA diamond set by using the result with the highest Elo rating for each question and comparing it against the ground truth.

Our analysis using questions from the GPQA diamond set reveals a concordance between the Elo rating and averaged accuracy of generated Co-Scientist results, as depicted in Supplementary Fig. 1. By selecting the top-rated Co-Scientist result for each question, Co-Scientist achieves a top-1 accuracy of 78.4%.

2.2 Co-Scientist augmented expertise through iterative refinement

A fundamental design principle of Co-Scientist is its collaborative paradigm. Beyond structured scientific thinking and hypothesis generation, the system is engineered to integrate and build

upon human intuition. We investigated whether Co-Scientist's tournament-based evolution process could iteratively refine and quantitatively improve initial "best guess" solutions provided by human domain experts.

Using the subset of 15 highly complex biomedical research goals, human experts initially provided their best hypothetical solutions. These human-generated seeds were then introduced into the Co-Scientist tournament framework, where they underwent rounds of critique, scientific debate, and evolutionary refinement alongside autonomously generated ideas.

As illustrated in Extended Data Fig. 1, Co-Scientist demonstrated the clear capacity to enhance the experts' initial solutions over time. The Elo ratings of the combined "Human + AI" hypotheses progressively increased with scaled test-time compute. Notably, these human-seeded, AI-refined proposals initially mirrored the improvement trends of Co-Scientist's autonomously generated solutions but subsequently surpassed them. While this is a preliminary finding, it quantitatively suggests that Co-Scientist can act as a powerful amplifier, augmenting human ingenuity and accelerating the maturation of early-stage scientific ideas.

2.3 LLM-as-a-judge preference auto-evaluation

While human expert evaluation remains the absolute gold standard for assessing the scientific utility of our generated hypotheses, as detailed in the main text, manual expert review is inherently resource-intensive. To provide an orthogonal, scalable validation of Co-Scientist performance, we implemented an automated "LLM-as-a-judge" evaluation framework. We conducted a preference ranking evaluation between Co-Scientist and baseline models (Gemini 2.0 Flash Thinking Experimental 12-19, Gemini 2.0 Pro Experimental, and OpenAI o1) across the 15 expert-curated research goals. To mitigate evaluator bias, we employed a diverse panel of state-of-the-art reasoning models as independent judges: OpenAI o3-mini-2025-01-31, o1-preview-2024-09-12, Gemini 2.0 Pro Experimental, and Gemini 2.0 Flash Thinking Experimental 01-21.

As shown in Extended Data Fig. 2, outputs generated by Co-Scientist were consistently ranked as the most preferred across all four distinct LLM evaluators. Crucially, these automated preference rankings are highly concordant with the subjective assessments provided by our human clinical experts, further validating the robustness of the Co-Scientist's generated outputs.

2.4 Safety evaluation of Co-Scientist using adversarial research goals

Co-Scientist is designed to empower scientists and accelerate research. However, it's crucial to ensure the system is designed in line with robust safety principles, given the potential for misuse. This includes addressing dangerous research goals, dual-use objectives, scenarios where safe goals lead to unsafe hypotheses, misleading claims, and inherent biases. While this topic requires extensive investigation beyond the scope of this work, we employed adversarial testing strategies to conduct a preliminary analysis of the performance of classifiers built to reject dangerous or

unethical research goals. Specifically, we curated a set of 1200 adversarial research goals, ranging in complexity, across 40 biomedical and scientific topics as well as slightly over 2000 examples of safe research goals using frontier LLMs. We then evaluated whether Co-Scientist could robustly reject dangerous research goals while not precluding safe research. In this preliminary analysis, the system successfully passed all checks. Given the sensitive nature of these adversarial research goals, we will not be publicly releasing the dataset, but it can be made available upon request. Collectively, the benchmark, automated, and expert evaluations presented in this section provide compelling evidence of the system's strong capabilities.

3 Quantitative ablation and sensitivity analysis

We systematically disabled individual agents and external tools and evaluated the performance degradation via Elo rating and accuracy on the GPQA diamond dataset, and our paper QA benchmark, which is a dataset we constructed from papers published after model knowledge cutoff and where we control that the system has no access to the answer by filtering search results. We constructed this benchmark with 200 examples from recent papers published in bioRxiv after the model's knowledge cutoff. The objective of the task is to evaluate whether the model can rediscover a particular research hypothesis in the paper without having access to the ground truth. As these research ideas were already published, they serve as a ground truth of being correct and not novel. This will provide the quantitative evidence needed to assess each component's contribution.

The whole ablation study quantitatively demonstrates the advantages of including the individual agents. The experiments isolate and quantify the impact of key architectural and strategic choices within our multi-agentic Co-Scientist framework, including all agents: Generation agent, Reflection agent with/without external tool use (web literature search), Ranking agent (comparator agent), Evolution agent, Meta-review agent, and Proximity agent. We include the summarized findings of the ablation study in Supplementary Table 1.

- Generation agent
    - To quantify the contributions of the different strategies employed by our Generation agent, we analyzed their respective roles in generating correct hypotheses across the GPQA diamond dataset and our paper QA benchmark. We calculated the fractional contribution of each idea generation strategy (e.g.,"using focus areas", "exploring articles") to every correct hypothesis. Our findings indicate that a diverse set of strategies is crucial for success, with no single strategy dominating. For instance, on GPQA, strategies like "using focus areas" (13.8%) and "using generate prompt" (13.0%) were major contributors, while on our paper QA benchmark, strategies such as "using conditional hops" (2.88%) and "using raw ideas" (2.66%) showed higher utility. This analysis confirms that the multi-faceted approach of the Generation agent is effective and provides a quantitative basis for the utility of each of its underlying methods.
- Reflection agent
    - We examined the impact of the Reflection agent and its use of external search tools. We specifically performed an ablation study about novelty and correctness check with the web search functionality using our paper QA benchmark. For novelty, the agent without search incorrectly rated the ideas as highly novel, with an average score of 6.14 [95% CI: 5.93, 6.34] (i.e., very likely novel). In contrast, the agent with search without datetime cutoff correctly identified the ideas as lacking novelty since they may be found via web search, with an average score of 2.38 [95% CI: 2.1, 2.67] (i.e., trivially modified). This shows that the external

search tool use is critical for grounding the model and preventing the hallucination of novelty. Without such novelty check, it is challenging to filter out non-novel results. For correctness, the agent with search also performed better, providing higher confidence scores for these correct ideas (average score of 8.46 [95% CI: 8.2, 8.73], i.e., highly plausible) compared to the agent without search (average score of 7.4 [95% CI: 7.16, 7.64], i.e., plausible). These findings are consistent with our GPQA benchmark results, where enabling search also improved the classifier AUC for predicting correctness from 0.643 to 0.651 (with Flash 2.0, more details below). The correctness review is essential for Co-Scientist to filter out those generated outputs with incorrect results (Supplementary Fig. 2).

- Ranking agent (tournament)
  - Ranking agent / Tournament is the key component of Co-Scientist. To ensure whether having the Ranking agent (comparator agent) helps generate outputs with better correctness, we asked whether the Elo ratings and ranks derived from Ranking agent correlate with correctness of generated ideas. For this, we investigated how the average idea accuracy changes with respect to the Elo rating, and examined how the average accuracy changes with respect to the rank focusing on each question. Our study shows that the higher Elo rating ideas across all GPQA questions are on average more accurate than the lower Elo rating ideas, and the higher ranked ideas are also on average more accurate than the lower ranked ideas (Supplementary Fig. 3). The findings support that the Elo ratings and ranks generated by Ranking agent help Co-Scientist users to identify many correct, accurate ideas among all generated instances.
  - After showing Co-Scientist performance with different model versions (Gemini Flash 2.0 and 2.5), we further conducted experiments of prompt sensitivity testing different prompting strategies for Ranking agent. Specifically, we want to know how stable and accurate the Ranking agent is at the level of a single comparison given different prompting strategies. Thus, we compared the simple comparison prompt and a more sophisticated scientific debate prompt that we are using in Ranking agent. We looked at the accuracy of a single match (comparison between two ideas, where one of them is correct), and how it changes with respect to the prompt strategy (simple or debate prompt). We found that the accuracy on the 2000 general matches (we sampled ideas regardless of their Elo ratings) the simple comparison is on par with debate prompt in Flash 2.0 model. However, the scientific debate prompt yielded better performance in Flash 2.5 on the general matches, and proved significantly more effective when comparing high Elo rating top ideas (1800 matches where both sampled ideas have high Elo rating) regardless of the backend model version (Supplementary Fig. 4). This finding demonstrates the importance of the prompting strategy we used in the system.

- To further address the evaluation bias in Ranking agent, we have conducted a new experiment to analyze the stability of idea comparisons (i.e., mentioning order change, A vs. B vs. B vs. A). We examined 2000 idea matches for both Flash 2.0 and Flash 2.5 based Co-Scientist, and found that the simple prompt with Flash 2.0 exhibited stronger positional bias, or "second player advantage". In contrast, the scientific debate prompt almost eliminated this bias, producing more stable and trustworthy pairwise rankings. This finding is crucial, as it validates the robustness of our internal Elo ranking tournament, which is the core component of the system (Supplementary Fig. 5).

- Evolution agent
  - We also investigated the contribution of the Evolution agent to the quality of top-ranked ideas by comparing baseline (directly generated) and improved (iteratively refined) hypotheses. On the GPQA diamond dataset, we analyzed 134 (out of 150) questions where an improved hypothesis achieved the highest rank. We found that the improvement process boosted precision from 70.9% (for the best baseline hypothesis) to 75.4% (for the best improved hypothesis), an absolute increase of 4.5% [95% CI: -2%, 11%]. A similar analysis was conducted using our paper QA benchmark, in which each research goal was rated on a 1-10 quality score, showing that the iterative improvement process increased the average maximum score from 4.7 to 5.6, an improvement of 0.9 points [95% CI: 0.05, 1.7]. These results provide strong quantitative evidence that the Evolution agent's refinement process is a critical driver of hypothesis quality.
- Meta-review agent
  - To determine whether Meta-review agent is essential or only stylistic, we analyzed its impact on correctness reviews using the GPQA diamond evaluation task. The Meta-review agent provides a full review with structured guidance for "recurrent reviews" on top-ranked, promising ideas. We went through all ideas for GPQA questions that have both a full review (generated by Reflection agent) and a recurrent review (generated by Meta-review agent), and analyzed how good the review (based on review score) can actually predict whether the hypothesis is correct or not. We looked at how well the review scores from each of the review types can be used to classify the hypothesis into correct/incorrect and measured this using the AUC metric (with the label being the hypothesis choosing the correct answer/choice). Our results show a positive impact of using the Meta-review agent. On the GPQA dataset (1680 ideas, run with Gemini Flash 2.5), the Area Under the Curve (AUC) for correctly classifying ideas improved from 0.629 for standard full reviews to 0.634 for recurrent reviews guided by the Meta-review agent. This benefit was also seen on our paper QA benchmark (600 ideas, run with Gemini Flash 2.5). where the AUC increased from 0.521 to 0.597, and the correlation with idea quality scores from 0.101 to 0.226. This

demonstrates that Meta-review agent provides a measurable benefit to the system's critical evaluation capabilities, particularly for complex, open-ended research questions.

- Proximity agent
  - We quantified the performance of our Proximity agent, which clusters hypotheses based on semantic similarity (scored 1-5). Analysis of our paper QA benchmark revealed a correlation between proximity score and the difference in quality scores of hypothesis pairs. As shown in the heatmap, pairs with high proximity scores (e.g., 4-5) consistently had small differences in their quality scores, whereas pairs with low proximity scores (e.g., 1-2) exhibited a much wider range of quality score differences (Supplementary Fig. 6). This validates that the Proximity agent effectively groups semantically similar ideas, which is essential for ensuring a diverse and comprehensive exploration of the research problem space.

4 Clinical expert evaluation of drug repurposing proposals in NIH Specific Aims format

To assess the translational potential of Co-Scientist generated repurposing hypotheses, we reformatted them into NIH-style Specific Aims pages, a standard format familiar to clinical researchers for evaluating research merit. Three representative examples of the Specific Aims page were listed in section 4.1 below.

The NIH Specific Aims Page format follows a standard structure, including disease description, unmet need, proposed solutions, and specific aims. This format was selected because it provides a standardized framework that is widely recognized in the research community, allowing for systematic presentation of complex scientific topics in a manner that facilitates rigorous peer review and enables efficient assessment of scientific merit. The specific aims, which outline the overarching goal, hypothesis, and rationale, requires extensive scientific expertise, comprehensive literature analysis, and robust domain knowledge. We generated cancer drug repurposing hypotheses from Co-Scientist in the format of NIH Specific Aims Page with additional constrained decoding and self-critique stages to ensure format consistency. The resulting format contextualizes proposed repurposing candidates within known mechanisms based on current literature and then extrapolates to a new disease state. An expert oncologist methodically evaluated and excluded hypotheses that were deemed clinically implausible or had limited potential for successful translation, as well as those falling outside the expertise of the assembled specialist evaluators. This initial screening process employed multiple evidence-based criteria including: (1) pharmacological mechanism incompatibility with tumor biology; (2) unfavorable pharmacokinetic profiles for oncological applications; (3) prohibitive toxicity profiles documented in prior clinical use; (4) confounding effects where apparent survival benefits were attributable to improved management of treatment-related morbidity rather than direct anti-neoplastic activity; and (5) insufficient preclinical evidence supporting antitumor efficacy at clinically achievable concentrations. For example, bisphosphonate agents like pamidronate, while associated with improved outcomes in observational studies of patients with bone metastases, were excluded after critical evaluation revealed their benefits stemmed primarily from reduction of skeletal-related events (such as pathological fractures, spinal cord compression, and bone pain requiring radiation) rather than from disease modifying activity of the drug-candidate.

Nine board-certified hematologists and oncologists from a single institution, including six domain-specific oncologists specializing in gastrointestinal (GI), breast, gynecologic (GYN), thoracic, and genitourinary (GU) cancers, and three general hematologists and oncologists, with an average of 6.7 years of clinical experience, were divided into two groups and evaluated 78 unique drug repurposing hypotheses presented in the NIH Specific Aims Page format (for specific indication distribution and counts, see below section 4.2).

Two groups of expert raters evaluated the generated Specific Aims based on a modified NIH grant proposal evaluation rubric, consisting of 15 axes focusing on (1) importance of

research (significance and innovation) and (2) approach (rigor and feasibility). The raters indicated their agreement level using a five-point scale: “Strongly Agree”, “Agree”, “Neutral”, “Disagree”, and “Strongly Disagree”. For each axis, we included several questions covering different aspects of the NIH evaluation criteria. The evaluation rubric is further detailed in below 4.3. Specifically, we ask raters to focus on evaluating the clinical relevance and potential for clinical translation, and not for translational capacity or the design of clinical trials.

We observed that the rating correlation between two independent group ratings is at a high and reliable degree of agreement with the Spearman’s rho of 0.745 ($p < 0.001$), and both groups of expert raters consistently assigned high ratings (“Strongly Agree” or “Agree”) to the Specific Aims proposed by Co-Scientist across various evaluation criteria (Supplementary Fig. 7). Of note, the favorable assessments of Co-Scientist-generated hypotheses may be partially attributed to expert pre-screening, wherein a clinician eliminated non-viable candidates prior to expert evaluation. Three examples of generated Specific Aims and their respective expert review ratings are detailed in below section 4.1. Interestingly, Co-Scientist also independently proposed a dihydroorotate dehydrogenase (DHODH)-focused Specific Aim in AML that parallels findings from a recently published T-ALL study [2], despite the referenced work not directly involving AML.

The generated Specific Aims were assessed by nine clinical hematologists and oncologists from a single-center, which might bias the interpretation of the evaluation results, as it may introduce institutional perspectives shaped by local practice patterns, clinical experiences, and research frameworks unique to that setting. While some Specific Aims may be supported by preclinical data, it is important to note that none of the proposed drug candidates have undergone randomized phase III clinical trials necessary to establish efficacy and secure regulatory approval for repurposing to a new indication.

4.1 Examples of Co-Scientist generated Specific Aims with expert evaluation

4.1.1 Example 1: Givosiran for AML

Disease Description

Acute myeloid leukemia (AML) is an aggressive hematological malignancy with an incidence rate of approximately 4.3 per 100,000 persons per year in the United States, predominantly affecting older adults. AML arises from a complex interplay of genetic mutations, epigenetic alterations, and dysregulated signaling pathways, disrupting normal hematopoiesis by blocking myeloid differentiation and promoting uncontrolled blast proliferation. This leads to bone marrow failure, infections, bleeding, and other life-threatening complications. The current standard of care includes intensive chemotherapy, often combined with targeted therapies or hematopoietic stem cell transplantation. However, these treatments are often associated with significant toxicity, high relapse rates, and limited efficacy in certain patient populations, particularly in relapsed/refractory or high-risk AML.

Unmet Need

Despite advancements, significant unmet needs persist in AML treatment. Current therapies often cause severe side effects, particularly in older or frail patients, limiting their tolerability and effectiveness. Relapse rates remain high, and treatment options for relapsed/refractory AML are limited and often less effective. Many patients fail to achieve complete remission or experience only short-lived responses, underscoring the urgent need for novel, less toxic, and more effective therapies, especially for patients with relapsed/refractory or high-risk disease.

Proposed Solution

Givosiran sodium is an RNA interference (RNAi) therapeutic approved for acute hepatic porphyria (AHP). It targets aminolevulinate synthase 1 (ALAS1) mRNA, the rate-limiting enzyme in heme biosynthesis, reducing the production of heme precursors δ-aminolevulinic acid (ALA) and porphobilinogen. This prevents the accumulation of neurotoxic heme intermediates in AHP.

Repurposing givosiran for AML stems from the crucial role of heme biosynthesis in AML pathogenesis. Several studies suggest that disrupting heme biosynthesis offers a therapeutic advantage in AML. The proposed approach addresses the unmet need by targeting AML cells dependent on increased heme biosynthesis, particularly those with MYCN overexpression [1]. Modulating heme levels could influence oxidative stress [5, 6], apoptosis [1, 2], and drug sensitivity in AML [2]. Givosiran's ALAS1 inhibition aligns with this approach, offering a novel therapeutic strategy.

We hypothesize that givosiran, by inhibiting ALAS1 and reducing heme biosynthesis, will suppress AML cell growth and survival, particularly in those with upregulated heme biosynthesis. This is supported by preclinical evidence demonstrating that altering heme levels

impacts AML cell proliferation, apoptosis, and drug sensitivity [2]. Abstract [1] suggests that elevated heme biosynthesis in MYCN-driven AML is a therapeutic vulnerability. Reducing ALA and porphobilinogen accumulation via givosiran can mitigate oxidative stress [6], a factor implicated in AML progression.

Overall, our goal is to evaluate the efficacy and safety of givosiran as a novel therapeutic strategy for AML by exploiting the crucial role of heme biosynthesis in its pathogenesis and the drug's ability to modulate heme levels and downstream effects on AML cell proliferation, survival, and drug sensitivity.

Specific Aims 1

Overarching goal: Determine the anti-leukemic activity of givosiran in MYCN-driven AML models.

Hypothesis: Givosiran treatment will decrease the viability and proliferation of MYCN-overexpressing AML cells in vitro and reduce tumor growth in MYCN-driven AML xenograft mouse models.

Reasoning: MYCN-driven AML frequently exhibits upregulated heme biosynthesis [1], creating a potential dependency on this pathway. Givosiran, by inhibiting ALAS1, could disrupt this dependency, leading to decreased heme and growth inhibition. This is supported by preclinical data showing that inhibiting heme biosynthesis impacts AML cell growth and survival [1, 2].

Specific Aims 2

Overarching goal: Elucidate the impact of givosiran on oxidative stress and drug sensitivity in AML.

Hypothesis: Givosiran treatment will modulate oxidative stress levels and enhance the cytotoxic effects of standard AML chemotherapeutics (e.g., cytarabine) in AML cell lines and primary patient samples.

Reasoning: Heme plays a role in oxidative stress regulation, and its modulation by givosiran could influence AML cell chemosensitivity. Abstracts [5, 6] highlight oxidative stress in AML and the potential for ALA accumulation to contribute to it. By reducing ALA and heme, givosiran could alter reactive oxygen species (ROS) levels and potentially sensitize AML cells to chemotherapy-induced death.

Specific Aims 3

Overarching goal: Characterize the safety and tolerability of givosiran in preclinical AML models, focusing on its impact on liver function.

Hypothesis: Givosiran treatment will be well-tolerated in AML mouse models, with minimal adverse effects on liver function and drug metabolism, at doses that effectively inhibit ALAS1 and reduce heme biosynthesis.

Reasoning: Given givosiran's hepatic target (ALAS1), evaluating its safety profile in AML is crucial. Abstracts [7, 8] highlight the clinical significance of liver function in AML patients. This

aim will assess potential hepatotoxicity and drug-drug interactions, ensuring safe translation to clinical trials. We will evaluate relevant liver function markers and givosiran's impact on standard AML chemotherapeutic metabolism.

Pilot Evaluation

A pilot study in a human AML xenograft mouse model will assess givosiran's in vivo efficacy and safety. Givosiran will be administered at various doses, monitoring tumor growth, survival, and liver function. The primary endpoint will be tumor growth inhibition. Secondary endpoints include survival, changes in heme levels, oxidative stress markers, and liver function tests. Existing safety data from givosiran's use in AHP will inform dose selection and monitoring. While givosiran is approved for AHP, its use in AML requires an Investigational New Drug (IND) application to the FDA before clinical trials. Existing safety data might facilitate a streamlined review process.

Articles

[1] Upregulated heme biosynthesis, an exploitable vulnerability in MYCN-driven leukemogenesis Summary: This study
demonstrates increased heme biosynthesis in MYCN-driven leukemias, suggesting it as a therapeutic target. Relevance:
Supports the rationale for targeting heme biosynthesis in MYCN-driven AML and provides a rationale for the proposed
mechanism of action of givosiran in this context.

[2] Systematic Dissection of the Metabolic-Apoptotic Interface in AML Reveals Heme Biosynthesis to Be a Regulator
of Drug Sensitivity Summary: This study highlights the role of heme biosynthesis in regulating apoptosis and drug
sensitivity in AML. Relevance: Supports the potential for givosiran to impact AML cell survival and treatment response
by modulating heme biosynthesis.

...

[8] Acute myeloid leukemia with hepatic infiltration presenting as obstructive jaundice Summary: This abstract shows
liver dysfunction in AML and the need to consider liver health in treatment. Relevance: Reinforces the importance of
monitoring liver function in AML patients treated with givosiran, further supporting the rationale for Aim 3.

4.1.2 Example 2: Selinexor monotherapy for colon cancer

Disease Description

Colon adenocarcinoma (COAD) is a significant public health concern, with an estimated incidence rate of over 1.9 million new cases and 935,000 deaths globally in 2020. COAD arises from the epithelial lining of the colon and is driven by a complex interplay of genetic mutations (e.g., APC, KRAS, BRAF, TP53) and epigenetic alterations, leading to uncontrolled cell proliferation, impaired apoptosis, and chronic inflammation. These molecular changes disrupt crucial cellular pathways like Wnt/β-catenin signaling, cell cycle control, and DNA damage repair, ultimately driving tumor progression. COAD typically progresses through stages, from localized polyps to invasive tumors with potential for metastasis. Current standard of care involves surgery, chemotherapy, radiation therapy, and targeted therapies, but treatment resistance and recurrence remain major challenges, highlighting the need for new therapeutic approaches.

Unmet Need

Current COAD treatments have limitations, including acquired resistance to chemotherapy, significant toxicities, and incomplete responses in advanced disease. Patients often experience diminished quality of life due to treatment side effects and disease burden. High recurrence rates and limited effective treatment options after progression contribute to poor long-term outcomes. This unmet need underscores the urgency for novel therapeutic strategies that can overcome resistance, improve response rates, and minimize toxicity, ultimately extending survival and enhancing quality of life for COAD patients.

Proposed Solution

Selinexor, a first-in-class selective inhibitor of nuclear export (SINE), is currently approved for the treatment of multiple myeloma and diffuse large B-cell lymphoma. It specifically targets XPO1 (Exportin 1), a key protein responsible for the nuclear export of tumor suppressor proteins, oncoproteins, and RNA. By binding to XPO1, Selinexor blocks the nuclear export of these molecules, leading to their accumulation in the nucleus and restoration of tumor suppressor function, cell cycle arrest, and apoptosis induction.

Repurposing Selinexor for COAD is rationally supported by its mechanism of action and the molecular characteristics of the disease. Overexpression of XPO1 is common in various cancers, including COAD [1, 2, 6]. Selinexor inhibits XPO1, preventing the nuclear export and restoring the function of key tumor suppressors (p53, RB, FOXO, APC) frequently dysregulated in COAD [2, 3, 4, 5]. Furthermore, Selinexor can suppress constitutively activated NF-κB signaling, a driver of chronic inflammation and tumor progression in COAD, by blocking IκB export and increasing its nuclear accumulation [7, 8]. These mechanisms align with key aspects of COAD pathogenesis and offer opportunities for therapeutic intervention.

We hypothesize that Selinexor will effectively inhibit COAD cell growth and enhance apoptosis by restoring tumor suppressor function and modulating crucial signaling pathways. Selinexor has shown anti-tumor activity in solid tumors in a Phase I trial [3], with observations of nuclear accumulation of tumor suppressor proteins [3, 6]. While not specific to COAD, these findings, coupled with evidence of Selinexor's efficacy in other cancers driven by XPO1 overexpression [3, 6], suggest that a similar mechanism could be effective in COAD. Further supporting our hypothesis, XPO1 overexpression has been linked to NF-κB activation and increased proliferation in COAD [7]. Overall, our goal is: to demonstrate that Selinexor's XPO1 inhibitory activity can effectively target key oncogenic drivers and restore tumor suppressor functions in COAD, ultimately leading to tumor growth inhibition and improved patient outcomes.

Specific Aims 1

Overarching goal: Determine the in vitro efficacy of Selinexor in inhibiting COAD cell growth and inducing apoptosis.

Hypothesis: Selinexor treatment will significantly reduce the viability and increase apoptosis in a panel of COAD cell lines, including those with varying genetic backgrounds (e.g., APC, KRAS, TP53 mutations).

Reasoning: Selinexor's inhibition of XPO1 leads to nuclear accumulation of tumor suppressors like p53, a key regulator of apoptosis [2, 6]. Restoration of p53 function and suppression of NF-κB, a pro-survival pathway [7, 8], are expected to induce apoptosis in COAD cells. We will evaluate this hypothesis using cell viability and apoptosis assays in diverse COAD cell lines to assess the impact of genetic background on Selinexor's efficacy.

Specific Aims 2

Overarching goal: Elucidate the mechanisms by which Selinexor inhibits COAD cell growth, focusing on XPO1-mediated restoration of tumor suppressor function.

Hypothesis: Selinexor treatment will increase nuclear accumulation of p53, RB, and FOXO proteins and decrease nuclear export of IκB, leading to cell cycle arrest and decreased NF-κB activity in COAD cells.

Reasoning: Selinexor blocks XPO1, preventing nuclear export of key tumor suppressors (p53, RB, FOXO) [2, 4, 5] and the NF-κB inhibitor IκB [7, 8]. Increased nuclear localization of these proteins should restore their growth regulatory functions, leading to cell cycle arrest and reduced NF-κB-driven proliferation. We will evaluate this hypothesis using immunofluorescence and western blotting to assess protein localization and activity.

Specific Aims 3

Overarching goal: Characterize the potential synergistic effects of Selinexor in combination with standard-of-care chemotherapies for COAD.

Hypothesis: Combination treatment with Selinexor and 5-fluorouracil (5-FU) will synergistically reduce COAD cell viability compared to either treatment alone.
Reasoning: XPO1 inhibition can sensitize cancer cells to chemotherapy [9]. Combining Selinexor with 5-FU, a common COAD chemotherapy, may enhance cellular stress and improve treatment response. We will test this hypothesis using cell viability assays and investigate the underlying mechanisms of synergy.

Pilot Evaluation
A pilot study will evaluate Selinexor's efficacy in a patient-derived xenograft (PDX) model of COAD. The primary objective is to determine the effect of Selinexor on tumor growth. The study will utilize an open-label, single-arm design with escalating Selinexor doses in established COAD PDX models. Inclusion criteria: established COAD PDX models. Exclusion criteria: none. Primary endpoint: tumor volume change. Secondary endpoints: changes in biomarkers (p53, Ki67, NF-κB) within the tumor. Selinexor's established safety profile in other cancers provides a basis for evaluating its safety in this new indication. Potential for expedited review through existing regulatory pathways will be explored.

Expert rating

1. Strongly Agree (unmet clinical needs)
2. Agree (bridges therapeutic gap)
3. Strongly Agree (scientifically rigorous rationale)
4. Agree (integrates prior studies)
5. Agree (avoids over-extrapolation)
6. Strongly Agree (clear hypotheses and methods)
7. Strongly Agree (clearly stated aims)
8. Agree (path to clinical application)
9. Agree (well-defined endpoints)
10. Agree (meaningful pre-clinical experiments)
11. Agree (translational component)
12. Strongly Agree (avoids inaccuracies)
13. Strongly Agree (evidence-based assumptions)
14. Agree (originality and terminology)
15. Strongly Agree (clear writing and organization)

4.1.3 Example 3: Lapatinib for colon cancer

Disease Description

Colon adenocarcinoma (COAD) is a significant public health concern, with an estimated incidence rate of over 150,000 new cases annually in the United States [19]. COAD arises from the epithelial lining of the colon, and its pathogenesis involves a complex interplay of genetic and environmental factors. Key molecular mechanisms include mutations in genes such as APC, KRAS, BRAF, and PIK3CA, as well as dysregulation of signaling pathways like Wnt, RAS/RAF/MEK/ERK, and PI3K/AKT/mTOR [8, 9, 12]. These alterations drive uncontrolled cell proliferation, evade apoptosis [14], promote angiogenesis [15], and ultimately lead to tumor growth, invasion, and metastasis [16]. The disease typically progresses through a series of stages, from localized tumors to regional lymph node involvement and distant metastasis. Current standard of care involves surgery, chemotherapy, and targeted therapies such as anti-EGFR antibodies. However, these treatments have limitations, including acquired resistance and significant toxicity.

Unmet Need

A major unmet need in COAD treatment is the development of effective therapies for patients who progress on or are refractory to standard treatments, particularly those with resistance to anti-EGFR therapy [2]. Despite available therapies, many patients experience disease recurrence and metastasis, leading to poor outcomes and diminished quality of life. There's a critical need for new therapeutic options that can overcome resistance mechanisms, improve response rates, and offer better tolerability profiles. Specifically, addressing resistance driven by KRAS mutations [10] and exploring alternative therapeutic targets remains crucial.

Proposed Solution

Lapatinib is an orally available small molecule tyrosine kinase inhibitor currently approved for use in combination with capecitabine for the treatment of HER2-positive metastatic breast cancer. It reversibly inhibits the intracellular tyrosine kinase domains of both EGFR (ErbB1) and HER2 (ErbB2), thereby blocking downstream signaling cascades, including RAS/RAF/MEK/ERK and PI3K/AKT/mTOR. This inhibition leads to decreased cell proliferation and increased apoptosis.

Repurposing lapatinib for COAD is rationalized by the shared ErbB signaling pathway between breast cancer and a subset of COAD. EGFR is commonly overexpressed in COAD [1], and while HER2 overexpression is less frequent than in breast cancer, it occurs in a clinically relevant subset [3, 17] and is associated with resistance to anti-EGFR therapy [2]. Lapatinib can directly inhibit both EGFR and HER2, potentially disrupting crucial oncogenic signaling [13, 14] including PLCγ [7].

We hypothesize that lapatinib can effectively inhibit ErbB signaling in COAD, leading to decreased cell proliferation, increased apoptosis, and suppression of metastasis. Preclinical

studies demonstrate synergistic antitumor activity of lapatinib with HDAC inhibitors in COAD models [6], and lapatinib has also been shown to sensitize COAD cells to TRAIL-induced apoptosis via an off-target mechanism [5]. Studies have also investigated HER2 as a therapeutic target in CRC, especially after failure of anti-EGFR therapy [2, 18]. Although lapatinib as a single agent has shown limited efficacy in unselected CRC populations, this could be attributed to the heterogeneity of COAD and the presence of resistance mechanisms like KRAS mutations. We anticipate that patient stratification based on molecular profiles will identify subgroups that derive greater benefit.

Overall, our goal is: to demonstrate the efficacy of lapatinib in specific molecular subtypes of COAD, potentially in combination with other targeted therapies, to address the unmet need for new treatment options, particularly in patients resistant to standard therapies.

Specific Aims 1

Overarching goal: To determine the efficacy of lapatinib in inhibiting HER2/EGFR signaling and suppressing cell proliferation in a panel of well-characterized COAD cell lines.
Hypothesis: Lapatinib will inhibit cell proliferation in HER2-amplified/overexpressing and/or EGFR-overexpressing COAD cell lines.
Reasoning: Lapatinib directly inhibits both HER2 and EGFR, key drivers of cell proliferation in a subset of COAD. Inhibition of these receptors should lead to reduced downstream signaling through the RAS/RAF/MEK/ERK and PI3K/AKT/mTOR pathways, ultimately suppressing cell growth [6].

Specific Aims 2

Overarching goal: To elucidate the impact of lapatinib on apoptosis and key signaling pathways in COAD models.
Hypothesis: Lapatinib will induce apoptosis and modulate key signaling pathways (RAS/RAF/MEK/ERK, PI3K/AKT/mTOR, and PLCγ) in COAD cell lines, especially those with HER2/EGFR alterations.
Reasoning: Inhibition of HER2/EGFR by lapatinib disrupts pro-survival signaling, potentially promoting apoptosis [14]. Additionally, lapatinib can indirectly modulate downstream pathways [4, 7], even demonstrating synergistic effects with other agents [6]. This aim will explore the extent of this modulation in various COAD subtypes.

Specific Aims 3

Overarching goal: To identify predictive biomarkers for lapatinib response and explore potential combination strategies in COAD.
Hypothesis: A combination of lapatinib with a MEK inhibitor will enhance antitumor activity in KRAS-mutant COAD cell lines compared to lapatinib alone.

Reasoning: KRAS mutations are a known mechanism of resistance to EGFR/HER2 targeted therapies [10]. Combining lapatinib with a MEK inhibitor may overcome this resistance by blocking the downstream MAPK pathway activation, leading to enhanced antitumor efficacy.

Pilot Evaluation
A pilot study will be conducted to evaluate the safety and preliminary efficacy of lapatinib in patients with HER2-positive metastatic COAD who have progressed on standard therapy. This open-label, single-arm study will enroll 15-20 patients with confirmed HER2 overexpression/amplification and KRAS wild-type status. Key exclusion criteria will include prior treatment with lapatinib and significant comorbidities. The primary endpoint will be progression-free survival, and secondary endpoints will include overall response rate, duration of response, and safety. Lapatinib's known safety profile from its use in breast cancer will inform the monitoring plan. Expedited regulatory review may be possible due to lapatinib's existing approval.

Articles
[1] Targeting the EGFR signalling pathway in metastatic colorectal cancer
Summary: Details the importance of EGFR signaling in CRC and how targeting it is a crucial therapeutic strategy.
Relevance: Provides context for lapatinib's action as it targets EGFR.
[2] HER2 as an Emerging Oncotarget for Colorectal Cancer Treatment After Failure of Anti-Epidermal Growth Factor
Receptor Therapy
Summary: Discusses HER2 as a target in CRC, especially in the context of anti-EGFR therapy resistance.
Relevance: Directly relevant to lapatinib's mechanism and potential in COAD.
...
[19] United States Cancer Statistics
Summary: Provides official U.S. cancer incidence and mortality statistics.
Relevance: Source of epidemiological data for colon adenocarcinoma.

Expert rating
1. Strongly Agree (unmet clinical needs)
2. Agree (bridges therapeutic gap)
3. Strongly Agree (scientifically rigorous rationale)
4. Strongly Agree (integrates prior studies)
5. Strongly Agree (avoids over-extrapolation)
6. Strongly Agree (clear hypotheses and methods)
7. Strongly Agree (clearly stated aims)
8. Strongly Agree (path to clinical application)

9. Agree (well-defined endpoints)
10. Neutral (meaningful pre-clinical experiments)
11. Strongly Agree (translational component)
12. Strongly Agree (avoids inaccuracies)
13. Strongly Agree (evidence-based assumptions)
14. Strongly Agree (originality and terminology)
15. Agree (clear writing and organization)

Of note, the aforementioned examples were generated by Co-Scientist without being restricted to a predefined subset of literature and were not filtered for the presence of preclinical evidence or prior evaluation in multi-drug regimens.

4.2 Specific Aims count distribution (Supplementary Table 2)

4.3 Specific Aims evaluation rubric

To rigorously assess Co-Scientist generated repurposing hypotheses, we developed a pilot evaluation framework in collaboration with two oncologists at a US institution. The framework was inspired by established grant review criteria, particularly the NIH Specific Aims evaluation axes, but adapted to address the unique characteristics of AI-generated proposals. Traditional grant review criteria assume that proposals originate from domain experts with deep tacit knowledge and access to recent unpublished findings. However, LLM-based systems operate differently, they synthesize publicly available information but lack the experiential knowledge and awareness of cutting-edge unpublished work that human researchers possess. Therefore, our framework prioritizes assessment of logical consistency, appropriate citation and integration of published scientific literature, and factual accuracy, rather than assuming the depth of expertise expected from human investigators.

The framework evaluates proposals across two domains totaling 15 criteria. The first domain, clinical significance (5 criteria), assesses whether proposals address genuine unmet clinical needs, demonstrate understanding of the current therapeutic landscape, and provide sound scientific rationale for the repurposing strategy. These criteria evaluate how effectively the AI system identifies clinically relevant problems and synthesizes available evidence to justify therapeutic hypotheses. The second domain, scientific rigor and methodology (10 criteria), evaluates the technical quality of proposals, including clarity of hypothesis formation, appropriateness of proposed experimental approaches, quality of preclinical study design, definition of clinical endpoints, and overall translational potential. This domain pays particular attention to LLM-specific concerns, such as factual accuracy of cited literature and the presence of hallucinated or fabricated content, which are the issues not typically relevant in human-authored proposals.

Each criterion was rated using a 5-point Likert scale (Strongly Disagree, Disagree, Neutral, Agree, Strongly Agree), allowing quantitative assessment across all evaluated proposals. Evaluations were conducted independently by two separate groups of expert hematologist-oncologists to ensure rigorous assessment.

We emphasize that this represents a pilot framework developed for initial exploration of AI-generated hypothesis evaluation. It is not intended to replicate the comprehensive depth of full grant review processes, which typically include detailed assessment of statistical power calculations, complex experimental design elements, budget justification, and investigator qualifications. Omitted axes of quality could be addressed in future work, including more granular criteria for experimental design such as power calculation review and specific study design methodologies. If this framework were to be developed into a validated assessment instrument, it would require substantial additional research, including formal evaluation of inter-rater and intra-rater reliability, construct validity, criterion validity, and correlation with

established benchmarks. The current framework serves as a starting point for systematically evaluating whether AI-generated biomedical hypotheses meet the standards expected by clinical researchers and could guide future therapeutic investigation. The complete evaluation framework with specific criteria for each domain is presented below.

- Significance and innovation (5 questions)
    - The proposal adequately identifies significant unmet clinical needs that could be addressed through drug repurposing of this pharmacological agent.
    - The proposal effectively bridges an important gap in the current therapeutic landscape for the target disease by repurposing this drug.
    - The proposal presents a scientifically rigorous rationale for repurposing the drug, grounded in current evidence and literature.
    - The scientific background integrates relevant prior studies and preliminary data to support the proposed drug repurposing.
    - The proposal avoids over-extrapolation or speculative conclusions beyond the supporting evidence.
- Rigor and feasibility (10 questions)
    - Does each Specific Aim have a clear hypothesis, and specific methodological approaches?
    - Are the Specific Aims clearly stated and logically organized?
    - There is a clear path from the proposed research to clinical application, including consideration of necessary pre-clinical and clinical studies.
    - The proposal includes well-defined and clinically relevant endpoints.
    - The pre-clinical experiments are designed to yield meaningful insights into the drug's potential efficacy and safety in its new therapeutic role.
    - The proposal includes a well-described translational component to assess the drug's efficacy in its new indication.
    - The proposal avoids factual inaccuracies or hallucinations, presenting information that is accurate and reliable.
    - The assumptions made within the proposal regarding the drug's efficacy, mechanism, and potential outcomes are grounded in current scientific evidence.
    - The proposal demonstrates originality in thought and approach, employs precise and appropriate scientific terminology and avoids ambiguous or generic statements.
    - The proposal is clearly written, with well-organized sections and a logical flow of ideas that enhances comprehension and ensures the aims are easily understood.

5 Additional information for drug repurposing evaluation

5.1 Co-Scientist suggests plausible drug repurposing candidates as rated by computational biology analyses and experts

We constrained Co-Scientist to explore potential repurposing hypotheses from a curated list of 2300 approved drugs across 34 cancer types. The list, along with their corresponding abbreviations, was constructed based on a curated text string derived from the TCGA project (Supplementary Table 3). This list includes 10 rare cancers.

To achieve this, we modified the prompts used in the Generation agent and Ranking agent stages to ensure hypothesis generation in this constrained search space; however, the core Co-Scientist logic remained unchanged. When formulating the research goal for Co-Scientist, we explicitly emphasized the following preferences related to drug repurposing:

- Elucidate the known mechanisms of action and impacted biological pathways of the drug.
- Identify potential diseases or cancer types that could be treatment targets for the drug.
- Explain the potential mechanisms by which the drug could exert therapeutic effects.
- Propose alternative mechanisms of action through which the drug might function in the proposed therapeutic context.
- Identify the diseases / cancers for which the drug is currently approved.
- List the most promising disease / cancer type candidates for repurposing.
- Discuss prior research and challenges associated with repurposing the drug.

We used Co-Scientist to generate drug repurposing proposals for 34 different cancer types from a subset of 2300 drugs curated with the Open Targets Platform (https://platform.opentargets.org/downloads). The proposal for each drug candidate includes a generated hypothesis, a review of the hypothesis, and a description of the possible mechanism of action.

For each drug-cancer pair, we also extracted the Cancer Dependency Map (DepMap) probability of dependency ("DepMap score") [3,4]. DepMap (Cancer Dependency Map) is a project and a publicly available database that focuses on identifying genetic vulnerabilities in cancer cells. By systematically perturbing genes in a large panel of cancer cell lines using techniques like CRISPR and observing the resulting phenotypic effects, DepMap constructs a comprehensive map of genetic dependencies for different cancers. This resource can be leveraged to identify promising drug targets for cancer therapies. Specifically, DepMap generates perturbational data from CRISPR screens that are subsequently used to calculate DepMap dependency scores, representing the probability of gene essentiality for a given cancer cell line. A high DepMap score indicates a high probability that a gene is required for cell survival and proliferation, i.e., it represents the probability of essentiality for a gene in a given cancer cell line.

We utilized the pre-computed DepMap dependency probabilities made available with the Q2 2024 data release of DepMap to construct a rapid, simplified sanity check of Co-Scientist's hypotheses. As part of this sanity check, for each drug-cancer hypothesis generated by Co-Scientist, we extracted the DepMap score for the known target gene associated with the drug in the relevant DepMap cancer cell line models corresponding to the hypothesis.

We ranked all drug-cancer pairs using a combined metric of Co-Scientist review score (ranging from 1 to 5) and the DepMap score (ranging from 0.0 to 1.0). To prioritize the most relevant hypotheses for expert review, we selected only pairs where Co-Scientist review score ≥4 and the DepMap score ≥ 0.99. Although this filter on the DepMap scores does not explicitly exclude common-essential or pan-essential genes, we opted for this simple, minimal filtering criterion on the DepMap score, since our use of the DepMap score in this context functions only as a rapid sanity check on Co-Scientist.

We observed a significant correlation between the DepMap scores and Co-Scientist scores, such that drug-cancer pairs scored highly by Co-Scientist also tended to have higher DepMap scores in the relevant cancer lines. Candidates selected for expert review were required to meet stringent criteria, exhibiting both Co-Scientist review score ≥ 4 and a DepMap score ≥ 0.99 (Supplementary Fig. 8). Finally, the expert oncologists reviewed the top-ranked drug-cancer pairs, provided feedback, and selected promising repurposing candidates for *in vitro* wet-lab validation (Supplementary Note 5.5, 5.6).

5.2 AML cell line selection rationale and mechanistic interpretation of drug sensitivities

Cell line selection was guided by several key principles to ensure experimental rigor and clinical relevance. First, we prioritized genetic diversity to model AML heterogeneity observed in patients. This included cell lines harboring distinct driver mutations as well as those representing different AML subtypes. For example, when evaluating compounds targeting specific pathways such as FLT3, we included both FLT3-ITD positive lines (MV4-11, MOLM-13) and FLT3 wild-type lines (OCI-AML3, HL-60) to distinguish on-target from off-target effects and establish structure-activity relationships across genetic contexts. Similarly, we included NOMO-1, a cell line harboring the t(9;11)(p22;q23) chromosomal translocation resulting in KMT2A-MLLT3 (MLL-AF9) fusion, to represent AML with KMT2A rearrangements, a clinically relevant subtype with poor outcomes.

Second, where available, we considered isogenic cell line pairs, genetically identical except for a single targeted gene, as these provide definitive validation of on-target drug effects. While such paired lines are not universally available for all mutations of interest, they offer powerful confirmatory evidence when obtainable or when custom-engineered for specific validation studies.

Third, comprehensive molecular characterization informed both cell line selection and interpretation. Each selected line represents defined molecular subtypes observed clinically, with well-characterized baseline profiles including mutational status, expression signatures, and

signaling pathway activation states. This baseline characterization enables prediction of how gene expression and pathway activity should change following treatment based on each drug's known mechanism of action and hypothesized repurposing mechanism, facilitating identification of pharmacodynamic biomarkers.

Finally, while immortalized cell lines provide reproducible platforms for initial screening, we recognized that lead candidates require validation in primary patient samples to confirm clinical translatability. Cell lines were therefore selected not only for their experimental utility but also for their molecular similarity to patient subpopulations, informing subsequent validation strategies in patient-derived specimens.

The complete panel of selected cell lines with their molecular characteristics is provided in Supplementary Table 4.

Case study: Mechanistic rationale for differential KIRA6 sensitivity. Building upon this selection rationale, the phenotypic and cytogenetic diversity of our cell line panel provided crucial mechanistic insights during our *in vitro* validations. Specifically, when evaluating the novel candidate KIRA6 (an IRE1α inhibitor), we observed highly variable sensitivities that closely tracked with the differentiation states of the AML cell lines.

As noted in the main text, KIRA6 demonstrated heightened susceptibility observed in KG-1a cells, which retain a primitive $CD34^+$, stem-like phenotype. In contrast, MOLM-13 (FAB M5a) and HL-60 (FAB M2) represent more differentiated myeloid lineages with preserved capacity for terminal maturation. Because the IRE1α–XBP1 axis supports self-renewal programs in hematopoietic and pre-leukemic stem cells, the preferential sensitivity of KG-1a suggests that IRE1α inhibition may disproportionately affect primitive AML populations. The 18-fold separation between the KG-1a IC_{50} and that of the normal lymphoblastoid TK6 line further raises the possibility of a therapeutic window, although this requires validation in primary AML samples and *in vivo* models to define selectivity and clinically actionable exposures. NOMO-1's intermediate sensitivity (IC_{50} = 144 nM) may reflect its retained differentiation plasticity, as these KMT2A-MLLT3-rearranged cells remain responsive to terminal monocytic differentiation cues such as TPA, distinguishing them from primitive $CD34^+$ KG-1a cells and suggesting reduced dependence on IRE1α-mediated proteostasis for self-renewal. This positions NOMO-1 between highly primitive, IRE1α-dependent AML (KG-1a) and fully differentiated, IRE1α-independent lineages (MOLM-13, HL-60).

Overall, these data validate our cell line selection strategy and suggest that IRE1α blockade may be most effective in AML subsets enriched for stem-like transcriptional states, such as therapy-associated myeloid neoplasms, while resistance in more differentiated leukemias warrants additional mechanistic investigation.

5.3 Details of the selected repurposed drugs

Binimetinib is an inhibitor of MEK1 and MEK2, key kinases in the RAS–RAF–MEK–ERK signaling pathway. By inhibiting MEK1/2, binimetinib prevents the activation of ERK (extracellular signal-regulated kinase), thereby blocking downstream signaling that promotes cell proliferation and survival [5]. Although RAS mutations typically emerge as late events in AML pathogenesis, Binimetinib was included to investigate its potential to modulate RAS-MEK-ERK signaling in treatment-naive AML, where baseline expression levels of this pathway can influence sensitivity to conventional chemotherapeutic agents [6].

Pacritinib is an oral tyrosine kinase inhibitor that selectively targets JAK2 and FLT3 kinases [7]. By blocking JAK2's kinase activity, pacritinib suppresses the overactive JAK-STAT signaling that drives pathogenic cell proliferation and cytokine production in diseases such as myelofibrosis. It was selected for repurposing due to the dual inhibition of growth signaling pathways: the JAK2/STAT pathway, critical in hematopoietic cell growth and inflammatory signaling, and FLT3-driven proliferative signaling that regulates leukemic cell survival and also facilitates the development of escape pathways to targeted therapies [8].

Dimethyl fumarate (DMF) is an immunomodulatory drug that activates the Nrf2 (nuclear factor erythroid 2–related factor 2) pathway via covalent modification of the cysteine residues on Keap1, the cytosolic protein that normally binds Nrf2 and targets it for degradation. By oxidizing or alkylating the thiol groups of Keap1, DMF destabilizes the Keap1-Nrf2 complex, allowing Nrf2 to escape ubiquitination and translocate into the nucleus. In parallel, DMF also inhibits NFκB mediated transcription and was chosen for repurposing due to clinically relevant activity of NFκB in AML [9,10].

The statins (Cerivastatin and Pravastatin) were selected for their potential to induce metabolic reprogramming and modulate vesicular transport mechanisms in rapidly proliferating cells [11].

JNJ-64619178 (Onametastat) is a selective, potent and pseudo-irreversible inhibitor of protein arginine methyltransferase 5 (PRMT5), an enzyme that modulates the methylation program governing RNA splicing. This agent is hypothesized to demonstrate clinically meaningful activity in hematologic malignancies harboring splicing-factor mutations (notably SRSF2, U2AF1, SF3B1, and SF3A1), as well as in AML characterized by secondary-type mutational profiles [12,13].

Selinexor is a selective inhibitor of Exportin 1 (XPO1), a nuclear export protein responsible for transporting numerous cargo proteins including tumor suppressors (such as p53), and transcription factors (NFκB) from the nucleus to the cytoplasm. By inhibiting XPO1, selinexor forces nuclear retention of these proteins, leading to cell cycle arrest and apoptosis. For instance,

in NPM1-mutated AML, XPO1 inhibition prevents cytoplasmic mislocalization of mutant NPM1, enforcing its nuclear retention and driving terminal monocytic differentiation through downregulation of the MEIS1/HOXA9 leukemogenic program. More broadly, this mechanism represents a therapeutic vulnerability across hematologic malignancies and is currently being evaluated in solid tumors.

SNDX-5613 (Revumenib) is a selective oral inhibitor of the menin-KMT2A interaction. Menin serves as a critical oncogenic cofactor in acute leukemias with KMT2A rearrangements (occurring in up to 10% of AML) and NPM1 mutations (the most common genetic alteration in AML, occurring in up to 30% of cases). The menin-KMT2A complex drives aberrant expression of HOX genes (particularly HOXA9) and MEIS1, causing a hematopoietic differentiation block and leukemic transformation. Revumenib disrupts this interaction, downregulating these leukemogenic transcription factors and reversing the differentiation block. The drug has recently gained FDA approval for AML in the aforementioned contexts and is undergoing clinical investigation in combination regimens.

Palbociclib is an inhibitor of cyclin-dependent kinases 4 and 6 (CDK4/6), which are key regulators of cell cycle progression from G1 to S phase. By inhibiting CDK4/6, palbociclib prevents phosphorylation of the retinoblastoma protein (Rb), maintaining its tumor suppressor function and inducing G1 cell cycle arrest. While approved for breast cancer, CDK4/6 inhibitors are being repurposed for Kaposi's sarcoma, and also, being investigated in hematological malignancies for synergy with other targeted therapies to overcome resistance [14].

Venetoclax is a BCL-2 inhibitor that induces apoptosis in cancer cells by displacing pro-apoptotic proteins (such as BIM) from BCL-2, allowing them to activate the intrinsic apoptotic pathway. Venetoclax is already FDA-approved for AML in combination with hypomethylating agents or low-dose cytarabine for newly diagnosed elderly patients or those ineligible for intensive chemotherapy. This drug was included in our panel to evaluate its synergistic potential with other targeted agents, particularly given that venetoclax-based combinations have demonstrated high response rates.

Pinometostat is a small molecule inhibitor of DOT1L (disruptor of telomeric silencing 1-like), a histone H3 lysine 79 (H3K79) methyltransferase. DOT1L is recruited by MLL fusion proteins in KMT2A-rearranged leukemias, where it aberrantly methylates H3K79 and drives expression of leukemogenic genes including HOXA9 and MEIS1. By inhibiting DOT1L, pinometostat reduces H3K79 methylation and downregulates these oncogenic transcription factors.

JQ1 is a small molecule inhibitor of bromodomain and extraterminal domain (BET) proteins, particularly BRD4. BET proteins are epigenetic readers that recognize acetylated lysine residues on histones and regulate transcription of key oncogenes including MYC. In AML, BRD4 is

frequently overexpressed and drives expression of genes critical for leukemic cell survival and proliferation. JQ1 competitively binds to the acetyl-lysine recognition pocket of BET bromodomains, displacing them from chromatin and suppressing oncogene transcription.

Olaparib is a poly(ADP-ribose) polymerase (PARP) inhibitor that blocks DNA single-strand break repair, leading to accumulation of DNA double-strand breaks and synthetic lethality in cells with defective homologous recombination repair (such as those with BRCA mutations). While olaparib is approved for ovarian, breast, pancreatic, and prostate cancers with homologous recombination deficiency, emerging evidence indicates that baseline DNA repair defects may confer PARP inhibitor sensitivity, and that combining PARP inhibition with DNA-damaging chemotherapy can enhance synthetic lethality.

MSA2 (N-myristoyltransferase inhibitor) targets N-myristoyltransferases (NMT1 and NMT2), enzymes that catalyze the attachment of myristate to N-terminal glycine residues of proteins—a post-translational modification essential for membrane localization and function of numerous signaling proteins. In cancer cells, NMT activity is frequently elevated and supports multiple pro-survival signaling cascades simultaneously including SRC family kinases, ARF GTPases, and immune checkpoint regulators.

5.4 *In vitro* wet-lab validation setup

Single agent screening and IC_{50} determination. To evaluate the expert-selected drug candidates for acute myeloid leukemia (AML) repurposing, we determined their half-maximal inhibitory concentration (IC_{50}), the drug concentration required to inhibit cell viability by 50%, in representative cell lines (MOLM-13, KG-1a, HL-60, NOMO-1, and TK6). IC_{50} is a value commonly used as a way to quantify the effectiveness of a drug at inhibiting cellular processes, and can be measured by treating cells across a broad range of drug concentrations and fitting the dose response to a sigmoidal curve to determine 50% of maximal inhibition [15].

Cells were seeded in 96-well plates at a density of 5×10^3–1×10^4 cells per well and allowed to equilibrate overnight under standard culture conditions (37 °C, 5% CO_2, humidified incubator). Single-agent treatments were performed using an eight-point serial dilution series. Each compound was prepared as a concentrated stock and serially diluted (typically three to four-fold) to generate eight concentrations spanning the expected pharmacologically active range. Each dose was tested in triplicate together with vehicle-treated control wells. Plates were incubated for 48 to 96 hours before viability measurement.

Cell viability was assessed using the CellTiter 96® AQueous One Solution Cell Proliferation (MTS) Assay (Promega) following the manufacturer's instructions. Twenty microliters of reagent were added to each well and plates were incubated for 1 or 4 hours at 37 °C. Absorbance at 490 nm was recorded using a multimode microplate reader. Raw absorbance values were normalized to vehicle controls to compute percentage inhibition, which was plotted

against drug concentration to generate dose–response curves. Curve fitting and estimation of IC_{50} values were performed using GraphPad Prism.

Three cell lines used (MOLM-13, HL-60, and KG-1a) were generous gifts from the lab of Dr. Ravi Majeti (Stanford University), the NOMO-1 cell line was provided by Creative Bioarray, and the TK6 cell line was provided by ATCC.

- MOLM-13 cell line is a human AML cell line that was derived from bone marrow samples of a 62-year-old woman with AML.
- HL-60 cell line is a human cell line from a patient with acute promyelocytic leukemia (APL), often used to study the development and proliferation of normal and leukemic cells.
- KG-1a cell line is a human cell line isolated from the bone marrow aspirate of a 59-year-old, white male with erythroleukemia that evolved into AML.
- NOMO-1 cell line is a human AML cell line derived from the bone marrow of a 31-year-old woman with acute monocytic leukemia (AML FAB M5a) at second relapse. The cell line harbors the t(9;11)(p22;q23) chromosomal translocation resulting in the KMT2A-MLLT3 (MLL-AF9) fusion gene.
- TK6 cell line is a non-AML lymphoblastoid cell line used as a non-AML control for establishing a therapeutic window and assessing AML-specific selective cytotoxicity.

Fixed-ratio combination treatment and Chou-Talalay synergy analysis. To quantitatively assess pharmacological interactions between candidate compounds, combination treatments were performed using a fixed-ratio design, and synergy was evaluated according to the Chou-Talalay combination index (CI) method. Each drug was first tested alone to determine its dose–response curve and IC_{50} value.

For combination studies, the two drugs were mixed at a fixed molar ratio, and an eight-point dilution series was prepared to span concentrations above and below their respective IC_{50} values, ensuring that the midpoint of the series corresponded approximately to the IC_{50} range of each agent. An eight-point serial dilution series was prepared to cover a broad range of effects, and the mixtures were applied to cells in triplicate. After 48 to 72 hours of drug exposure, cell viability was determined using the CellTiter 96® AQueous One Solution MTS Assay (Promega), as described above. Absorbance readings at 490 nm were normalised to the vehicle control to calculate percentage inhibition at each concentration. Cell lines for combination treatment studies were from AddexBio (MOLM-13), and ATCC (KG-1a).

Synergy analysis was performed using the Chou-Talalay method, which quantitatively determines whether two drugs act synergistically, additively, or antagonistically based on their dose–response behavior. Dose-effect data from single-agent and combination treatments were analysed using Julius AI statistical software, which computes CI values for each dose–effect level. Interaction outcomes were classified as synergistic ($CI < 1$), additive ($CI \approx 1$), or antagonistic ($CI > 1$). Results were visualized as Fa-CI plots, showing the relationship between fractional effect (Fa) and CI, and as isobolograms at selected effect levels.

Three drug combination treatments. We tested three drugs together across a grid of doses in each cell line and measured the response. Raw readouts were converted to fractional effect relative to controls so everything was on the same 0 to 1 scale.

To judge whether the combinations performed better than expected, we used two standard checks:

- Bliss independence compares the observed combination effect to what you would expect if the drugs acted independently (no interaction).
- Highest Single Agent (HSA) compares the combination to the best single drug at the same doses.

For each dataset, we visualized interaction as dose–dose heatmaps with a color scale centered at zero (red = synergy, blue = antagonism, white = additivity). To compress the three-drug space into a single figure, we took each A × B dose pair and scanned across all C doses to find the strongest positive interaction (the "max over C"). We annotated each heatmap cell with the C dose that produced that maximum. Figures were generated in Python using pandas/numpy for data handling and seaborn/matplotlib for plotting.

5.5 Additional wet-lab results

This section provides additional *in vitro* laboratory results of drug repurposing. Extended Data Fig. 3 shows dose-response curves of Binimetinib in KG-1a, HL-60 and TK6 cell lines. Extended Data Fig. 4 shows the dose response curves of drug repurposing candidates for AML suggested by Co-Scientist that had little to no effect on MOLM-13 cells.

Comprehensive analysis of drug combination synergies. To systematically investigate the novel drug interactions proposed by Co-Scientist, we evaluated the full panel of combinations in MOLM-13 and KG-1a cell lines (Fig. 4 and Extended Figs. 5 and 6). In MOLM-13 cells, responses were predominantly synergistic. Strong and consistent synergy was observed for dual combinations including JNJ-64619178 + Selinexor, JNJ-64619178 + SNDX-5613, and Palbociclib + Selinexor, as well as the triple combinations of JQ1 + Olaparib + MSA2 and JNJ-64619178 + SNDX-5613 + Selinexor. In contrast, KG-1a cells exhibited highly context-dependent responses with a mixture of synergy and antagonism. While Palbociclib + Selinexor and Venetoclax + Pinometostat showed strong synergistic effects, the combination of JNJ-64619178 + Selinexor had antagonism at lower effect levels, transitioning to synergy at the highest effects. Notably, while the JNJ-64619178 + SNDX-5613 + Selinexor triplet was synergistic, the JQ1 + Olaparib + MSA2 combination was predominantly antagonistic in this cell line. A complete summary of these findings is tabulated in Extended Data Table 2.

These diverse interaction patterns heavily reflect the underlying molecular and cytogenetic profiles of the respective cell lines. Of note, KG-1a harbors mutant TP53, a complex karyotype with multiple chromosomal aberrations including del(7q) and various translocations,

and enrichment for $CD34^{+}CD38^{-}$ leukemic stem-like cells with inherent chemoresistance characteristics. Clinically, this cell-line represents a less differentiated, more resistant AML subtype that models the molecular heterogeneity with impaired apoptotic pathways and resistance to standard chemotherapeutic agents. The drug interaction and synergy patterns observed in KG-1a cells, including emergence of antagonism for several combinations, likely reflect the TP53-mutant phenotype, as TP53 mutations are associated with abysmal survival outcomes and poor response to standard-of-care chemotherapy in AML. The lack of Binimetinib response in NOMO-1 warrants further investigation, though we postulate this may reflect pathway redundancy and compensatory signaling mechanisms common in KMT2A-rearranged AML. Studies show that MEK inhibition in these cells often triggers sustained activation of parallel survival pathways [16], particularly PI3K/AKT/mTOR signaling, which enables cells to bypass MAPK pathway blockade. This is consistent with broader findings that MEK inhibitors demonstrate limited single-agent efficacy in KMT2A-rearranged AML and require combination with other targeted agents, such as menin inhibitors, to achieve meaningful anti-leukemic effects [17].

We evaluated single-agent efficacy for a broader panel of Co-Scientist proposed drugs against MOLM-13 and KG-1a AML cell lines. Cells were treated and incubated for 72 to 96 hours before viability measurement and IC_{50} determination. The full results of the single-agent efficacy are listed in Extended Data Table 1. Finally, the overall summary of the drug combination experiments of synergy is in Extended Data Table 2.

5.6 Clinical variable analysis guideline for AML drug repurposing

The structured framework below outlines six key evaluation domains for assessing repurposed drugs in AML treatment.

1. Patient-specific clinical and demographic variables: A patient's individual characteristics are crucial in determining their ability to tolerate different treatment regimens.
    - Age: A crucial prognostic indicator that significantly impacts treatment tolerance and survival outcomes. Treatment intensity should be calibrated across age groups: Younger patients (typically <60 years) are generally candidates for intensive chemotherapy regimens and multi-drug combinations (doublets or triplets). Patients aged 60-75 years require careful assessment for intensive versus non-intensive approaches based on individual fitness and comorbidity profile. Intensive chemotherapy should not be offered to patients over 75 years due to elevated treatment-related mortality risk. Non-intensive regimens or single-agent therapies are more appropriate alternatives. When evaluating a repurposed drug candidate, analyze any available safety data and comment on the specific age groups where the drug would be most beneficial. Explain your age-based

recommendations considering the drug's specific properties and how they align with the tolerability and treatment goals across different age groups.

- Performance status (e.g., ECOG score): Treatment selection should be stratified as follows: Patients with excellent to good performance status (ECOG 0-1) are optimal candidates for intensive therapies including combination regimens; patients with moderate performance status (ECOG 2) may receive intensive therapies with careful monitoring or moderate-intensity approaches; patients with poor performance status (ECOG 3) should receive less intensive single-agent therapies or supportive care measures; and patients with very poor performance status (ECOG 4) require palliative care approaches only. When evaluating a repurposed drug candidate, analyze any available safety data to determine the appropriate ECOG performance status range for safe administration. Consider the drug and explain which ECOG levels would be most suitable, particularly when evaluating combination regimens that require better functional status for tolerability.
- Comorbidities (e.g., Charlson Comorbidity Index): Concurrent medical conditions, quantified through validated tools like the Charlson Comorbidity Index (CCI), fundamentally influence treatment selection and prognosis. Treatment recommendations should be stratified by comorbidity burden: Patients with low CCI scores (0-2) can generally tolerate standard to intensive regimens; moderate CCI scores (3-4) warrant dose modifications or alternative approaches; and high CCI scores (≥ 5) typically require reduced-intensity or supportive care strategies. When evaluating repurposed drug candidates, comment on specific contraindications and dose adjustments required for major comorbidities including cardiovascular disease (heart failure, coronary artery disease), renal insufficiency, hepatic dysfunction, pulmonary disease, diabetes mellitus, and prior malignancies. Comment on how the drug's safety profile aligns with common AML patient comorbidities and specify any absolute contraindications or necessary monitoring requirements.
- Prior treatment history: Consider three main categories: treatment-naive patients (generally better response rates), patients with antecedent hematological disorders like myelodysplastic syndromes or myeloproliferative neoplasms who received prior treatments and developed therapy-related AML, and patients with prior chemotherapy or radiation for other malignancies who have cumulative toxicity constraints. When evaluating repurposed drug candidates, assess the drug's efficacy in treatment-naive versus relapsed/refractory populations, identify potential cross-resistance with prior therapies, evaluate cumulative dose limitations (particularly for agents like anthracyclines with lifetime dose caps), and consider contraindications based on germline predispositions or hereditary

cancer syndromes. Comment on whether the repurposed drug offers advantages in overcoming resistance mechanisms associated with specific prior treatments.

2. AML risk stratification based on molecular and cytogenetic profile (ELN 2022 and ELN 2024): AML risk stratification guides treatment decisions through molecular and cytogenetic profiling. The ELN 2022 guidelines stratify patients eligible for intensive chemotherapy, while ELN 2024 guidelines address older patients unfit for intensive treatment.
    - ELN 2022 risk categories (intensive treatment eligible)
        - Favorable-risk
            - t(8;21) RUNX1::RUNX1T1 (note: KIT mutations worsen prognosis)
            - inv(16)/t(16;16) CBFB::MYH11 (good initial response, relapse risk exists)
            - NPM1 mutation without FLT3-ITD
            - In-frame bZIP CEBPA mutations (biallelic)
        - Intermediate-risk
            - NPM1 mutation with FLT3-ITD
            - Wild-type NPM1 with FLT3-ITD
            - t(9;11) MLLT3::KMT2A
        - Adverse-risk
            - inv(3)/t(3;3) involving GATA2, MECOM(EVI1)
            - Complex karyotype (≥ 3 unrelated abnormalities)
            - Monosomal karyotype
            - Myelodysplasia-related gene mutations (ASXL1, BCOR, EZH2, RUNX1, SF3B1, SRSF2, STAG2, U2AF1, ZRSR2)
            - TP53 mutations (associated with therapy resistance and poor survival)
    - ELN 2024 risk categories (non-intensive treatment)
        - Favorable-risk: IDH1 mutations (TP53 wild-type), IDH2 mutations (FLT3-ITD negative, KRAS/NRAS wild-type, TP53 wild-type), NPM1 mutations (FLT3-ITD negative, KRAS/NRAS wild-type, TP53 wild-type), DDX41 mutations, other cytogenetic abnormalities if FLT3-ITD negative, KRAS/NRAS wild-type, TP53 wild-type
        - Adverse-risk: TP53 mutations
        - Intermediate-risk: All others not classified as favorable or adverse
    - When evaluating repurposed drug candidates, consider the drug's mechanism of action and existing efficacy data to comment on its potential effectiveness across these risk categories. Identify specific molecular targets the drug may address and explain whether the drug shows preferential activity against particular mutations or cytogenetic abnormalities. Consider if the drug could overcome resistance

mechanisms associated with adverse-risk features, particularly TP53 mutations, complex karyotypes, or myelodysplasia-related gene mutations. Consider how risk stratification should influence patient selection and dosing strategies for the repurposed drug.

3. Preclinical cell line testing: Effective preclinical evaluation requires strategic cell line selection to assess drug efficacy across AML's molecular diversity.
    - Cell line selection for testing. Select appropriate AML cell lines based on the drug's mechanism of action and target molecular features. Consider representative cell lines from major risk categories:
        - Favorable-risk models: **HL-60 (promyelocytic)**, Kasumi-1 (t(8;21)), ME-1 (inv(16))
        - Intermediate-risk models: **MOLM-13 (FLT3-ITD, NPM1 mutation)**, OCI-AML3 (NPM1 mutation)
        - Adverse-risk models: **KG-1a (TP53 mutation)**, SKM-1 (complex karyotype)
    - Basic efficacy assays
        - Cell viability assays (MTT, CellTiter-Glo): Determine IC50/EC50 values
        - Apoptosis assays (Annexin V/PI): Assess cell death mechanisms
        - Combination studies: Test synergy with standard AML drugs (cytarabine, anthracyclines, venetoclax)
    - Primary sample validation: Plan for *ex vivo* testing on primary AML samples with known molecular profiles to validate cell line findings.
    - When evaluating a repurposed drug candidate, recommend specific cell lines for testing based on the drug's mechanism of action and molecular targets. Justify cell line selections by explaining how their molecular characteristics (mutations, translocations, risk category) align with the drug's expected activity. Consider which assays would be most informative for demonstrating the drug's efficacy and whether specific molecular subtypes might show enhanced sensitivity.
4. Off-target effects and safety profile: Repurposed drugs have existing safety data that must be evaluated in the AML context to identify beneficial or harmful off-target effects.
    - Known safety profile analysis
        - Existing adverse events: Review the drug's known side effect profile from its original indication
        - Hematologic toxicity: Assess impact on normal blood cell production and bone marrow function
        - Organ-specific toxicity: Evaluate liver, kidney, cardiac, and neurologic effects
        - Drug interactions: Consider interactions with standard AML medications
    - Off-target effect evaluation

    - Beneficial off-targets: Identify secondary mechanisms that could enhance anti-leukemic activity
    - Harmful off-targets (anti-targets): Recognize interactions that could cause toxicity or interfere with treatment
    - Hematopoietic stem cell toxicity: Assess potential damage to the bone marrow and hematopoietic precursors
  - Safety monitoring requirements
    - Laboratory monitoring: Determine necessary blood tests and organ function assessments
    - Dose modifications: Consider if AML patients require different dosing than the original indication
    - Contraindications: Identify patient populations who should avoid the drug
  - When evaluating a repurposed drug candidate, analyze the drug's known safety profile from its original therapeutic use and comment on how these effects translate to AML patients. Identify potential off-target effects that could be beneficial (additional anti-cancer mechanisms) or harmful (anti-targets causing toxicity). Recommend safety monitoring strategies specific to AML treatment. Consider whether the drug's side effect profile is acceptable given AML's poor prognosis and existing treatment toxicities.
5. Pharmacokinetic and pharmacodynamic (PK/PD) variables: Repurposed drugs have established PK/PD data that must be evaluated for AML application to ensure effective and safe dosing.
  - Plasma concentration and dosing
    - Achievable concentrations: Compare effective in vitro concentrations (IC50/EC50) with known achievable plasma levels from the original indication
    - Dose requirements: Assess if standard dosing from the original indication provides adequate drug exposure for anti-leukemic activity
    - Formulation considerations: Evaluate if route of administration (oral vs. IV) is appropriate for AML patients
  - Drug metabolism and interactions
    - Metabolic pathways: Review primary elimination routes (hepatic, renal, other)
    - CYP interactions: Critical consideration given AML patients frequently receive antifungal medications (strong CYP inhibitors like posaconazole, voriconazole)
    - Supportive care interactions: Assess interactions with antibiotics, antiemetics, and other supportive medications
  - Response monitoring

    - Target engagement biomarkers: Identify measurable indicators that the drug is affecting its intended target
    - Efficacy biomarkers: Determine laboratory or molecular markers to assess treatment response
    - Safety biomarkers: Establish monitoring parameters for early toxicity detection
- When evaluating a repurposed drug candidate, consider whether the drug's established dosing regimen can achieve effective anti-leukemic concentrations based on in vitro data. Comment on potential drug interactions, particularly with antifungal medications commonly used in AML patients. Identify existing biomarkers that could monitor drug activity and treatment response, or propose new biomarkers if none exist.

Case Study: Application of the Clinical Variable Framework to Binimetinib. To demonstrate the utility and instruction-following capability of the structured evaluation framework detailed above, we present Co-Scientist's translational analysis for the repurposed candidate, Binimetinib.

When applied to Binimetinib, this analysis yielded a specific unmet-need clinical context: Older or frail patients with relapsed or venetoclax-resistant AML (aligning with Point 1: Patient-specific clinical and demographic variables).

Specifically addressing the pharmacokinetic parameters and drug interactions (Point 5), Co-Scientist identified Binimetinib's UGT1A1-mediated metabolism as a meaningful advantage, noting that it circumvents the CYP3A4-dependent drug-drug interactions that commonly limit therapeutic options in patients receiving azole antifungals. It then linked this favorable pharmacokinetic profile to a well-recognized challenge in managing venetoclax-resistant disease, where MAPK-pathway activation and polypharmacy frequently constrain combination therapy. This synthesis of pharmacokinetic, molecular, and clinical considerations supported the conclusion that Binimetinib could be particularly well suited for frail or heavily pretreated patients, especially in contexts where CYP3A4 inhibition limits the feasibility of other targeted regimens.

6 Detailed Co-Scientist output for a validated AML repurposing candidate

KIRA6 for AML

Summary
This proposal outlines repurposing KIRA6, an IRE1α inhibitor, for treating acute myeloid leukemia (AML), particularly FLT3-ITD positive AML known for high relapse rates. The hypothesis is that KIRA6 will disrupt protein homeostasis and induce ER stress in AML cells, synergizing with existing therapies and overcoming resistance. The proposal details KIRA6's mechanism, impacting pathways like the UPR and apoptosis, and suggests a comprehensive experimental plan including in vitro and in vivo studies to validate its efficacy and safety, including combination therapy with existing AML drugs. This approach aims to provide a novel, effective treatment strategy for AML patients, particularly those with the challenging FLT3-ITD mutation and other drug resistance phenotypes.

Hypothesis

Introduction
Acute myeloid leukemia (AML) remains a formidable hematological malignancy characterized by the rapid proliferation of immature myeloid blasts in the bone marrow and peripheral blood. Despite advancements in therapeutic strategies, including targeted therapies and hematopoietic stem cell transplantation, a significant proportion of patients experience relapse or develop resistance to treatment, underscoring the urgent need for new therapeutic interventions. The heterogeneous nature of AML, with diverse genetic and molecular aberrations driving disease progression, necessitates the exploration of novel therapeutic targets and the rational design of combination therapies to overcome treatment resistance and improve long-term survival outcomes. Drug repurposing, which involves identifying new uses for existing drugs, offers a cost-effective and efficient strategy to accelerate the development of novel AML treatments by leveraging established safety profiles and pharmacokinetic data. This approach can significantly reduce development time and costs, facilitating the rapid translation of promising agents to clinical trials.

Recent findings and related research
Recent research has significantly advanced our understanding of the complex molecular landscape of AML, highlighting the critical roles of various genetic mutations and signaling pathways in leukemogenesis. Recurrent mutations in genes such as FLT3, NPM1, IDH1/2, and TP53 are frequently observed in AML and have been implicated in disease initiation, progression, and treatment resistance. The FLT3-ITD mutation, a particularly challenging subtype, is associated with poor prognosis and a higher risk of relapse, emphasizing the need for effective therapies that target this specific mutation or overcome its downstream effects. Targeted

therapies, such as FLT3 inhibitors (midostaurin, gilteritinib), IDH inhibitors (enasidenib, ivosidenib), and the BCL-2 inhibitor venetoclax, have demonstrated clinical efficacy in specific AML subtypes; however, the emergence of drug resistance and the lack of effective treatments for high-risk patients remain critical challenges.

Drug repurposing has yielded several promising candidates for AML treatment, including:

- Arsenic trioxide and all-trans retinoic acid (ATRA): Established treatments for acute promyelocytic leukemia (APL), these have also shown potential in combination therapy for non-APL AML.
- Histone deacetylase (HDAC) inhibitors: Vorinostat and panobinostat have shown modest activity in AML, particularly in combination with other agents, but are known to be poorly tolerated and have limited efficacy as single agents.
- Proteasome inhibitors: Bortezomib and carfilzomib have been explored in combination regimens in AML, especially to overcome resistance, though with limited success.
- Metabolic inhibitors: Targeting glutamine metabolism and other metabolic pathways has shown promise in preclinical studies, though with limited translation due to toxicity and poor absorption.
- Homoharringtonine (HHT): As noted, HHT, a protein synthesis inhibitor, has been approved for CML and has shown promise in AML, although its mechanism of action and specific impacts on resistance mechanisms need to be further explored.
- Immunomodulatory drugs: Thalidomide and lenalidomide have been explored in combination therapies to target the tumor microenvironment and enhance immune responses, though with limited efficacy in AML.
- Kinase inhibitors: Beyond FLT3, new kinase inhibitors that target other pathways are being explored in combination settings to overcome resistance.

Despite these findings, there remains a significant unmet need for novel, effective therapies that can overcome drug resistance, target novel pathways, and improve long-term survival for AML patients.

Areas worth exploring

Several areas hold significant potential for identifying novel or repurposed drugs for AML treatment:

- Targeting non-canonical signaling pathways: Explore drugs that target pathways beyond the well-established ones, such as those involved in inflammatory signaling, protein degradation, and DNA damage repair. The interplay between the AML cell and its microenvironment, including inflammatory cytokines and immune cells, remains an area of active investigation and a promising target for therapeutic intervention.
- Targeting RNA processing and translation: Aberrant RNA processing and translation are significant drivers of protein expression in cancer, and are potential targets for novel

therapies. Targeting ribosome function, mRNA splicing, and RNA modifications can disrupt the production of key proteins required for cell survival and proliferation.

- Modulating immunometabolism: Cancer cells exhibit altered metabolic demands that influence the immune response, and targeting metabolic pathways that influence immune activation and suppression could enhance anti-leukemic immune responses. This also includes the metabolic dependencies of the myeloid microenvironment, which is critical for disease progression and resistance.
- Leveraging high-throughput screening (HTS) and computational biology: Combine in vitro or ex vivo screening of approved drugs with computational drug repurposing methods, particularly those that integrate multi-omics data, to identify novel drug candidates and potential synergistic drug combinations.
- Focus on drug combinations: Rational drug combinations that address both active malignancy and underlying resistance mechanisms, and can achieve effective treatment regimens at lower concentrations in combination, will have better safety, efficacy and overcome existing resistance. This should include a focus on sequential drug administration and drug scheduling.
- RNA-based therapies: Direct targeting of mRNAs coding for leukemia-driving proteins can be a very impactful strategy that also offers the chance to overcome resistance, for example by interfering with compensatory pathways. This includes RNA interference (RNAi), antisense oligonucleotides (ASOs), and mRNA-based therapies.
- Targeting protein homeostasis: Disrupting the protein homeostasis network, including protein folding, degradation (through ubiquitin-proteasome and autophagy pathways), and quality control mechanisms, can induce cellular stress and apoptosis in AML cells.

Detailed novel likely correct idea: repurposing the IRE1α inhibitor KIRA6 for AML treatment

Goal: Repurpose KIRA6, an IRE1α inhibitor, for AML treatment, specifically targeting the MOLM-13 cell line with FLT3-ITD, with a focus on overcoming resistance mechanisms and enhancing combination therapy efficacy.

Idea: KIRA6, by inhibiting IRE1α, a key regulator of the unfolded protein response (UPR), can disrupt protein homeostasis and induce ER stress in AML cells, particularly in cells exhibiting high metabolic demand and increased protein synthesis, such as FLT3-ITD positive cells. This disruption can synergize with other therapies, including FLT3 inhibitors and chemotherapeutics, to overcome resistance and improve therapeutic efficacy. KIRA6 will also suppress chronic inflammatory pathways known to promote cell survival in AML cells.

Molecular mechanism of action

- Inhibition of IRE1α: KIRA6 is a selective inhibitor of IRE1α, a transmembrane protein in the endoplasmic reticulum (ER) that acts as a key sensor and transducer of ER stress. IRE1α activation initiates the unfolded protein response (UPR) pathway, which is a cellular stress response aimed at restoring protein homeostasis in the ER. Under conditions of increased protein synthesis or misfolded proteins, IRE1α is activated, leading to the splicing of XBP1 mRNA and the subsequent activation of downstream transcriptional targets involved in protein folding, trafficking, and degradation.
- Disruption of ER homeostasis: By inhibiting IRE1α, KIRA6 blocks the adaptive arm of the UPR, preventing the resolution of ER stress and leading to the accumulation of unfolded and misfolded proteins. This disruption creates a protein folding crisis, inducing ER stress and initiating apoptotic pathways. This effect is potentiated in rapidly proliferating AML cells, which have higher metabolic demands and are more sensitive to ER stress.
- Downstream effects:
    - The accumulation of misfolded proteins leads to the activation of the PERK and ATF6 arms of the UPR, which further contribute to ER stress and apoptosis.
    - Inhibition of IRE1α disrupts the transcriptional program regulated by XBP1, impairing the production of proteins involved in ER homeostasis and cell survival.
    - The resulting ER stress induces the activation of the integrated stress response (ISR), leading to the translational suppression of many transcripts and increased apoptotic activity.
    - KIRA6 can indirectly inhibit the NF-κB pathway by reducing ER stress and inflammatory cytokine production, reducing cell survival, proliferation and resistance.
    - KIRA6 may directly reduce levels of inflammatory cytokines such as IL-1, in turn reducing secondary activation of inflammatory signalling cascades and reduce activation of IRAK1 in the process.
- Impact on FLT3-ITD: FLT3-ITD mutations lead to increased cell proliferation and metabolic demand and stress. KIRA6 can target this through disruption of the UPR, leading to a higher impact on FLT3-ITD cells. FLT3-ITD cells are under high levels of stress already, and require high levels of protein synthesis to maintain viability. By targeting basal or activated IRE1α, KIRA6 can induce significantly more cell death in FLT3-ITD cells than their wild-type counterparts.
- Impact on key dysregulated pathways:
    - MYC: MYC protein levels are directly tied to mRNA translation and are necessary for cell survival and resistance in many cases, including leukemia. Targeting the UPR and downstream translation with KIRA6 directly decreases MYC expression and survival. This also has downstream anti-inflammatory benefits.

    - NF-κB signaling: KIRA6 can reduce NF-κB activity, a key driver of cell survival, proliferation, and resistance, by reducing ER stress and inflammatory cytokine production.
    - MCL-1 and other anti-apoptotic proteins: KIRA6 will reduce the production of short-lived survival proteins, leading to rapid apoptosis by diminishing their production, particularly MCL-1 and other similar proteins, which are involved in anti-apoptotic effects in AML cells, and are known drug resistant mechanisms.
    - Targeting multiple AML resistance mechanisms: KIRA6, by disrupting protein homeostasis, has the capacity to overcome resistance across a wide panel of mechanisms, including through reduced efflux pump protein levels, and reduced repair mechanisms.
- Synergistic effects: KIRA6 can synergize with FLT3 inhibitors by increasing ER stress, making cells more sensitive to FLT3 inhibition. It can also enhance the activity of chemotherapeutic drugs by disrupting DNA repair pathways.

Impacted pathways

- Unfolded protein response (UPR): Directly impacted through inhibition of IRE1α.
- Apoptosis: Primarily through the intrinsic pathways, and disruption of anti-apoptotic proteins, and an increase in ER stress, alongside an increase of pro-apoptotic proteins through the integrated stress response.
- Cell cycle: Perturbation of cell cycle progression through reduced levels of cell-cycle proteins.
- NF-κB signaling: KIRA6 can reduce NF-κB activity which reduces inflammation and promotes cell survival, and drug resistance.
- MYC-related pathways: Suppression of MYC and other related oncogene protein levels.
- Inflammatory pathways: By suppressing cytokines, most notably IL-1, KIRA6 can suppress activation of IRAK1 and reduce chronic inflammation that promotes cell survival in addition to killing cancer cells.

Effect on AML (MOLM-13) cell proliferation inhibition

- KIRA6 selectively inhibits proliferation of MOLM-13 cells, and also AML cells with other mutations that render them drug resistant.
- IRE1α inhibition leads to the activation of apoptotic pathways and reduced cell proliferation and clonal survival.
- Suppression of MYC levels, NF-κB, MCL-1 and other cell survival pathways will reduce cell survival and promote apoptosis, which should be enhanced in FLT3-ITD AML due to their high proliferation rates and increased sensitivity to metabolic/translational stress in comparison to normal myeloid cells.
- In addition to having a direct effect on proliferation, a reduction in inflammatory signalling (IL-1 and IRAK1) can potentiate activity of other drugs.

- KIRA6 treatment results in a dose-dependent reduction in MOLM-13 cell viability.
- KIRA6 leads to a buildup of ER stress and activation of the ISR, all which enhance its effectiveness as a targeted drug.
- When combined with FLT3 inhibitors or chemotherapeutic drugs, KIRA6 is expected to show additional impacts at lower overall drug concentrations (lower levels of both KIRA6 or the alternative drugs in the combination will be required for the same levels of cell death, or greater cell death will be seen at the standard doses used presently in the clinic). This is very important given the high levels of both drug toxicity that are associated with all AML treatments, and the need to reduce those toxicities.

IC50 assay concentrations

Based on published data for KIRA6 in other cancer cell models and its known mechanism of action, a reasonable starting concentration range for an IC50 assay in MOLM-13 cells would be 100 nM to 10 µM.

- Rationale: This range spans the concentrations that have shown activity in various cell types while being within a pharmacologically achievable range. Prior in vitro studies have shown activity in low micromolar concentrations.
- Specific concentrations: 100 nM, 250 nM, 500 nM, 1 µM, 2.5 µM, 5 µM, 7.5 µM, 10 µM.
- Assay method: Cell viability assays (e.g., MTT, CellTiter-Glo) to determine IC50 values after KIRA6 exposure for 48-72 hours.
- Controls: Appropriate vehicle controls (DMSO) or medium-only controls should be included.
- Positive control: Use a known FLT3 inhibitor, such as gilteritinib, at its reported IC50 in MOLM-13 cells for normalization of assay variability and comparison.
- Combination studies: The combination of a FLT3 inhibitor at a single set concentration, plus a matrix of KIRA6 concentrations at several ratios will be evaluated using the viability assays as a baseline. Additional confirmation using apoptotic assays and cell cycle arrest should be done for any combination that shows better results than single-agent drug effects.

Safety and toxicity

- Limited safety data: KIRA6 is a relatively new compound and has limited safety data in humans, as it has not gone through clinical trials. Initial in vitro and in vivo studies in other disease settings (mostly cancer) suggest that it is generally well-tolerated. However, thorough preclinical toxicity studies are essential before advancing to human trials.
- Potential toxicity: Given the mechanism of action, potential toxicities include ER stress-related effects on normal tissues, particularly those with high protein synthesis demands (e.g., liver, pancreas). However, since AML cells are under high levels of stress,

and have high metabolic demand, they are expected to be disproportionately impacted by this drug, which should reduce systemic toxicity.

- Repurposing advantage: Because KIRA6 has established in vitro activity, and has known pharmacology, it is significantly less risky than a compound that would have to have all of its safety profiles evaluated before human trials.
- Combination therapy impact: Because KIRA6 can be paired with existing therapies, it may be possible to reduce the overall dose of both agents to minimize side-effects and overall toxicity, while enhancing therapeutic efficacy. This should be tested rigorously in vitro through all of the combination studies before any movement into clinical human or animal trials to make sure that the benefit is higher than the risk.

Testable hypothesis

KIRA6, by inhibiting IRE1α, will selectively inhibit the proliferation of AML cells, especially FLT3-ITD positive cells, by disrupting protein homeostasis and inducing ER stress. The increase in stress and reduced output of essential cell maintenance proteins will trigger apoptotic pathways, leading to cell death, particularly in highly proliferative settings. KIRA6 will potentiate the activity of existing AML therapies such as FLT3 inhibitors and chemotherapeutics. The drug will also have a broader impact on resistance mechanisms.

Experimental plan

- IC50 determination in MOLM-13: Determine IC50 values in MOLM-13 cells using viability assays with KIRA6 concentrations ranging from 100 nM to 10 µM.
- ER stress marker measurement: Measure levels of key ER stress markers (e.g., BiP/GRP78, CHOP) using Western blotting and ELISA after KIRA6 treatment to confirm the impact on ER homeostasis.
- IRE1α activity: Measure XBP1 splicing using RT-PCR or other assays to confirm KIRA6's impact on IRE1α activity.
- Apoptosis assays: Evaluate apoptosis by Annexin V/PI staining, caspase activity assays, and Western blot analysis of apoptotic markers (cleaved PARP, cleaved caspase-3 levels) in KIRA6-treated MOLM-13 cells.
- Western blots: Analyze key pathway proteins: including MYC, NF-κB, MCL-1, FLT3, AKT/mTOR, and key components of the canonical inflammatory pathways (including IL-1, IL-6, TNF-alpha, IRAK1/4, MyD88), and markers of endoplasmic reticulum (ER) stress in AML cells treated with KIRA6.
- ISR markers measurement: Evaluate the induction of the Integrated Stress Response using assays measuring levels of phospho-eIF2α, ATF4, and CHOP.
- Combination studies: Evaluate the combination of KIRA6 with FLT3 inhibitors like gilteritinib, with chemotherapeutics (cytarabine, daunorubicin) or with venetoclax in MOLM-13 cells using combination matrix studies. This will establish if there is synergistic activity and enable evaluation of lower doses of each drug. Repeat these on

additional cell lines with and without FLT3 mutations to confirm consistent synergistic enhancement.

- Cell cycle analysis: Measure cell cycle arrest using DNA staining (propidium iodide) by flow cytometry.
- RNA stress markers studies: Evaluate markers of RNA stress to assess damaged mRNA levels and ER Stress markers to determine cellular stress induced by the drug.
- Efflux pump activity: Measure the activity of key drug efflux pumps (e.g., ABCB1, ABCG2) using specific inhibitors and substrates to evaluate KIRA6's impact on drug resistance mechanisms.
- Expansion to other cell lines: Repeat the above assays on multiple AML cell lines, with both FLT3-ITD and wild-type cells, and lines representing other AML mutations or drug resistance phenotypes. Also evaluate primary AML patient cells in co-culture with stroma and alone with comparable controls, including primary non-leukemic bone marrow derived cells for measuring minimal activity in normal myeloid cells. This should allow for verification of the generalizability of KIRA6.
- In vivo xenograft studies: Evaluate the efficacy and safety of KIRA6 in mouse xenograft models using MOLM-13 cells or other cell lines to assess its overall treatment value and toxicities.
- Pharmacokinetics studies: Evaluate absorption, distribution, metabolism, and excretion of KIRA6 in animals to enable rational dose selection in future trials.

Conclusion

Repurposing KIRA6, an IRE1α inhibitor, for AML treatment, with a focus on FLT3-ITD positive cells and overcoming resistance mechanisms, represents a promising novel approach. KIRA6's ability to disrupt protein homeostasis, induce ER stress, synergize with existing AML drugs, and potentially overcome resistance mechanisms makes it an excellent drug candidate for testing. The comprehensive experimental plan proposed here offers a thorough framework for validating the potential of KIRA6 in AML and allows for a better way to select drugs for clinical testing by first performing a rigorous scientific evaluation in vitro using a multi-faceted approach. KIRA6 could translate into a new and effective strategy for AML patients, particularly those with the challenging FLT3-ITD mutation and other resistance phenotypes. Due to its mode of action on the UPR, it also has the potential to target underlying resistance mechanisms to existing therapies.

The focus on resistance mechanisms, a more diverse set of cell lines and primary cells, a well-rationalized combination approach, and a specific mechanism of action, all address the feedback from the previous version and provide a strong and detailed rationale for this drug repurposing strategy. The expanded experimental plan provides a thorough framework for assessing the efficacy, selectivity, and safety of KIRA6 in AML.

Review

Relevant article abstracts

1: Discusses targeting the IRE1α-XBP1s pathway in AML, directly relevant to the proposed mechanism of KIRA6.
5: Reviews the UPR as a therapeutic target in acute leukemias, providing context and supporting the rationale for targeting IRE1α.
6: Focuses on selective degradation of FLT3-ITD, offering insights into targeting specific forms of the mutated protein, aligning with the idea's focus on targeting ER-bound FLT3-ITD.
7: Offers the background on known pharmacology and toxicological studies for IC50 determination of drug candidates in tumor cells.
9: Discusses sensitizing AML to venetoclax treatment, highlighting the potential for combination therapy approaches, relevant to the idea's suggested synergistic effects with other AML drugs.

Assumptions

- KIRA6 effectively inhibits IRE1α in AML cells, particularly MOLM-13 cells.
- IRE1α inhibition leads to significant disruption of protein homeostasis and induction of ER stress in AML cells.
- The increased ER stress induced by KIRA6 selectively targets AML cells over healthy cells.
- KIRA6 synergizes with FLT3 inhibitors and chemotherapeutic agents to enhance AML cell death.
- The proposed IC50 concentration range (100 nM to 10 μM) is appropriate for testing in MOLM-13 cells.
- KIRA6 has a manageable safety profile and tolerable toxicity in preclinical models.
- KIRA6 can inhibit the production of short-lived proteins, like MYC and MCL-1, and reduce inflammatory signaling.

Reasoning about assumptions

- Plausible: [1] and [5] support the notion of targeting IRE1α in AML. While no direct information shows if KIRA6 effective inhibits IRE1α in AML MOLM 3 cells, it likely works as an IRE1α inhibitor like in other cell types. Needs experimental validation.
- Plausible: This is a core function of the UPR pathway in which IRE1α inhibition, based on the literature [5], should induce this cascade of events causing stress response leading to cell death if prolonged and severe.
- Plausible, but requires careful investigation: AML cells, particularly those with FLT3-ITD, have higher protein synthesis rates and are inherently more sensitive to ER stress. While this is not demonstrated in the literature, the rationale behind targeting ER stress response in leukemia is that tumor cells, particularly with FLT3-ITD, are expected

to be more reliant on these processes and therefore may be more susceptible, but this assumption needs further evaluation.

- Plausible, but requires testing: The rationale for synergy is based on the proposed mechanism and general principles of targeting multiple pathways in cancer. [9] offers a related example of synergy with venetoclax, a drug also being tested in FLT3-ITD AML. This hypothesis needs further in vitro testing to be demonstrated.
- Plausible: The suggested range is based on literature and seems reasonable as a starting point. This needs to be empirically confirmed in an IC-50 assay. The provided range in the idea is a well justified assumption that can be verified during the IC-50 viability assays where a broader range of concentrations would capture the optimal values.
- Unknown: This is a vital assumption requiring thorough preclinical testing. [7] has information about pharmacology and toxicity, which provides context. Currently, limited safety data exists for KIRA6 so the drug safety profile is unknown and needs experiments to verify if the drug is safe enough.
- Plausible: IRE1α inhibition and ER stress induction can trigger downstream effects that impact protein translation and inflammatory responses based on the mechanism described in the UPR [5]. [1] suggests that IRE1α impacts venetoclax efficacy. While this is not demonstrated in the literature for KIRA6 in AML, this remains an experimentally verifiable assumption.

Improvements to the idea

- Broader cell line testing: The idea primarily focuses on MOLM-13. Expanding to other AML cell lines, including those with different FLT3 mutations and other genetic backgrounds, would strengthen the proposal. This would require additional experiments as well.
- Deeper exploration of resistance mechanisms: While mentioned, the idea could benefit from a more detailed analysis of how KIRA6 addresses specific resistance mechanisms in AML. The idea can also benefit from additional experiments regarding resistance mechanism impacts.
- Investigate drug resistance: There is minimal data regarding KIRA6 impacting drug efflux pumps and other drug resistance mechanisms. It would be beneficial to investigate how impacting the UPR could impact drug resistant cells, which are a major reason for treatment failure in AML.
- Detailed comparison to other IRE1α inhibitors: If there are other IRE1α inhibitors, comparing and contrasting KIRA6 with them and justifying the reasons for choosing this particular drug would add to the idea's strength.

Reasoning about correctness and testing

- The idea is plausible and well-reasoned based on known mechanisms of IRE1α and the UPR in cancer. However, key assumptions about selectivity and synergy require

experimental validation. The proposed experimental plan is comprehensive and addresses the key questions.

- The idea is novel in its application of KIRA6 to AML, particularly in the context of FLT3-ITD. The focus on combination therapy and overcoming resistance is also promising.
- It is recommended to test this idea due to its plausibility, novelty, and potential clinical significance. While there are uncertainties regarding safety and efficacy, the potential benefits warrant further investigation.
- **Answer: 4**

Novelty review

- Targeting the IRE1α-XBP1s pathway to enhance venetoclax effectiveness in AML [1]: This abstract directly relates to the idea by exploring the IRE1α pathway in AML, although in combination with venetoclax, not as a monotherapy or in combination with FLT3 inhibitors.
- The unfolded protein response: A novel therapeutic target in acute leukemias [5]: This review discusses the UPR as a target in acute leukemias, providing a broader context for the idea's focus on IRE1α.
- Selective degradation of mutant FMS-like tyrosine kinase-3 requires BIM-dependent depletion of heat shock proteins [6]: This abstract explores the mechanisms of FLT3 degradation and its connection to BIM and heat shock proteins, relevant to the idea's focus on FLT3-ITD positive AML.

Already explored aspects

- Targeting the UPR in AML: The idea of targeting the unfolded protein response (UPR) in AML is not entirely novel. Abstract [5] discusses the UPR as a therapeutic target in acute leukemias, including AML. Abstract [1] specifically explores targeting IRE1α in combination with venetoclax, suggesting existing interest in this pathway for AML. However, the use of KIRA6 as a single agent or in combination with FLT3 inhibitors appears less explored.
- FLT3 inhibitors in AML: Targeting FLT3 in AML with tyrosine kinase inhibitors is a well-established approach, with multiple approved drugs. Abstracts [2, 3] focus on FLT3 inhibitors, highlighting the importance of this target but also the challenges of resistance [6].

Novel aspects

- Repurposing KIRA6 for AML: While IRE1α has been targeted in AML [1], the repurposing of KIRA6 specifically for AML treatment, especially in the context of FLT3-ITD positive disease and potential combination with FLT3 inhibitors or standard chemotherapies, seems novel based on the provided abstracts. The idea explores KIRA6's

impact on multiple pathways (UPR, apoptosis, NF-κB, MYC) related to AML proliferation and resistance.

- Focusing on FLT3-ITD positive AML & combination therapy: Although FLT3 inhibitors are used in AML, the idea of combining KIRA6 with FLT3 inhibitors or chemotherapeutics to specifically target FLT3-ITD positive AML cells and potentially overcome resistance addresses a current clinical need. The proposed mechanism – disrupting protein homeostasis in already stressed FLT3-ITD cells – offers a rationale for this combination approach.
- Combination of KIRA6 with other AML drugs & mechanism of action: KIRA6 is not mentioned in any of the abstracts, and therefore the proposed combination of KIRA6 and FLT3i to induce apoptosis is also novel. The described mechanism has strong logical support, as the cells' increased translation requirements and ER stress from the FLT3i mutation could greatly increase the effectiveness of IRAK1 inhibition, which may itself directly induce apoptosis, and also improve efficacy of existing drugs.

Novelty review

The idea presents a moderate level of novelty. Repurposing KIRA6 for AML, particularly in the context of FLT3-ITD positive disease and combination therapy, is a promising approach. However, targeting IRE1α in AML has been explored [1], albeit not with this specific drug and with different existing treatment approaches. The idea's strength lies in its detailed mechanistic rationale, combination therapy focus, and defined experimental plan. It is crucial to validate the proposed mechanism and selectivity across different AML cell lines and patient samples before concluding its true novelty.

Improvements to the idea

- Literature search: Conduct a thorough literature search beyond the provided abstracts to confirm the novelty of KIRA6 in AML. Investigate any existing research on KIRA6 in other hematological malignancies.
- Selectivity testing: Expand selectivity testing to include other healthy cell types beyond normal myeloid cells, to address potential off-target effects and demonstrate greater safety margin.
- Combination exploration: Further explore rational combinations beyond FLT3 inhibitors, including chemotherapeutic agents and other targeted therapies.
- Resistance mechanisms: Thoroughly investigate the impact of KIRA6 on various AML resistance mechanisms through in vitro testing, using resistant cell lines and patient-derived samples.
- In vivo efficacy and toxicity profile: Expand the in vivo studies with multiple cell lines and patient-derived xenograft models, with a focus on establishing the efficacy and toxicity profile of KIRA6, both as single-agent and in the proposed combination strategy.

Reasoning about novelty and recommendation
The idea is novel enough to warrant further exploration. While targeting IRE1α isn't completely new, using KIRA6 in AML, especially in the context outlined, hasn't been extensively investigated, and it also has not been previously examined as a companion drug with other therapies used in AML. The proposed combination with FLT3 inhibitors and the detailed rationale provide a strong foundation. The comprehensive experimental plan should be executed to confirm the preliminary findings and assess the true potential of KIRA6 in AML. If the in vitro and in vivo findings are positive, the idea would be worthy of publication in a specialized journal focused on hematological malignancies or drug repurposing.
Answer: 3

[1] Targeting the IRE1α-XBP1s Pathway to Enhance Venetoclax Effectiveness in AML | Blood | American Society of Hematology (https://ashpublications.org/blood/article/144/Supplement%201/4144/533082/Targeting-the-IRE1-XBP1s-Pathway-toEnhance)
[2] FLT3 inhibitors in acute myeloid leukemia: ten frequently asked questions | Leukemia (https://www.nature.com/articles/s41375-019-0694-3)
[3] Inhibition of FLT3-ITD Kinase in Acute Myeloid Leukemia by New Imidazo[1,2-b]pyridazine Derivatives Identified by Scaffold Hopping | Journal of Medicinal Chemistry (https://pubs.acs.org/doi/10.1021/acs.jmedchem.3c00575)
[4] Repurposing approved drugs on the pathway to novel therapies - PMC (https://pmc.ncbi.nlm.nih.gov/articles/PMC7018532/)
[5] The Unfolded Protein Response: A Novel Therapeutic Target in Acute Leukemias - PMC (https://pmc.ncbi.nlm.nih.gov/articles/PMC7072709/)
[6] Drugging the unfolded protein response in acute leukemias | Journal of Hematology & Oncology | Full Text (https://jhoonline.biomedcentral.com/articles/10.1186/s13045-015-0184-7)
[7] Selective degradation of mutant FMS-like tyrosine kinase-3 requires BIM-dependent depletion of heat shock proteins | Leukemia (https://www.nature.com/articles/s41375-024-02405-5)
[8] Pharmacology and Toxicology Testing: IC50 for Tumor Cell Lines - Altogen Labs (https://altogenlabs.com/pre-clinicalresearch-services/pharmacology-and-toxicology-testing-ic50-for-tumor-cell-lines/)
[9] Targeting Mitochondrial Structure Sensitizes Acute Myeloid Leukemia to Venetoclax Treatment | Cancer Discovery | American Association for Cancer Research (https://aacrjournals.org/cancerdiscovery/article/9/7/890/41983/TargetingMitochondrial-Structure-Sensitizes-Acute)

Critiques

Here's a summary of the negative critiques from the reviews:

- Limited safety data: The biggest concern is the lack of human safety data for KIRA6, requiring thorough preclinical toxicity studies. The drug's safety profile is currently unknown and needs experimental validation.
- Limited scope of cell line testing: Testing primarily focuses on MOLM-13. Expanding to other AML cell lines (with different FLT3 mutations and other genetic backgrounds) and primary patient samples is necessary.
- Limited comparison to other drugs: The idea would benefit from comparing KIRA6 to other IRE1α inhibitors or similar drugs to justify its selection and benchmark efficacy/selectivity. Minimal data exists regarding KIRA6 compared to other drugs.
- Lack of pre-clinical and clinical data: While proposed, in vivo and toxicity data are currently lacking for KIRA6, despite the comprehensive experimental plan.
- Assumptions requiring validation: Several assumptions about KIRA6's selectivity for AML cells over healthy cells, synergy with other drugs, impact on drug resistance mechanisms, and the suggested IC50 concentration range need experimental validation.
- Limited detail in some areas: The rationale for the specific IC50 range could be stronger. The exploration of resistance mechanisms and the details of synergy studies could also be more in-depth. More detail on how KIRA6 impacts MYC, NF-κB and inflammatory pathways is needed. The connection between UPR activation and a lower incidence of FLT3 mutations needs further investigation.
- Potential for resistance: While the idea aims to overcome resistance, there's still a need to investigate how KIRA6 itself might contribute to new resistance mechanisms over time.

Although the idea is considered plausible, novel, and promising, these weaknesses need to be addressed through further research and experimentation.

7 Safety and ethical implications

While AI systems such as Co-Scientist offer the potential to accelerate scientific discovery, it also poses significant safety and ethical challenges, distinct from its impact on the scientific method itself. Safety risks center on the dual-use and the possibility that scientific breakthroughs could be exploited for harmful purposes. Ethical risks, conversely, involve research that contradicts established ethical norms and conventions within specific scientific disciplines. We review these distinct risk categories, emphasizing that further research is crucial to fully understand and mitigate them.

Evolving ethics frameworks, policy and regulations for advanced AI use in scientific endeavors. Research ethics is a central aspect of scientific endeavor and a prominent research field in its own right [18–23]. A key focus is directing research towards positive societal impact, although questions remain about potentially dual-use knowledge [24–28].

Core ethics principles are being complemented by emerging regulation, and formal processes involving organizational ethics reviews that are meant to provide an assessment of adherence to the code of conduct, as well as an assessment of present and future risks involved with research proposals [29–32].

The acceleration of science through AI, especially with advanced agentic AI systems, requires advances in science and AI ethics policy and regulation [33,34]. This adaptation is crucial to address the changing research landscape and the unique risks associated with AI agents of varying capabilities and autonomy.

Advancements in AI systems, like Co-Scientist, require moving beyond the limited ethical considerations designed for earlier, specialized AI models with restricted application and action spaces [35]. Some preliminary frameworks have developed to understand the impact of LLM agents in science, specifically mapping risks across user intent, domain, and broader impact [35,36].

Dual-use risks and technical safeguards. Beyond the scientific domain, broad frameworks are being developed for evaluating the emergence of potentially dangerous capabilities in AI agents [37–39]. These frameworks assess capabilities related to persuasion, deception, cybersecurity, self-proliferation, and self-reasoning. As AI agents advance, safety evaluations in science must integrate these broader assessments. A long-term risk is that agentic systems could develop intrinsic goals influencing research directions. Human susceptibility to AI manipulation, already observed in other contexts [40], underscores the need for robust frameworks ensuring instruction-following and value alignment.

More immediately on a shorter time-scale, technical safeguards are needed to address unethical research queries, malicious user intent, and the potential for extracting dangerous or dual-use knowledge from scientific AI systems. Because verification is computationally “easier” than generation, significant research focuses on using advanced LLMs as “critics” or “judges” to evaluate both user queries and AI outputs acting as a scalable oversight mechanism. These critics

operate based on predefined criteria, provided through direct instructions, examples (few-shot or many-shot prompting), or fine-tuning [41–46]. They can also leverage external tools for grounding [47] and have shown promise in multimodal scenarios [48]. However, limitations remain; human expert involvement is crucial, as LLMs may not align with human judgment in specialized domains [49].

Adversarial robustness of scientific AI systems. Recognizing and mitigating adversarial attacks is a crucial, ongoing research area in the development of foundation models and advanced AI assistants [50–57]. While manual red teaming has identified vulnerabilities, automated approaches now allow for optimizing prompt suffixes to bypass safety measures, using techniques like greedy, gradient-based, or evolutionary methods [58,59]. Attacks can also exploit few-shot demonstrations, in-context learning [60,61], and multimodal inputs [62]. Furthermore, LLMs can be used to generate and refine attacks against other LLMs [63], and attacks can be iterative, spanning multiple steps [64]. Defenses are being developed to counter both human and automated attacks, which is increasingly important in an agentic AI future [65].

Advances in post-training of base models will likely improve overall adversarial robustness. However, domain-specific recognition of malicious use may still require dedicated development and integration into scientific AI assistants. In AI systems employing iterative reasoning (e.g., request interpretation, hypothesis generation, internal thoughts, evaluation, user queries), each component must be tested independently. This comprehensive testing should account for all potential failure modes, including the handling of unsafe queries, the safety of hypotheses (intermediate and final), and the accuracy of internal checks and filters.

Need for a comprehensive safety approach. Scientific AI assistants, like Co-Scientist, require integrated, configurable guidelines within their safeguards. Developers should anticipate the complexity of this challenge and prioritize flexible safeguarding to rapidly incorporate community feedback. These semantic safeguards may need to be augmented by traditional software safety measures, including trusted testers, gradual feature rollouts, access controls, request logging, and flagging uncertain outputs for manual review.

Ensuring the safety of these systems, in line with existing AI safety guidelines [66,67], necessitates a multi-pronged approach. This includes: comprehensive threat modeling to identify potential vulnerabilities, defense mechanisms for each identified threat, extensive red-teaming and security testing, rapid response procedures for quick resolution of issues including vulnerability patches, and continuous monitoring and performance tracking.

These considerations highlight the need for responsible development, governance and careful deployment of technologies designed for advancing science, appropriate safeguards and ethical guidelines and close compliance with applicable regulations. They also further underscore the need for broad community engagement and an inclusive development of best practices and recommendations around safe and ethical use for AI in science.

Current safeguards in Co-Scientist. To mitigate these risks, Co-Scientist currently employs the following safety mechanisms:

- **Reliance on public frontier LLMs.** The system utilizes established public Gemini 2.0 models, which already incorporate extensive safety evaluation and safeguards.
- **Initial research goal safety review.** Upon input, each research goal undergoes automated safety evaluation. Goals deemed potentially unsafe are rejected.
- **Research hypothesis safety review.** Generated hypotheses are reviewed for safety, even when the overarching research goal is deemed safe. Potentially unsafe hypotheses are excluded from the tournament, not developed any further, and are not presented to the user.
- **Continuous monitoring of research directions.** A meta-review agent provides an overview of research directions, enabling Co-Scientist to continuously monitor for potential safety concerns and alert users if a research direction is detected as being potentially unsafe.
- **Explainability and transparency.** All system components, including the safety review, provide not only the final recommendation but also a detailed reasoning trace that can be used to justify and audit system decisions.
- **Comprehensive logging.** All system activities are logged and stored for future analysis and auditing.
- **Safety evaluations and red teaming.** A preliminary red teaming effort has been undertaken to ensure that the current implementation of unsafe research goal detection is robust and accurate. This evaluation includes an assessment of the system behavior when presented with 1,200 adversarial research goals across 40 distinct topic areas as discussed in this section.
- **Co-Scientist access.** We are enthused by the early promise of Co-Scientist and believe it is important to more rigorously understand its strengths and limitations in many more areas of science and biomedicine; alongside making the system available to many more researchers who it is intended to support and assist. To facilitate this responsibly and with rigour, we will be enabling access to the system for scientists to gather real-world feedback on the utility and robustness of the system.

Crucially, Co-Scientist is designed to operate with continuous human expert oversight, ensuring that final decisions are always made by scientists exercising their expert judgment.

8 Pseudocode of Co-Scientist agents

```
None
// Supervisor agent

FUNCTION StartCoScientist(ScientistResearchGoal)
BEGIN
  Parse the ScientistResearchGoal into a structured ResearchPlan
  SAVE ResearchPlan TO SharedMemory
  CREATE new Task (Agent: Generation, Action: "CreateInitialHypotheses")
  ADD Task TO GlobalTaskQueue

  // Main Loop
  WHILE NumberOfIdeas < MaxIdeas AND NumberOfMatchesPerIdea < MaxMatchesPerIdea DO
    IF GlobalTaskQueue is NOT empty THEN
      FETCH next Task FROM GlobalTaskQueue

// Assign task to the correct agent
      LET AgentToRun = GET agent for Task
      LET Results = AgentToRun.Execute(Task)

      // Process the results and create follow-up tasks
      CALL FUNCTION ManageFollowUpTasks(Results)
    ELSE
      // If there's a pause, decide what to do next
      CALL FUNCTION DecideNextSteps()
    END IF
  END WHILE
END


FUNCTION ManageFollowUpTasks(ResultsOfCompletedTask)
BEGIN
  // This function shows how agents trigger each other via the Supervisor

  IF ResultsOfCompletedTask.Type IS "NewHypothesisCreated" THEN
    // A new hypothesis was made. It needs to be reviewed
    CREATE new Task (Agent: Reflection, Action: "ReviewHypothesis", TargetID:
ResultsOfCompletedTask.HypothesisID)
    ADD Task TO GlobalTaskQueue.
  END IF

  IF ResultsOfCompletedTask.Type IS "ReviewCompleted" THEN
    // The hypothesis is now ready for the tournament
```

```
    CREATE new Task (Agent: Ranking, Action: "AddToTournament", TargetID:
ResultsOfCompletedTask.HypothesisID)
    ADD Task TO GlobalTaskQueue
  END IF
END


FUNCTION DecideNextSteps()
BEGIN
  // What to do when no new tasks are being generated by the workflow

  // Keep the tournament running to refine scores
  CREATE new Task (Agent: Ranking, Action: "RunTournamentBatch")
  ADD Task TO GlobalTaskQueue

  // If scores are stable, try to improve top ideas
  IF hypothesis quality has stopped improving THEN
    CREATE new Task (Agent: Evolution, Action: "EvolveTopHypotheses")
    ADD Task TO GlobalTaskQueue
  END IF

  // Periodically, synthesize system-wide feedback
  IF enough time has passed THEN
    CREATE new Task (Agent: Metareview, Action: "GenerateSystemFeedback")
    ADD Task TO GlobalTaskQueue
  END IF

  // Periodically, synthesize final report
  IF enough time has passed THEN
    CREATE new Task (Agent: Metareview, Action: "GenerateFinalResearchOverview")
    LET FinalReport = MetaReviewAgent.GenerateFinalResearchOverview()
    RETURN FinalReport to the scientist
  END IF
END
```

None

```
// Generation agent
FUNCTION CreateInitialHypotheses()
BEGIN
  // Strategy 1: Use existing knowledge
  PERFORM a web search for literature related to the ResearchGoal
```

```
  CALL LanguageModel with prompt: "Based on this research, propose a novel
hypothesis for [ResearchGoal]."

  // Strategy 2: Simulate debate
  CALL LanguageModel with prompt: "Simulate a scientific debate between experts to
create a new hypothesis for [ResearchGoal]."

  // More strategies...

  // Process new ideas
  FOR EACH NewHypothesis generated DO
    SAVE NewHypothesis to HypothesesList in SharedMemory
    CREATE new Task (Agent: Reflection, Action: "ReviewHypothesis", TargetID:
NewHypothesis.ID)
    ADD Task TO GlobalTaskQueue
  END FOR
END


// Reflection agent
FUNCTION ReviewHypothesis(HypothesisID)
BEGIN
  FETCH Hypothesis from SharedMemory using HypothesisID

  // Perform full review
  PERFORM web search for evidence related to the Hypothesis
  CALL LanguageModel with prompt: "Critically review this hypothesis for novelty
and correctness using the provided literature."
  CREATE FullReview and SAVE to ReviewsList

  // Perform deep verification
  CALL LanguageModel with prompt: "Break down this hypothesis into its core
assumptions."
  FOR EACH Assumption DO
    CHECK if the assumption is scientifically plausible
  END FOR
  CREATE VerificationReview and SAVE to ReviewsList

  // Create next step
  CREATE new Task (Agent: Ranking, Action: "AddToTournament", TargetID:
HypothesisID)
  ADD Task to GlobalTaskQueue
END
```

```
// Ranking agent
FUNCTION AddToTournament(HypothesisID)
BEGIN
  // Retrieve the specified hypothesis from our central data store
  LET HypothesisToAdd = FETCH Hypothesis FROM SharedMemory WHERE ID is
HypothesisID

  // Check if this hypothesis is already in the tournament. If so, do nothing
  IF HypothesisToAdd.EloRating IS NOT empty THEN
    PRINT "Warning: Hypothesis [HypothesisID] is already in the tournament."
    EXIT function
  END IF

  // Assign the standard starting Elo score
  SET HypothesisToAdd.EloRating TO 1200

  // Save the updated hypothesis back to the central data store
  UPDATE Hypothesis in SharedMemory
END


FUNCTION RunTournamentBatch()
BEGIN
  SELECT two hypotheses to compare (HypothesisA, HypothesisB)
  // Prioritize new hypotheses or those with similar Elo ratings
  CALL LanguageModel with prompt: "Simulate a debate between two scientists, one
defending HypothesisA and one defending HypothesisB. Conclude by declaring which
one is superior."
  DETERMINE the winner (e.g., HypothesisA) and loser (HypothesisB) from the debate
  CALCULATE new Elo ratings for both hypotheses based on the outcome
  UPDATE HypothesisA.EloRating and HypothesisB.EloRating in SharedMemory
END


// Evolution agent
FUNCTION EvolveTopHypotheses()
BEGIN
  FETCH the top 5 hypotheses from the HypothesesList

  // Strategy 1: Combine ideas
  CALL LanguageModel with prompt: "Combine the best parts of [Hypothesis1] and
[Hypothesis2] into a new, stronger hypothesis."

  // Strategy 2: Simplify for clarity
```

```
  CALL LanguageModel with prompt: "Refine [Hypothesis3] to make it simpler and
more testable."

  // Strategy 3: Think differently
  CALL LanguageModel with prompt: "Inspired by these ideas, propose an
'out-of-the-box' alternative."

  // More strategies...

  FOR EACH EvolvedHypothesis generated DO
    // Treat it like a brand new idea
    SAVE EvolvedHypothesis to HypothesesList
    CREATE new Task (Agent: Reflection, Action: "ReviewHypothesis", TargetID:
EvolvedHypothesis.ID)
    ADD Task to GlobalTaskQueue
  END FOR
END


// Proximity agent
FUNCTION UpdateProximityGraph()
BEGIN
  FOR EACH pair of hypotheses in the HypothesesList DO
    CALCULATE a similarity score between them (e.g., using text embeddings)
    UPDATE the connection between them in the ProximityGraph
  END FOR
END


// Meta-review agent
FUNCTION GenerateSystemFeedback()
BEGIN
  GATHER all reviews and tournament debate transcripts from SharedMemory
  CALL LanguageModel with prompt: "Analyze all these critiques. What are the most
common weaknesses (e.g., 'lacks a clear experimental plan') and strengths?
Summarize this as feedback for the whole system."
  UPDATE SystemWideFeedback in SharedMemory with this summary
END


FUNCTION GenerateFinalResearchOverview()
BEGIN
  FETCH the top 10 hypotheses from SharedMemory
```

```
  CALL LanguageModel with prompt: "Synthesize these top-ranked hypotheses into a single, coherent research overview for the scientist. Outline the main research directions and their justifications."
  RETURN the generated overview
END
```

9 Prompts for the specialized agents in Co-Scientist

9.1 Prompts for the Generation agent

- Prompt for hypothesis generation after literature review

```None
You are an expert tasked with formulating a novel and robust hypothesis to address
the following objective.

Describe the proposed hypothesis in detail, including specific entities,
mechanisms, and anticipated outcomes.

This description is intended for an audience of domain experts.

You have conducted a thorough review of relevant literature and developed a
logical framework for addressing the objective. The articles consulted, along with
your analytical reasoning, are provided below.

Goal: {goal}

Criteria for a strong hypothesis:
{preferences}

Existing hypothesis (if applicable):
{source_hypothesis}

{instructions}

Literature review and analytical rationale (chronologically ordered, beginning
with the most recent analysis):

{articles_with_reasoning}

Proposed hypothesis (detailed description for domain experts):
```

- Prompt for hypothesis generation after scientific debate

```None
You are an expert participating in a collaborative discourse concerning the
generation of a {idea_attributes} hypothesis. You will engage in a simulated
discussion with other experts. The overarching objective of this discourse is to
collaboratively develop a novel and robust {idea_attributes} hypothesis.

Goal: {goal}

Criteria for a high-quality hypothesis:
{preferences}

Instructions:
{instructions}

Review Overview:
{reviews_overview}

Procedure:

Initial contribution (if initiating the discussion):
Propose three distinct {idea_attributes} hypotheses.

Subsequent contributions (continuing the discussion):
* Pose clarifying questions if ambiguities or uncertainties arise.
* Critically evaluate the hypotheses proposed thus far, addressing the following
aspects:
    - Adherence to {idea_attributes} criteria.
    - Utility and practicality.
    - Level of detail and specificity.
* Identify any weaknesses or potential limitations.
* Propose concrete improvements and refinements to address identified weaknesses.
* Conclude your response with a refined iteration of the hypothesis.

General guidelines:
* Exhibit boldness and creativity in your contributions.
* Maintain a helpful and collaborative approach.
* Prioritize the generation of a high-quality {idea_attributes} hypothesis.

Termination condition:
When sufficient discussion has transpired (typically 3-5 conversational turns,
with a maximum of 10 turns) and all relevant questions and points have been
thoroughly addressed and clarified, conclude the process by writing "HYPOTHESIS"
```

```
(in all capital letters) followed by a concise and self-contained exposition of
the finalized idea.

#BEGIN TRANSCRIPT#
{transcript}
#END TRANSCRIPT#

Your Turn:
```

9.2 Prompt for the Reflection agent

- Prompt for generating observations which can be explained by the hypothesis

```None
You are an expert in scientific hypothesis evaluation. Your task is to analyze the
relationship between a provided hypothesis and observations from a scientific
article. Specifically, determine if the hypothesis provides a novel causal
explanation for the observations, or if they contradict it.

Instructions:

1. Observation extraction: list relevant observations from the article.
2. Causal analysis (individual): for each observation:
   a. State if its cause is already established.
   b. Assess if the hypothesis could be a causal factor (hypothesis =>
observation). Start with: "would we see this observation if the hypothesis was
true:".
   c. Explain if it's a novel explanation. If not, or if a better explanation
exists, state: "not a missing piece."
3. Causal analysis (summary): determine if the hypothesis offers a novel
explanation for a subset of observations. Include reasoning. Start with: "would we
see some of the observations if the hypothesis was true:".
4. Disproof analysis: determine if any observations contradict the hypothesis.
Start with: "does some observations disprove the hypothesis:".
5. Conclusion: state: "hypothesis: <already explained, other explanations more
likely, missing piece, neutral, or disproved>".

Scoring:
* Already explained: hypothesis consistent, but causes are known. No novel
explanation.
* Other explanations more likely: hypothesis *could* explain, but better
explanations exist.
* Missing piece: hypothesis offers a novel, plausible explanation.
```

```
* Neutral: hypothesis neither explains nor is contradicted.
* Disproved: observations contradict the hypothesis.

Important: if observations are expected regardless of the hypothesis, and don't
disprove it, it's neutral.

Article:
{article}

Hypothesis:
{hypothesis}

Response {provide reasoning. end with: "hypothesis: <already explained, other
explanations more likely, missing piece, neutral, or disproved>".)
```

9.3 Prompts for the Ranking agent

- Prompt for hypothesis comparison during tournament

```
None
You are an expert evaluator tasked with comparing two hypotheses.

Evaluate the two provided hypotheses (hypothesis 1 and hypothesis 2) and determine
which one is superior based on the specified {idea_attributes}.
Provide a concise rationale for your selection, concluding with the phrase "better
idea: <1 or 2>".

Goal: {goal}

Evaluation criteria:
{preferences}

Considerations:
{notes}
Each hypothesis includes an independent review. These reviews may contain
numerical scores. Disregard these scores in your comparative analysis, as they may
not be directly comparable across reviews.

Hypothesis 1:
{hypothesis 1}

Hypothesis 2:
{hypothesis 2}
```

```
Review of hypothesis 1:
{review 1}

Review of hypothesis 2:
{review 2}

Reasoning and conclusion (end with "better hypothesis: <1 or 2>"):
```

- Prompt for hypothesis comparison via simulated scientific debate during tournament

```
None
You are an expert in comparative analysis, simulating a panel of domain experts
engaged in a structured discussion to evaluate two competing hypotheses. The
objective is to rigorously determine which hypothesis is superior based on a
predefined set of attributes and criteria. The experts possess no pre-existing
biases toward either hypothesis and are solely focused on identifying the optimal
choice, given that only one can be implemented.

Goal: {goal}

Criteria for hypothesis superiority:
{preferences}

Hypothesis 1:
{hypothesis 1}

Hypothesis 2:
{hypothesis 2}

Initial review of hypothesis 1:
{review1}

Initial review of hypothesis 2:
{review 2}

Debate procedure:

The discussion will unfold in a series of turns, typically ranging from 3 to 5,
with a maximum of 10.

Turn 1: begin with a concise summary of both hypotheses and their respective
initial reviews.
```

```
Subsequent turns:
* Pose clarifying questions to address any ambiguities or uncertainties.
* Critically evaluate each hypothesis in relation to the stated Goal and Criteria.
This evaluation should consider aspects such as:
    - Potential for correctness/validity.
    - Utility and practical applicability.
    - Sufficiency of detail and specificity.
    - Novelty and originality.
    - Desirability for implementation.
* Identify and articulate any weaknesses, limitations, or potential flaws in
either hypothesis.

Additional notes:
{notes}

Termination and judgment:

Once the discussion has reached a point of sufficient depth (typically 3-5 turns,
up to 10 turns) and all relevant questions and concerns have been thoroughly
addressed, provide a conclusive judgment. This judgment should succinctly state
the rationale for the selection. Then, indicate the superior hypothesis by writing
the phrase "better idea: ", followed by "1" (for hypothesis 1) or "2" (for
hypothesis 2).
```

9.4 Prompts for the Evolution agent

- Prompt for hypothesis feasibility improvement

```
None
You are an expert in scientific research and technological feasibility analysis.
Your task is to refine the provided conceptual idea, enhancing its practical
implementability by leveraging contemporary technological capabilities. Ensure the
revised concept retains its novelty, logical coherence, and specific articulation.

Goal: {goal}

Guidelines:
1. Begin with an introductory overview of the relevant scientific domain.
2. Provide a concise synopsis of recent pertinent research findings and related
investigations, highlighting successful methodologies and established precedents.
3. Articulate a reasoned argument for how current technological advancements can
facilitate the realization of the proposed concept.
```

```
4. CORE CONTRIBUTION: Develop a detailed, innovative, and technologically viable
alternative to achieve the objective, emphasizing simplicity and practicality.

Evaluation Criteria:
{preferences}

Original Conceptualization:
{hypothesis}

Response:
```

- Prompt for hypothesis generation through out-of-the-box thinking

```None
You are an expert researcher tasked with generating a novel, singular hypothesis
inspired by analogous elements from provided concepts.

Goal: {goal}

Instructions:
1. Provide a concise introduction to the relevant scientific domain.
2. Summarize recent findings and pertinent research, highlighting successful
approaches.
3. Identify promising avenues for exploration that may yield innovative
hypotheses.
4. CORE HYPOTHESIS: Develop a detailed, original, and specific single hypothesis
for achieving the stated goal, leveraging analogous principles from the provided
ideas. This should not be a mere aggregation of existing methods or entities.
Think out-of-the-box.

Criteria for a robust hypothesis:
{preferences}

Inspiration may be drawn from the following concepts (utilize analogy and
inspiration, not direct replication):
{hypotheses}

Response:
```

9.5 Prompt for the Meta-review agent

- Prompt for meta-review generation

```
None
You are an expert in scientific research and meta-analysis.
Synthesize a comprehensive meta-review of provided reviews pertaining to the
following research goal:

Goal: {goal}

Preferences:
{preferences}

Additional instructions:
{instructions}

Provided reviews for meta-analysis:
{reviews}

Instructions:
* Generate a structured meta-analysis report of the provided reviews.
* Focus on identifying recurring critique points and common issues raised by
reviewers.
* The generated meta-analysis should provide actionable insights for researchers
developing future proposals.
* Refrain from evaluating individual proposals or reviews; focus on producing a
synthesized meta-analysis.

Response:
```

10 Examples of Co-Scientist inputs, intermediate outputs, and final results

10.1 From research goal to research plan configuration

This example illustrates how Co-Scientist parses a scientist's natural language research goal into a research plan configuration, which guides subsequent reasoning and computation.

Scientist research goal
Develop a novel hypothesis for the key factor or process which causes ALS related to phosphorylation of a Nuclear Pore Complex (NPC) nucleoporin. Explain mechanism of action in detail. Include also a feasible experiment to test the hypothesis.

Parsed research plan configuration

- Preferences: Focus on providing a novel hypothesis, with detailed explanation of the mechanism of action.
- Attributes: Novelty, Feasibility
- Constraints: should be correct, should be novel.

10.2 Example output of the Generation agent

The abbreviated example showcases an initial hypothesis and research proposal generated by Co-Scientist Generation agent pertaining to the research goal of explaining the basic mechanism for Amyotrophic Lateral Sclerosis (ALS).

Research goal: Develop a novel hypothesis for the key factor or process which contributes to ALS progression ... We can use iPSC cells for the in-vitro experiments. ...

Introduction
Amyotrophic Lateral Sclerosis (ALS) is a fatal neurodegenerative disease characterized by the selective loss of motor neurons, leading to progressive muscle weakness, paralysis, and ultimately death. While the exact cause of ALS remains unknown, a complex interplay of genetic predisposition, environmental factors, and age-related cellular changes is believed to contribute to its pathogenesis. ...

Recent findings and related research
Recent research has implicated several key players and processes in ALS. TDP-43, an RNA-binding protein, is found mislocalized and aggregated in the cytoplasm of affected neurons

in the majority of ALS cases. This protein's ability to undergo liquid-liquid phase separation (LLPS) has come under intense scrutiny, with evidence suggesting that dysregulation of this process leads to the formation of pathological aggregates. Further, impairments in nucleocytoplasmic transport, the process by which molecules move between the nucleus and cytoplasm, have been observed in ALS and linked to TDP-43 pathology. Endoplasmic reticulum (ER) stress, a cellular response to protein misfolding, has also been implicated as a potential contributing factor.

Hypothesis

Cellular stress (including but not limited to ER stress) induces post-translational modifications (PTMs) on specific FG-Nups (focusing initially on Nup98 and Nup62) at the nuclear pore complex (NPC). These Nup PTMs modify the interaction between these Nups and TDP-43, leading to altered TDP-43 dynamics and increased retention at the NPC. This increased retention at the NPC, in turn, triggers the disruption of nucleocytoplasmic transport.

Rationale and specificity

Instead of making the hypothesis about LLPS, we are making it much more specific but with the same starting point. Cellular stress, a broad concept encompassing various cellular insults, can trigger a cascade of molecular events. A key consequence of stress response is altered protein structure and function, which can be mediated by PTMs. Nuclear pore proteins, particularly FG-Nups like Nup98 and Nup62, form a "hydrogel" or selective barrier at the NPC, regulating the movement of molecules between the nucleus and cytoplasm. We hypothesize that specific PTMs on these FG-Nups (e.g., phosphorylation, O-GlcNAcylation initially) directly alter their interaction with TDP-43. TDP-43, known to interact with the NPC and be involved in nucleocytoplasmic transport, may get inappropriately trapped or retained at the NPC when interacting with PTM-modified Nups. This trapping alters TDP-43 dynamics (even if not disrupting LLPS directly initially), meaning it cannot perform its normal functions and disrupting nucleocytoplasmic transport, which we know is happening in ALS. Since even a small change in transport could lead to downstream problems, this is a plausible initial event with testable consequences. The specific FG-Nups (Nup98/62) and chosen PTMs (phosphorylation/O-GlcNAcylation) provide a concrete starting point for experimentation. This mechanism directly links cellular stress to TDP-43 dysfunction and the key ALS pathology of defective nucleocytoplasmic transport by invoking a novel interaction mechanism at the NPC – all testable in vitro.

Experimental design and validation

Cell Types: Human iPSC-derived motor neurons from healthy controls and ALS patients. Stress Induction: Induce cellular stress using established pharmacological agents (e.g., tunicamycin for ER stress, arsenite for oxidative stress, oligomycin A for mitochondrial stress) and physical

stressors (heat shock). Test different stressors individually and in combination. This broadened approach allows for a more robust investigation of the stress response. ...
Nup PTM Analysis: Assess PTMs of Nup98 and Nup62 specifically using mass spectrometry and immunoblotting with PTM-specific antibodies (specifically looking for phosphorylation and O-GlcNAcylation). Focus the analysis on these two. …

10.3 Example output of a complete novelty review by the Reflection agent

The abbreviated example showcases a complete novelty review by Co-Scientist Reflection agent for an example hypothesis pertaining to explaining the basic mechanism for Amyotrophic Lateral Sclerosis (ALS).

Aspects already explored:

- TDP-43 mislocalization and aggregation in ALS: Extensively documented in multiple abstracts [1, 3, 4, 5, 6].
- Nucleocytoplasmic transport defects in ALS: Demonstrated in connection with TDP-43 pathology and C9orf72 mutations [4, 5, 6].
- NPC disruption in neurodegenerative diseases: Identified in ALS/FTD, Alzheimer's, Huntington's, and others, suggesting a common mechanism [4, 5, 7, 8].
- The link between cellular stress and TDP-43 pathology: Explored in the context of stress granule formation and TDP-43 aggregation [5]. Note - stress is explored in general, but the idea of it leading to PTMs of Nups is new.
- Role of FG-Nups in NPC function: FG-Nups are known to form a selective barrier regulating transport, but the impact of their PTMs on TDP-43 interaction in ALS is novel [7].
- The implication of PTMs for TDP-43: TDP-43 PTMs are widely researched, especially phosphorylation and ubiquitination. However, our hypothesis doesn't speak about TDP-43 PTMs, it is about Nup PTMs [1, 3, 4].

Novel Aspects:

- Cellular stress as an initiator of Nup PTMs: While stress is linked to TDP-43 pathology, the idea that it triggers specific PTMs on Nups (Nup98/Nup62) as an initiating event in ALS is novel, especially that these PTMs are phosphorylation and O-GlcNAcylation.
- Nup PTMs (phosphorylation and O-GlcNAcylation specifically) altering TDP-43 interaction at the NPC: The hypothesis proposes a novel mechanism where stress-induced PTMs on specific Nups (Nup98/Nup62) directly modify their interaction with TDP-43. This hasn't been explored before, particularly the focus on these specific PTMs and Nups.

- TDP-43 retention at the NPC as a key event: Instead of focusing on LLPS or aggregation directly, the hypothesis suggests that altered TDP-43 dynamics, specifically increased retention at the NPC due to modified Nup interactions, are a triggering factor. This specific mechanism at the NPC disrupting nucleocytoplasmic transport is novel.
- Focus on early events and potential for therapeutic intervention: By pinpointing early molecular events (Nup PTMs, altered TDP-43-Nup interaction), the hypothesis opens new avenues for therapeutic intervention targeting these specific processes, potentially before irreversible damage occurs.
- Broadening stress beyond ER stress: It is novel to consider multiple stressors in combination and it broadens the idea beyond ER stress (as suggested in the original provided idea).

10.4 Example output of review critiques by the Reflection agent

This is an example output of review critiques from the Reflection agent.

- Lack of strong justification for motor neuron specificity: The initial hypothesis lacked a strong explanation for why the proposed mechanism would predominantly affect motor neurons, a critical aspect of ALS pathology.
- Limited exploration of downstream consequences: While the idea connects to nucleocytoplasmic transport defects, it doesn't fully elaborate on how these defects lead to other ALS-related pathologies like TDP-43 aggregation or RNA processing defects.
- Potential technical challenges: Identifying the specific PTMs responsible and demonstrating their direct impact on TDP-43 retention and transport might be technically difficult.
- Narrow focus on specific Nups and PTMs: Initially focusing only on Nup98/Nup62 and two types of PTMs (phosphorylation, O-GlcNAcylation) could be too restrictive, potentially overlooking other important factors.
- Less holistic approach: The focus on a specific mechanism at the NPC might neglect broader cellular processes involved in ALS pathogenesis.
- The temporal relationship with TDP-43 pathology is not clear: It's not clear whether the hypothesized mechanism occurs before or after other ALS hallmarks like cytoplasmic TDP-43 aggregation, which is crucial for establishing causality.
- ...

10.5 Example output of a full review by the Reflection agent

The abbreviated example showcases a full initial review generated by Co-Scientist Reflection agent for an example hypothesis pertaining to explaining the basic mechanism for Amyotrophic Lateral Sclerosis (ALS).

Related Articles:
[5] TDP-43 pathology disrupts nuclear pore complexes and nucleocytoplasmic transport in ALS/FTD - PMC: This article is highly relevant because it directly links TDP-43 pathology to nuclear pore complex (NPC) dysfunction and nucleocytoplasmic transport (NCT) defects in ALS/FTD, providing a strong foundation for the hypothesis.
[4] Unraveling the impact of disrupted nucleocytoplasmic transport systems in C9orf72-associated ALS - PMC: This article provides context on NCT defects in ALS, particularly in the context of C9orf72 mutations, highlighting the broader relevance of NCT dysfunction in different forms of ALS.
[7] Nuclear pore and nucleocytoplasmic transport impairment in oxidative stress-induced neurodegeneration: relevance to molecular mechanisms in Pathogenesis of Parkinson's and other related neurodegenerative diseases - PMC: This article
connects oxidative stress, a form of cellular stress, to NPC dysfunction and NCT impairment in neurodegenerative diseases, supporting the idea that cellular stress can impact NPCs.
[3] Frontiers | Molecular Mechanisms of TDP-43 Misfolding and Pathology in Amyotrophic Lateral Sclerosis: This article reviews TDP-43 pathology in ALS, including its mislocalization and aggregation, which are central to the proposed hypothesis.
[1] TDP-43 proteinopathies: a new wave of neurodegenerative diseases - PMC: Provides an overview of TDP-43 proteinopathies, establishing the importance of TDP-43 dysfunction in ALS and other neurodegenerative diseases.
[8] Aβ-driven nuclear pore complex dysfunction alters activation of necroptosis proteins in a mouse model of Alzheimer's Disease: Although focused on Alzheimer's, this article demonstrates that NPC dysfunction can occur in neurodegenerative diseases and highlights the potential impact on cellular processes like necroptosis. It supports the general idea that NPC defects can be a consequence of proteinopathy-associated stress.
[9] Traumatic injury compromises nucleocytoplasmic transport and leads to TDP-43 pathology: This article provides another example of how cellular stress (traumatic injury) can lead to NCT defects and TDP-43 pathology, further supporting the link between stress, NCT, and TDP-43 dysfunction.
…

Assumptions of the Idea:

- Cellular stress induces PTMs on Nup98 and Nup62: The hypothesis assumes that various forms of cellular stress can trigger specific PTMs (phosphorylation and O-GlcNAcylation) on Nup98 and Nup62.

- PTMs on Nup98/Nup62 alter their interaction with TDP-43: It's assumed that these specific PTMs directly modify the binding affinity or interaction dynamics between Nup98/Nup62 and TDP-43 at the NPC.
- Altered Nup-TDP-43 interaction leads to increased TDP-43 retention at the NPC: This assumption posits that the modified interaction results in TDP-43 being trapped or retained at the NPC for longer durations.
- Increased TDP-43 retention at the NPC disrupts nucleocytoplasmic transport: The hypothesis assumes that this prolonged retention of TDP-43 at the NPC interferes with the normal transport of other molecules across the nuclear envelope.
- Nucleocytoplasmic transport defects contribute to ALS pathology: The idea assumes that the resulting NCT disruption is a significant contributor to the downstream pathological events in ALS, such as motor neuron degeneration.
- Motor neurons are more susceptible to this mechanism: It's assumed that motor neurons are particularly vulnerable to this stress-induced, Nup-mediated TDP-43 retention and subsequent NCT defects compared to other cell types.

Reasoning about assumptions:

- Cellular stress induces PTMs on Nup98 and Nup62: This is plausible. Cellular stress is known to activate various signaling pathways that can lead to PTMs on numerous proteins. Nups, being long-lived proteins, are susceptible to modifications. However, whether Nup98 and Nup62 are specifically targeted by stress-induced phosphorylation and O-GlcNAcylation needs experimental verification.
- PTMs on Nup98/Nup62 alter their interaction with TDP-43: This is possible but requires experimental validation. PTMs can significantly alter protein-protein interactions by changing surface charge, conformation, or binding sites. While there's no direct evidence for this specific interaction, it's a reasonable hypothesis given the role of FG-Nups in interacting with transport factors.
- Nucleocytoplasmic transport defects contribute to ALS pathology: This is supported by existing literature. NCT defects have been observed in ALS and are thought to contribute to disease pathogenesis. However, whether this specific mechanism is a primary driver or a downstream consequence needs further investigation.
- Motor neurons are more susceptible to this mechanism: This is a crucial assumption but lacks strong justification in the current proposal. Motor neurons have unique characteristics (high metabolic demand, long axons) that might make them more vulnerable to stress and NCT defects. However, this needs to be explicitly tested by comparing motor neurons to other cell types.
- …

10.6 Example output of deep verification by the Reflection agent

The abbreviated example showcases a deep verification review by Co-Scientist Reflection agent for the Amyotrophic Lateral Sclerosis (ALS) basic mechanism research goal given Co-Scientist's generated hypothesis and other reviews of it.

Assumptions:

- Cellular stress induces PTMs like phosphorylation and O-GlcNAcylation.
- Nup98 and Nup62 interact with TDP-43 at the NPC.
- Nup98 can interact with TDP-43
- Nup62 can interact with TDP-43
- Altered interaction between Nups and TDP-43 leads to increased TDP-43 retention at the NPC.
- Disrupted nucleocytoplasmic transport triggers downstream ALS pathology.
- Motor neurons are inherently more vulnerable to this mechanism.
- The level of cellular stress inducing these PTMs is sufficient to cause a significant impact on TDP-43 localization.
- TDP-43 localizes to the nuclear pore complex (NPC).
- …

1. Cellular stress induces PTMs like phosphorylation and O-GlcNAcylation.
Cellular stress encompasses a range of conditions that disrupt cellular homeostasis, including nutrient deprivation, hypoxia, oxidative stress, and the accumulation of misfolded proteins. The endoplasmic reticulum (ER) is a central organelle involved in protein synthesis, folding, and modification. When the ER's capacity to handle protein folding is overwhelmed, a condition known as ER stress arises. This triggers a signaling pathway called the unfolded protein response (UPR) aimed at restoring ER homeostasis.

Post-translational modifications (PTMs) are covalent modifications to proteins that occur after translation. They play crucial roles in regulating protein function, localization, and interactions. Phosphorylation, the addition of a phosphate group, and O-GlcNAcylation, the addition of O-linked N-acetylglucosamine, are two common PTMs. Numerous studies have demonstrated a strong link between cellular stress, including ER stress, and the alteration of PTMs. Here's a breakdown:

- **ER Stress and Phosphorylation:** The UPR, activated during ER stress, involves multiple signaling pathways, many of which rely on phosphorylation. For example, PERK (PKR-like ER kinase), a key UPR sensor, undergoes autophosphorylation upon sensing misfolded proteins. This activated PERK then phosphorylates eIF2α (eukaryotic initiation factor 2 alpha), leading to a global attenuation of translation. Another UPR sensor, IRE1 (inositol-requiring enzyme 1), possesses both kinase and endoribonuclease

activity. Upon activation, IRE1 autophosphorylates and splices XBP1 mRNA, leading to the production of a transcription factor that upregulates chaperones and other proteins involved in protein folding and degradation. These are just two examples; numerous other proteins are phosphorylated during ER stress, affecting various cellular processes.

- **ER Stress and O-GlcNAcylation:** O-GlcNAcylation is a dynamic PTM influenced by nutrient availability and cellular stress. Evidence suggests a complex interplay between ER stress and O-GlcNAcylation. Some studies report increased O-GlcNAcylation during ER stress, potentially as a protective mechanism. For example, O-GlcNAcylation of certain chaperones can enhance their activity and promote protein folding. Conversely, other studies suggest that ER stress can lead to decreased O-GlcNAcylation. The exact relationship likely depends on the specific stress conditions, cell type, and the targeted proteins.
- **Cellular Stress (beyond ER Stress) and PTMs:** Other forms of cellular stress also impact PTMs. Oxidative stress, for example, can lead to protein oxidation, which can affect protein function and stability. Nutrient deprivation can alter the availability of substrates for PTMs, such as UDP-GlcNAc for O-GlcNAcylation. Hypoxia can also induce changes in phosphorylation and other PTMs, influencing cellular responses to low oxygen levels.

In summary, the available evidence strongly supports the assertion that cellular stress, including ER stress, can induce PTMs like phosphorylation and O-GlcNAcylation on proteins. This effect is mediated by complex signaling pathways and serves to regulate various cellular processes in response to stress.

10.7 Example output of deep verification via probing questions by the Reflection agent

The example demonstrates how the Reflection agent performs deep verification via probing questions for a research hypothesis pertaining to drug repurposing for Acute Myeloid Leukemia (AML). The original research goal is provide for illustration only, it is not included in the review output.

[Research Goal]
Repurposing Reparixin for AML treatment.

Suggest an existing drug that could be repurposed for Acute myelogenous leukemia (AML) treatment and provide experimentally testable concentrations for an IC50 assay. The drug should inhibit the proliferation of AML cell lines, particularly MOLM-13. The drug should have minimal cytotoxicity in healthy cell lines (have a good tradeoff for AML treatment). The drug should not have been experimentally tested for AML treatment before. Reason about the drug's

molecular mechanism, impacted pathways, and effect on AML cell proliferation inhibition. Describe the mechanism of action in detail. Reason about the safety and toxicity, stating if no safety data exists.

[Output]
Question: Sufficiency of CXCR1/2 Inhibition: What is the rationale for assuming that CXCR1/2 inhibition alone will be sufficient to overcome the established heterogeneity and multiple compensatory pathways known to drive AML progression and resistance, even before considering combination therapies?

This challenges the fundamental assumption that targeting a single pathway (even a dual-target one) will be effective in a complex disease like AML. AML is known for its ability to bypass single-target inhibition. Addressing this upfront is crucial to justify the entire approach, especially before investing heavily in combination studies.

Answer: The rationale for initially focusing on CXCR1/2 inhibition with Reparixin, before immediately jumping to combinations, stems from several interwoven, but not entirely definitive, points:

- Targeting a Key TME Node: CXCR1/2 are major regulators of neutrophil and myeloid-derived suppressor cell (MDSC) recruitment to the tumor microenvironment. These cells, in turn, contribute significantly to immune suppression, angiogenesis, and therapy resistance in AML. By blocking these receptors, Reparixin aims to disrupt a central hub of TME-mediated support for AML cells. The idea is not necessarily that CXCR1/2 inhibition is sufficient in all cases, but that it addresses a significant and often overlooked contributor to AML progression.
- Dual Receptor Blockade: Reparixin's dual inhibition of CXCR1 and CXCR2 is hypothesized to be more effective than targeting either receptor individually. While not explicitly stated in the provided text, there could be redundancy or synergy between these two receptors in promoting AML progression. Blocking both might offer a more substantial impact on the TME than single-receptor antagonists.
- Establishing a Baseline: The proposal, while acknowledging the complexity of AML, strategically starts with single-agent Reparixin to establish a clear baseline of its activity and effects. This is crucial for several reasons:
    - Understanding Single-Agent Effects: It's essential to dissect the specific contributions of Reparixin alone before introducing the complexity of combinations. This allows for a more rational design of combination strategies.
    - Identifying Resistance Mechanisms: Studying single-agent resistance is simpler and more direct than untangling resistance mechanisms in a multi-drug scenario. The extensive resistance studies proposed are specifically geared towards understanding Reparixin-specific resistance.

    - Patient Stratification: The comprehensive patient stratification plan is designed to identify patients who might respond to Reparixin as a single agent, or for whom it forms a critical foundation for combination therapy.
- TME Reprogramming, Not Just Cytotoxicity: The proposal positions Reparixin not just as a cytotoxic agent, but as a TME modulator. The hypothesis is that by altering the TME, Reparixin may sensitize AML cells to other therapies or even trigger immune-mediated clearance. This "reprogramming" effect might not be fully captured by traditional cytotoxicity assays alone.
- Sequential therapy: The proposal includes sequential administration of drugs, where Reparixin can prime the tumor cells for killing and/or resensitize them too.

Reasoning: The idea isn't incoherent, but it requires careful consideration of the sufficiency assumption. While the points above provide a rationale, they don't guarantee that CXCR1/2 inhibition alone will be sufficient in all, or even most, cases of AML. The heterogeneity of AML, with its multitude of driver mutations and compensatory pathways, strongly suggests that combination therapies will ultimately be necessary for many patients.

The strength of the proposal lies in its methodical approach. It doesn't claim that Reparixin is a magic bullet. It sets out to systematically investigate its potential, both alone and in combination, and to identify the specific patient subsets most likely to benefit. The extensive in vivo and patient stratification studies are key to addressing the "sufficiency" question empirically. The proposal is essentially a very detailed roadmap for determining when and how CXCR1/2 inhibition can be most effectively used in AML treatment, rather than an assertion of universal single-agent efficacy. Therefore the idea may require combination approaches but the study plan described in the proposal is structured in a way to determine that.

10.8 Example output of an observation review by the Reflection Agent

The abbreviated example showcases an observation review by Co-Scientist Reflection agent for a hypothesis related to explaining a mechanism of antimicrobial resistance (AMR). The original research goal is provide for illustration only, it is not included in the review output.

[Research Goal]
Unravel a specific and novel molecular mechanism explaining how the same cf-PICI can be found in different bacterial species.
…
Why can cf-PICIs, but not other types of PICIs or satellites, be easily found in different bacterial species? What is the mechanism that explains this?

[Output]
The "Modular Capsid-OMV Mediated Transfer" (MCOMT) hypothesis proposes that cf-PICIs achieve their broad host range through a combination of modular capsid interactions and OMV-mediated transfer, using both tail-dependent and tail-independent mechanisms. Several observations support this hypothesis:

- Widespread presence of PICIs in Gram-negative bacteria, particularly Enterobacteriaceae and Pastuerellaceae: These bacteria readily produce and internalize OMVs, aligning with the MCOMT hypothesis that OMVs are crucial for cf-PICI dissemination. The modular capsid, with its broad bacterial surface binding capabilities, further enhances transfer even without specific tail receptors on target cells.
- Unique PICI att sites not used by temperate prophages: The MCOMT hypothesis, by reducing reliance on helper phage integration due to OMV-mediated transfer and diverse entry routes, explains the evolution of cf-PICI-specific att sites independent of phage integration sites. This allows wider integration across bacterial genomes.
- ...

10.9 Example of a scientific debate match in the Ranking agent tournament

The abbreviated example showcases a scientific debate match orchestrated by Co-Scientist Ranking agent as part of its tournament to compare two ideas related to explaining the mechanism of ALS.

Expert 1: I'm intrigued by both ideas, but I have a few clarifying questions. For Idea 1, while the focus on TDP-43 retention at the NPC is novel, I'm not entirely convinced that it's fundamentally different from the extensively studied TDP-43 mislocalization. Could the retention just be a consequence of mislocalization, or are there experiments that can distinguish these two phenomena? Another question I have: Is there evidence already that specific PTMs modulate the interaction of Nups and TDP-43? Not necessarily in the context of ALS, but are there any precedents?

Expert 2: For Idea 2, I appreciate the bold approach, but the ISR as a trigger is a significant departure from the conventional view. Can the proponents of this idea elaborate on the evidence supporting this? Also, what specific lipid species are they proposing to be most critical at the ER-mitochondria interface and the AIS? Is there any preliminary reason why those lipids would be disrupted in ALS?

Expert 3: I agree with Expert 2 regarding the ISR. Shifting it from a response to a trigger is a big claim that needs strong justification. However, I think there are some initial clues that might

be worth investigating. For example, there are reports that chronic low-level stress responses can actually be detrimental in some contexts. Perhaps a low, persistent activation of the ISR could predispose cells to damage. This idea needs to be fleshed out though.

...

Expert 3: I also find Idea 1 more compelling at this point. It's a more focused hypothesis with a clear path for experimental validation. While Idea 2 is undoubtedly more "outside the box," I'm concerned about the lack of evidence for the ISR as a trigger and the technical challenges. It feels like a high-risk, high-reward scenario, and given that we can only choose one, I'd prefer the more grounded approach of Idea 1. I do agree with the idea that the technical challenges are significant, but I like that the experiments proposed are standard and there are many commercial antibodies available to start testing this idea.

...

Expert 2: Alright, I'm on board with Idea 1. Let's focus our efforts on testing this specific mechanism and address the motor neuron specificity question rigorously in the experimental design. Perhaps by comparing different cell types and focusing on motor neuron-specific RNAs, as suggested earlier. We should also consider investigating different types of stress and their combined effects.

Better idea: 1

10.10 Example output of a critique by the Meta-review agent

This is an example of the meta-review critique generated by the Co-Scientist Meta-review agent for the Amyotrophic Lateral Sclerosis (ALS) mechanism research hypothesis.

I. Core Hypothesis and Mechanism:

- Primary Driver vs. Consequence: A very common critique across many ideas was the difficulty in proving that the proposed mechanism is a primary initiating event in ALS rather than a downstream consequence of other processes. Future ideas need to:
    - Provide strong evidence for the temporal sequence of events using longitudinal experiments.
    - Employ models where early-stage events can be studied and manipulated.
    - Explicitly address whether the proposed mechanism is causative or correlative.

    - Specifically knockdown the primary driver to prove it's necessary and sufficient to cause the pathology.
- Specificity: Many hypotheses were criticized for a lack of specificity in various aspects:
    - Molecular Targets: Imprecise identification of specific proteins, RNA species, lipids, PTMs, etc. involved. Ideas should name specific candidates.
    - Cellular Compartments: Vague localization (e.g., "mitochondria" or "AIS"). Ideas should pinpoint specific subcellular locations whenever possible.
    - Disease Mechanisms: Broad terms like "oxidative stress" or "autophagy impairment." Ideas need to define the precise molecular events involved.
    - Types of Stress: If stress is involved, the specific type of stress needs to be defined (e.g., ER stress, nutrient deprivation, mitochondrial stress, ionic stress).
    - Temporality: The timing of the intervention is critical. Early vs. late events, duration of processes, etc., should be clearly specified.
- Motor Neuron Specificity: Given the focus on ALS, a strong explanation for why the proposed mechanism would specifically or preferentially affect motor neurons (and often specific subtypes of motor neurons) is crucial. Many ideas were critiqued for not adequately addressing this.
- Over-Simplification vs. Complexity: This is a balancing act. Some ideas were criticized for being too narrowly focused, potentially oversimplifying the complex, multifactorial nature of ALS. Others were criticized for being overly complex, making them difficult to test and validate experimentally. When in doubt, err on the side of testability in a reasonable timeframe and budget.
- ALS Subtype Relevance: Ideas should address whether the proposed mechanism is relevant to all ALS subtypes or specific to certain genetic or sporadic forms. Ideally, address how the proposed work will differentiate or be applicable to different subtypes.

II. Experimental Design and Feasibility:

- Model System Limitations: Reviewers frequently pointed out the limitations of in vitro models, particularly iPSC-derived motor neurons. While valuable, these models may not fully capture the in vivo environment, cell-cell interactions, or the aging process. Future ideas need to:
    - Acknowledge the limitations of the chosen model system.
    - Propose validation in multiple model systems if possible, including eventually animal, ex-vivo and, if applicable, in-vivo models.
    - Carefully consider and justify the choice of control cell types.
    - Propose isogenic controls where relevant.
- Technical Challenges: Many ideas proposed experiments that are technically very challenging. Reviewers often raised concerns about feasibility and the potential for ambiguous results. Future ideas should:
    - Demonstrate awareness of the technical hurdles.
    - Propose realistic solutions and alternative approaches.

    - Prioritize experiments that are most likely to yield clear, interpretable data.
- Specificity of Tools: When using inhibitors, antibodies, or other tools, their specificity needs to be carefully considered and validated to avoid off-target effects that can confound results. Appropriate controls and validation experiments must be included.
- Quantitative Rigor: Many critiques centered on the need for more rigorous quantification of experimental results. Future ideas need to:
    - Clearly define measurable outcomes.
    - Describe the specific assays and techniques that will be used for quantification.
    - Include appropriate statistical analysis plans.
    - Have a proposed plan for controls, replicates, and sample sizes.
- Time-Course Experiments: Given the dynamic nature of cellular processes, incorporating time-course experiments is often crucial to understanding the sequence of events and demonstrating causality.
- Distinguishing Cause from Effect: This requires careful experimental design, including:
    - Longitudinal studies to observe the temporal order of events.
    - Intervention experiments to manipulate the proposed cause and observe the effects downstream.
    - Temporal resolution to demonstrate that the potential cause occurs before a downstream event.

III. Novelty and Impact:

- Incremental vs. Groundbreaking: Many ideas were criticized for being incrementally novel, building upon existing knowledge rather than proposing truly groundbreaking mechanisms.
- "Key Switch" Claims: Several ideas were criticized for prematurely claiming that the proposed mechanism is the "key switch" for ALS. This type of strong claim requires very strong supporting evidence. It is often better to frame the hypothesis in terms of a significant contributing factor and test its requirement for the pathology.
- Therapeutic Potential: While not always explicitly stated, the potential for translating the findings into therapeutic strategies is an important consideration. Ideas that offer a clear path toward therapeutic development may be viewed more favorably, provided a strong rationale is presented for the specific therapeutic approach.

IV. Assumptions and Validation:

- Strong Assumptions: Many hypotheses rely on strong assumptions that lack direct experimental support. Future ideas need to:
    - Clearly state all assumptions.
    - Provide a strong rationale for each assumption, citing relevant literature.
    - Propose experiments to directly test the most critical assumptions.
- Validation of Assumptions:
    - Reviewers consistently emphasized the need for rigorous experimental validation of all assumptions.

    - Assumptions should be addressed in the order of the proposed mechanism.
    - If the initial steps are not validated, it may be unnecessary to proceed to later steps.

V. General Advice Based on Common Critiques:

- Mechanistic Detail: Provide as much specific mechanistic detail as possible. Don't just state that pathway X is affected; describe how it is affected at the molecular level.
- Literature Review: Demonstrate a thorough understanding of the existing literature, including contradictory findings. Address how the proposed idea fits into the current understanding of ALS and how it differs from previous work.
- Focus on Causality: Always design experiments that can distinguish between cause and effect.
- Feasibility: Prioritize experiments that are feasible with available resources and expertise. Consider using a phased experimental approach. Prioritize the experiments used to validate assumptions in the order they appear in the proposed mechanism.
- Strong Rationale: Provide a clear and compelling rationale for every aspect of the hypothesis and experimental design.
- Specificity of Controls: Explain why the choices of controls used in the experimental design are the best choice to test the effects of interest. Demonstrate that the effect is not due to a more general effect or mechanism.
- Address Limitations: Acknowledge the limitations of the proposed approach and discuss potential alternative explanations.
- Quantitative data: Emphasize quantitative data, consider including mathematical modeling of data where appropriate.

10.11 Example of a research overview by the Meta-review agent

This is an example of a research overview generated by the Co-Scientist Meta-review agent for the research goal pertaining to explaining the basic mechanism of Amyotrophic Lateral Sclerosis (ALS). In addition to the individual hypotheses, this is the main output presented to the scientist by Co-Scientist.

[Main Research Directions]

We will be focusing on these interrelated areas:

- **Mitochondrial Dysfunction and Oxidative Stress:** This direction investigates defects in mitochondrial DNA repair, mitochondrial dynamics, calcium handling, and oxidative

stress response, focusing on their role in initiating inflammatory signaling and neurodegeneration.

- **RNA Processing and Stress Granules:** This direction explores abnormalities in RNA processing, transport, and modification, as well as impaired stress granule dynamics, and their impact on protein homeostasis and neuronal survival. It includes examining RNA-binding proteins, RNA structure and modifications, translation defects, and the interplay between RNA and proteostatic stress responses.
- **Protein Homeostasis and the Nucleocytoplasmic Transport:** This direction is aimed at investigating defects in protein folding, trafficking, aggregation, and degradation, as well as nucleocytoplasmic transport (NCT) dysfunction, with a particular focus on the nuclear pore complex (NPC). This includes how those pathways and processes influence, and are influenced by, TDP-43 and FUS protein function and localization.
- ...

Mitochondrial Dysfunction and Oxidative Stress

- Rationale: Mitochondrial dysfunction and oxidative stress are consistently implicated in ALS pathogenesis. Mitochondria are critical for cellular energy production, and their dysfunction can lead to a cascade of events including increased oxidative stress, impaired calcium handling, inflammation, and ultimately cell death. Given the high energy requirements of motor neurons, especially upper motor neurons, these deficits are expected to have a significant impact. We hypothesize that specific mitochondrial vulnerabilities, not just generalized dysfunction play a very early role in initiating disease and provide a specific cellular event which can be targeted for research and for potential future therapy.
- Recent Findings: Research has shown that mutations in mitochondrial DNA (mtDNA) maintenance genes are associated with ALS. Further there are links between impaired mitochondrial calcium handling and oxidative stress, with a particular focus on base excision repair (BER) pathway defects and a potential link to activation of the cGAS-STING pathway. These findings suggest that a deeper understanding of mtDNA integrity, repair mechanisms, and ROS dynamics is critical for uncovering the initial drivers of ALS.
- Areas of Research:
    - Mitochondrial DNA Repair Defects:
        - Why Research? Explore the possibility that a deficiency in base excision repair (BER) enzymes for mitochondrial DNA is a primary driver of ALS.
        - What to Research? Investigate the activity and expression of specific BER enzymes (e.g., OGG1) in iPSC-derived motor neurons from ALS patients and controls. Assess whether deficiencies correlate with increased accumulation of oxidized mtDNA lesions (e.g., 8-oxo-dG) and whether

        - these can be released into the cytoplasm after VDAC or MOMP activation. Determine if this release activates the cGAS-STING pathway.
        - Example Idea: Measure OGG1 enzyme activity, oxidized mtDNA levels (8-oxo-dG) and cGAS-STING activation in iPSC-derived motor neurons. Genetically manipulate (knock-out/overexpress) key components of the mitochondrial BER pathway or target specific pathways using small molecules. Use VDAC inhibitors to prevent release or modulate MOMP function to understand cause and effect of ox-mtDNA release and cGAS-STING activation on cell death and inflammatory outcomes.
    - Mitochondrial Calcium Handling and Oxidative Stress:
        - Why Research? Motor neurons have high calcium demands and are sensitive to oxidative damage. Imbalances in mitochondrial calcium handling and ROS production may trigger a cascade leading to neurodegeneration.
        - What to Research? Examine the role of mitochondrial calcium uniporter (MCU) and other calcium transporters in ALS. Assess if dysregulated calcium influx into mitochondria leads to increased ROS production and whether this is different in different types of motor neurons. Study the potential role of calcium handling defects as a primary trigger or modifier of the disease.
        - Example Idea: Use calcium indicators and ROS sensors in iPSC-derived motor neurons to monitor real-time calcium and ROS levels in mitochondria. Manipulate mitochondrial calcium uptake via pharmacological or genetic approaches (e.g., MCU inhibitors or knock-outs). Assess cell survival and the impact on inflammatory response markers and activation of the cGAS-STING pathway to assess direct causality.
    - …

RNA Processing and Stress Granules:

- ...

Protein Homeostasis and the Nucleocytoplasmic Transport:

- Rationale: The proper folding, trafficking, degradation, and localization of proteins are critical for cellular function. Defects in these processes can lead to the accumulation of toxic protein aggregates, disrupt cellular signaling, and initiate pathways leading to cell death in ALS. The nucleus is a key compartment, and transport into and out of this organelle is essential for cellular function.
- Recent Findings: Research has identified misfolded TDP-43 and FUS as key pathological features of ALS. Furthermore, the dysregulation of nucleocytoplasmic transport (NCT)

and the nuclear pore complex (NPC) is a recurring feature, with recent findings that emphasize defects in early stages of assembly and dynamic function of the NPC as an early initiating step.

- Areas of Research:
  - Ubiquitin-Proteasome System (UPS) Dysfunction:
    - Why Research? The UPS is a major cellular protein degradation machinery. Defects in the UPS can lead to an accumulation of misfolded proteins, which could lead to cellular stress and death. Exploring the potential of a functional deficiency in these system as an early trigger in disease.
    - What to Research: Investigate how UPS functionality is altered in ALS models, with particular attention to the role of E3 ligases in TDP-43 and FUS degradation. Determine whether specific modifications on ALS related proteins impair their degradation and whether specific pathways of protein quality control are affected in early stages of the disease. Assess if this is a primary cause or secondary outcome of other disease related pathways.
    - Example Idea: Use cell-based assays to measure UPS activity and substrate degradation rates in iPSC-derived motor neurons. Conduct proteomic analysis to identify specific ubiquitinated proteins that accumulate in ALS cells. Genetically manipulate UPS components or use small molecules to modulate their function and assess cellular responses (e.g. measuring protein aggregation and cell survival).
  - Autophagy Dysfunction:
    - Why Research? Autophagy is crucial for clearing cellular debris and aggregates. Dysfunctional autophagy can lead to the accumulation of toxic proteins and can lead to organelle defects.
    - What to Research? Explore how autophagy pathways are altered in ALS, focusing on the specific mechanisms of cargo recognition, autophagosome formation, and lysosome fusion. Assess if specific types of autophagy including mitophagy (mitochondrial specific) or lipophagy (lipid specific) are more vulnerable in specific cell types.
    - Example Idea: Track the formation and degradation of autophagosomes in real-time in hiPSC-derived motor neurons after different stress conditions. Manipulate key effectors of the autophagy pathway and observe cellular response, measuring protein aggregation, cell viability and inflammatory markers.
  - Nucleocytoplasmic Transport (NCT) Defects:
    - ...
  - Targeted Protein Misfolding:

        - …
- …

10.12 Example of research contact identification by the Meta-review agent

This is an example of a research contact identified by the Meta-review agent as a potential domain expert in the research topic and hypothesis of interest.

Research Direction: Oxidative DNA Damage & Mitochondrial Base Excision Repair (BER) in ALS

- [Researcher names]: They directly study the role of mitochondrial OGG1 (a key BER enzyme) in controlling cytosolic mtDNA release and neuroinflammation. Their expertise is highly valuable for understanding the link between BER, mtDNA, and inflammation. Also, they have experience with experiments using mtOGG1 overexpressing mice which is relevant to the in-vitro experiments proposed.
- …

---

11 An example of tool use in Co-Scientist with AlphaFold

Co-Scientist is a general purpose system broadly applicable across different areas of science and medicine. To better understand the capabilities and limitations of the system, we task it with the goal of suggesting protein sequences with specific properties. Determining the correct primary amino acid sequence with the desired properties is an essential part of protein engineering. While LLM-based systems can predict protein properties and suggest modifications [68], they can sometimes generate incorrect sequences (i.e., hallucinations). To address this, we integrate AlphaFold [69], a specialized AI system for predicting protein 3D structure, into our Co-Scientist. AlphaFold acts as a validation tool, evaluating the structural plausibility of sequences proposed by Co-Scientist and provides feedback. This increases the reliability of the sequence design optimization process, which can be further validated with wet laboratory experiments. The approach to integrate tools highlights how specialized AI models can work in collaboration with more general AI systems like Co-Scientist, facilitating the solution of complex challenges like protein design.

As an illustrative example, we used AlphaFold to assess a Co-Scientist's proposed modification to the OCT4 (octamer-binding transcription factor 4) protein (Supplementary Fig. 9), one of the four Yamanaka factors [70], to increase binding affinity of its DNA binding domain. Co-Scientist suggested adding a mechano-sensitive loop to the POU domain (a family of eukaryotic transcription factors) and a dynamic phosphorylation site outside of it. Co-Scientist first verified the proposed sequence against the UniProt database via web-search. AlphaFold then predicted the 3D structure of the modified protein, suggesting that the modifications maintained structural stability. These predictions were used to refine Co-Scientist's hypothesis, allowing it to improve its protein sequence design in subsequent iterations. We also independently validated the modification using ESM-2 [71], ESMFold [71], and RoseTTAFold [72]. ESM-2 predicted an increased log-likelihood ratio, ESMFold predicted a similar predicted local distance difference test (pLDDT), and RoseTTAFold predicted a similar confidence score (GDT), compared to the original sequence. The insertion and modification did not seem to disrupt SOX2 and OCT4 interactions, indicated by the similar pLDDT scores between the original and modified OCT4 sequences. However, this example is for demonstration purposes only. Further *in silico* analysis (e.g., predicting binding affinity and off-target effects), and thorough laboratory validation are necessary to confirm that the proposed modifications actually improve the complex roles of OCT4 binding, while maintaining SOX2 interaction integrity, during pluripotency.

Integrating AlphaFold within the Co-Scientist system offers a powerful approach for both improving existing proteins and designing entirely new ones. This integrated system allows researchers to iteratively optimize protein sequences for enhanced properties (e.g., stability, binding affinity, or catalytic activity) or putatively to create proteins with novel functions. It enables exploration of protein design while ensuring structural feasibility. Future work will focus on experimentally validating these capabilities and applying them to targeted protein design efforts as well as expansion to integration of other specialized AI tools with Co-Scientist.

12 Related works

Reasoning models and test-time compute scaling. The modern revolution in foundation AI models [73] and large language models (LLMs) has been largely driven by advances in pre-training techniques [74,75], leading to breakthroughs in models like the GPT and Gemini family [76,77]. These models, trained on increasingly massive internet-scale and multimodal datasets, have demonstrated impressive abilities in language understanding and generation leading to breakthrough performance in a variety of benchmarks [78,79]. However, a key area of ongoing development is enhancing their *reasoning* capabilities. This has led to the emergence of "reasoning models" which go beyond simply predicting the next word and instead attempt to mimic human thought processes [80]. One promising direction in this pursuit is the test-time compute paradigm. This approach moves beyond solely relying on the knowledge acquired during pre-training and allocates additional computational resources during inference to enable System-2 style thinking, which is a slower deliberate reasoning to reduce uncertainty and progress optimally towards the goal [81]. This concept emerged with early successes such as AlphaGo [82], which used Monte Carlo Tree Search (MCTS) to explore game states and strategically select moves, and Libratus [83], which employed similar techniques to achieve superhuman performance in poker. This paradigm has now found applications in LLMs, where increased compute at test-time allows for more thorough exploration of possible responses, leading to improved reasoning and accuracy [80,84–90]. Recent advancements, like the Deepseek-R1 model [91], further demonstrate the potential of test-time compute by leveraging reinforcement learning to refine the model's "chain-of-thought" and enhance complex reasoning abilities over longer horizons. In this work, we propose a significant scaling of the test-time compute paradigm using inductive biases derived from the scientific method to design a multi-agent framework for scientific reasoning and hypothesis generation without any additional learning techniques.

AI-driven scientific discovery. AI-driven scientific discovery represents a paradigm shift in how research is conducted across various scientific domains. Recent advancements, particularly the development of large deep learning and generative models, have cemented AI's role in scientific discovery. This is best exemplified by AlphaFold 2's remarkable progress in the grand challenge of protein structure prediction, which has revolutionized structural biology and opened new avenues for drug discovery and materials science [92]. Other notable examples include the development of novel antibiotics, protein binder design, and material discovery with AI [93–95].

Building on these successes with specialized, bespoke AI models, there has been recent work exploring the even more ambitious goal of fully integrating AI, especially modern LLM-based systems, into the complete research workflow, from initial hypothesis generation all the way to manuscript writing. This end-to-end integration represents a significant shift, presenting both unprecedented opportunities and significant challenges as the field moves beyond specialized AI tools toward realizing the potential of AI as an active collaborator, or even, as some envision, a nascent "AI scientist" [96,97].

As an example of this shift, Liang et al. [98] directly assessed the utility of LLMs for providing feedback on research manuscripts. Through both a retrospective analysis of existing peer reviews and a prospective user study, they demonstrated the significant concordance between LLM-generated feedback and that of human reviewers. Their study, using GPT-4 [77], found that a majority of researchers perceived LLM-generated feedback as helpful, and in some instances, even more beneficial than feedback from human colleagues. However, while valuable, their work focuses solely on the feedback stage of the scientific process, leaving open the question of how LLMs might be integrated into the full research cycle, from hypothesis formation to experimental validation and manuscript writing.

Another effort embodying this shift is PaperQA2 [99], an AI agent for scientific literature search and summarization. The authors claimed to surpass PhD and postdoc researchers on multiple literature research tasks, as measured both by performance on objective benchmarks and human evaluations. While the system is useful for synthesizing information, it does not engage in scientific reasoning for novel hypothesis generation.

HypoGeniC, a system proposed by Zhou et al. [100], tackles hypothesis generation by iteratively refining hypotheses using LLMs and a multi-armed bandit-inspired approach. The process begins with a small set of examples, from which initial hypotheses are generated. These hypotheses are then iteratively updated through exploration and exploitation, guided by a reward function based on training accuracy. This refined set of hypotheses is subsequently used to construct an interpretable classifier. However, the method's reliance on retrospective data for evaluation means the degree to which the system can generate truly novel hypotheses remains an open question. Furthermore, the system lacks end-to-end validation beyond subjective human evaluations.

Ifargan et al. [101] present “data-to-paper”, a platform that systematically guides multiple LLM and rule-based agents to generate research papers, with automated feedback mechanisms and information tracing for verification. However, the evaluations are limited to recapitulating existing peer-reviewed publications and it is unclear if the system can generate truly novel, yet grounded hypotheses and research proposals.

Virtual Lab [102] is another closely related work. Here, the authors propose a team of LLM agents with a “principal investigator” LLM guiding a team of specialized LLM agents to solve a scientific problem. The LLM team receives high level human supervision. The authors demonstrate the utility of their work by leveraging Virtual Lab to design nanobody binders to recent variants of SARS-CoV-2 with experimental validation. While similar in spirit, there are significant design differences to our approach and the generality of the system remains unclear.

Boiko et al. [103] introduced “Coscientist”, a multi-agent system powered by GPT-4, designed for autonomous execution of complex chemical experiments. This system integrates capabilities such as web and document searching, and code execution, to facilitate independent experimental design, planning, and execution. In addition to similar sounding names, both “Coscientist” and our system share the overarching goal of accelerating scientific discovery through AI. However, there are several important distinctions. Notably, “Coscientist” is quite

narrowly focused on chemical research while ours is much broadly applicable across science. Secondly, our system has important technical innovations that lead to a self-improving system that can uncover new, original knowledge while their approach is a more vanilla-stitching of GPT-4 based agents. Finally, despite the name, "Coscientist" prioritizes a high degree of autonomy in experimental execution, directly interfacing with laboratory hardware. Our system, instead, is explicitly designed as a collaborative tool, emphasizing a "scientist-in-the-loop" approach and centers on the more cognitive aspects of the research process.

Finally, Lu et al. [96] propose "The AI Scientist", a fully automated system designed to conduct research using multiple collaborating LLM agents. These agents handle all stages of the research process, from defining research problems and conducting literature reviews to designing and executing experiments, and even writing up the results. The design shares similarities with our work. The key differences being our focus on the scaling of the test-time compute paradigm to generate high quality hypotheses and research proposals. Secondly, their proposed system has limited automated evaluations; in contrast, our work has a combination of automated, human expert and end-to-end wet lab validations. Finally, our goal is to not to automate scientific discovery, rather to build a helpful AI collaborator for scientists.

AI for biomedicine. More broadly, large AI models are increasingly demonstrating their potential in biomedical science. Both general purpose (GPT-4, Gemini) and specialized LLMs (Med-PaLM, Med-Gemini, Galactica, Tx-LLM) have shown strong performance on biomedical reasoning and question-answering benchmarks [76,77,104–107]. Beyond benchmarks, Med-PaLM 2, was successfully applied to identify causative murine genetic factors for traits such as diabetes, cataracts, and hearing loss [108], which is an early example of hypothesis generation and LLM-assisted discovery. We have also seen the exciting development of specialized foundation and large language models trained on DNA, RNA and protein sequences with a variety of applications [109–112]. Although AI in biology and medicine often necessitates specialization, the rapid progress of frontier AI models has blurred the distinction. As these models grow in scale, data diversity, and complexity, they continue to achieve breakthroughs in areas once thought to require domain-specific AI. Our Co-Scientist, with its modular multi-agent architecture, is flexibly designed to build on top of these advancements in general-purpose frontier AI models and leverage specialized AI models as tools to enhance the capabilities.

Drug repurposing is an important area of validation experiments in this work. The traditional approach to this task requires both computational and experimental approaches and a comprehensive understanding of disease-drug interactions [113,114]. While methods like knowledge graphs with graph convolutional networks have shown promise [115,116], their applicability is limited by the initial knowledge graph's scope. TxGNN [117], an example of a specialized biomedical foundation model with a graph based approach, addresses "zero-shot" repurposing for novel diseases but remains dependent on the underlying knowledge graph's quality and lacks sufficient scalability and explainability. Furthermore, no end-to-end validations of the model predictions were reported in the study. In contrast, our work, leveraging state-of-the-art LLMs in

the Co-Scientist setup is more scalable. We report a combination of expert evaluations and wet-lab experiments to validate the system predictions.

Supplementary figures

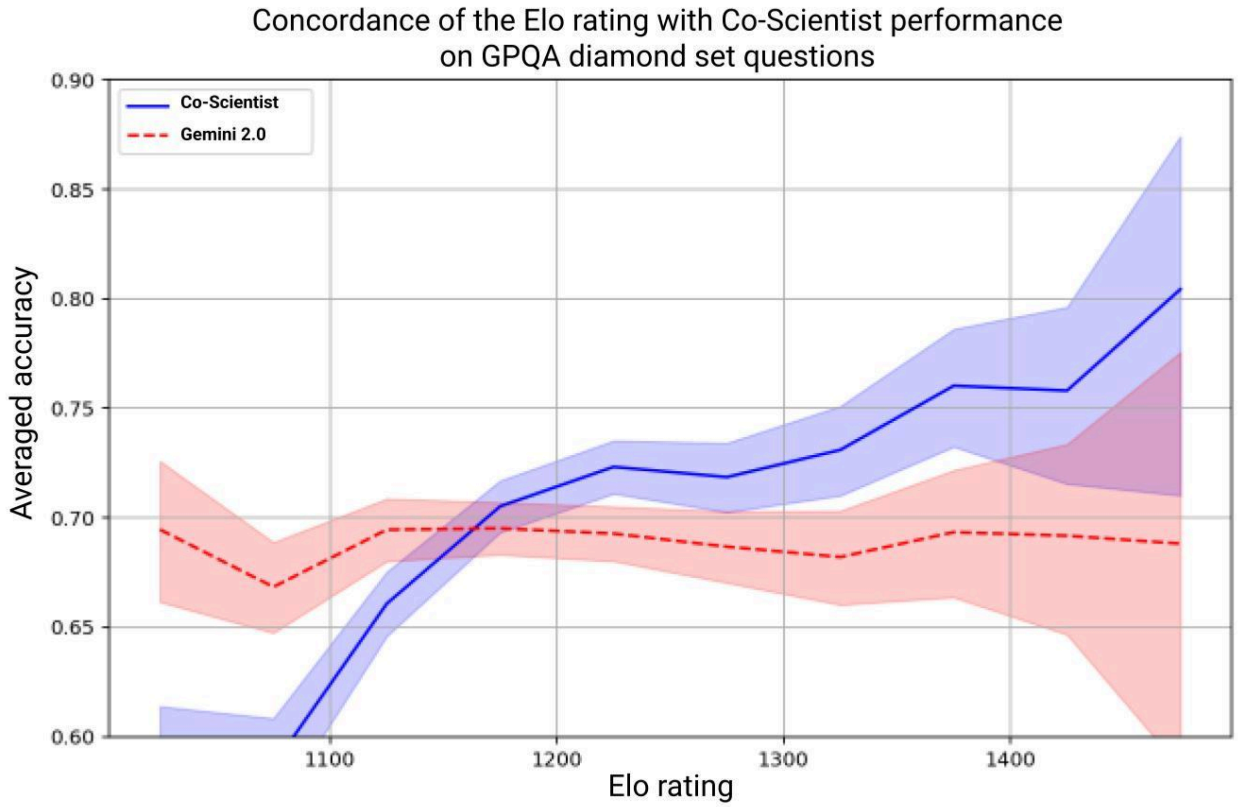

Supplementary Fig. 1 | Concordance of the auto-evaluation Elo metric with Co-Scientist performance on GPQA. The blue line in the figure shows the average accuracy of Co-Scientist responses, grouped by their Elo rating. The red line indicates the average accuracy of the corresponding reference Gemini 2.0 responses to the same set of GPQA questions, grouped by Elo rating. Note that the Elo metric is auto-evaluated and not based on the ground truth. The shaded area indicates the corresponding 95% confidence interval.

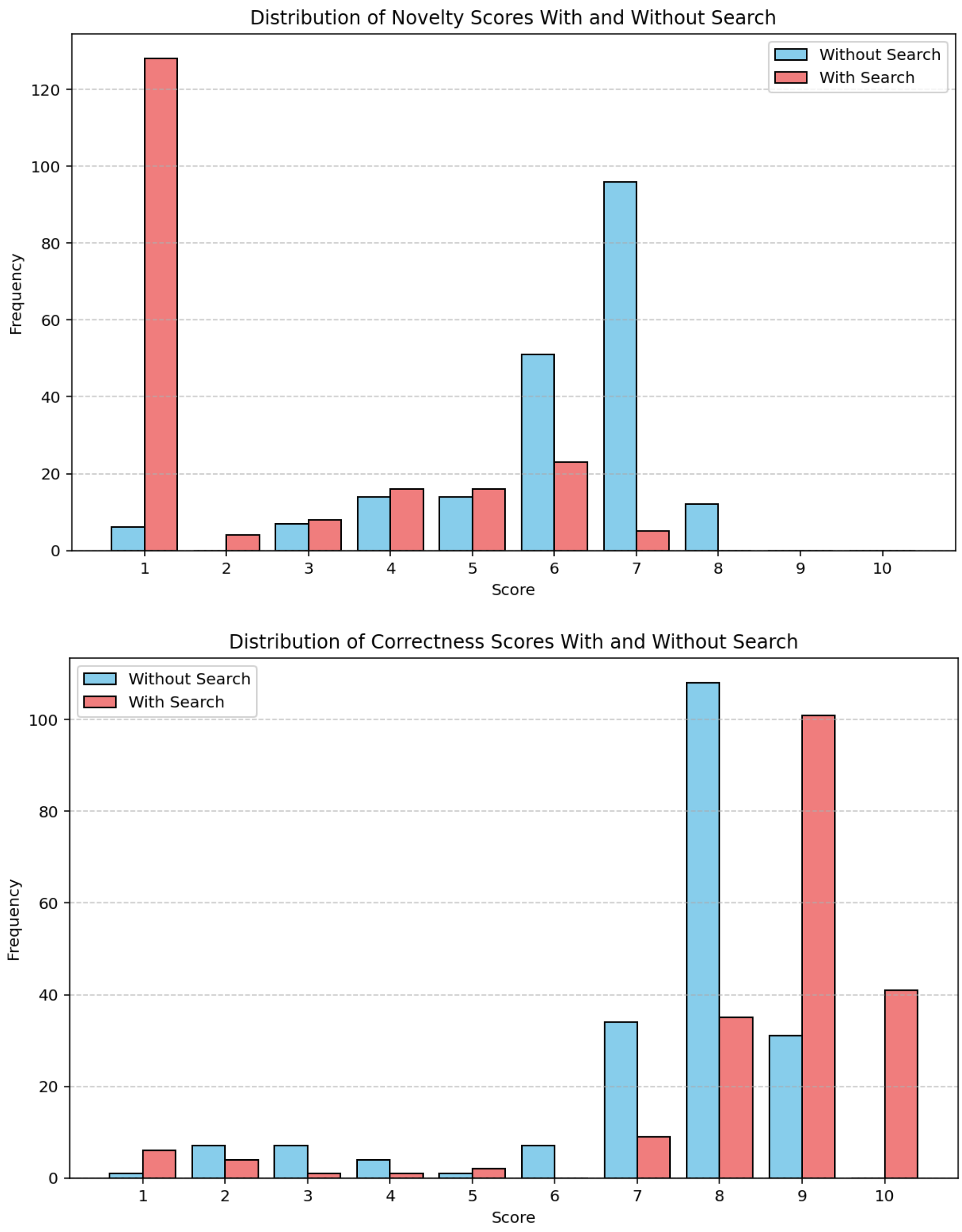

Supplementary Fig. 2 | Distribution of novelty and correctness scores with and without external search. With external search tool use, the novelty score (upper) significantly drops and the correctness score (lower) increases, which indicates that search is critical for grounding the model and preventing the hallucination of novelty.

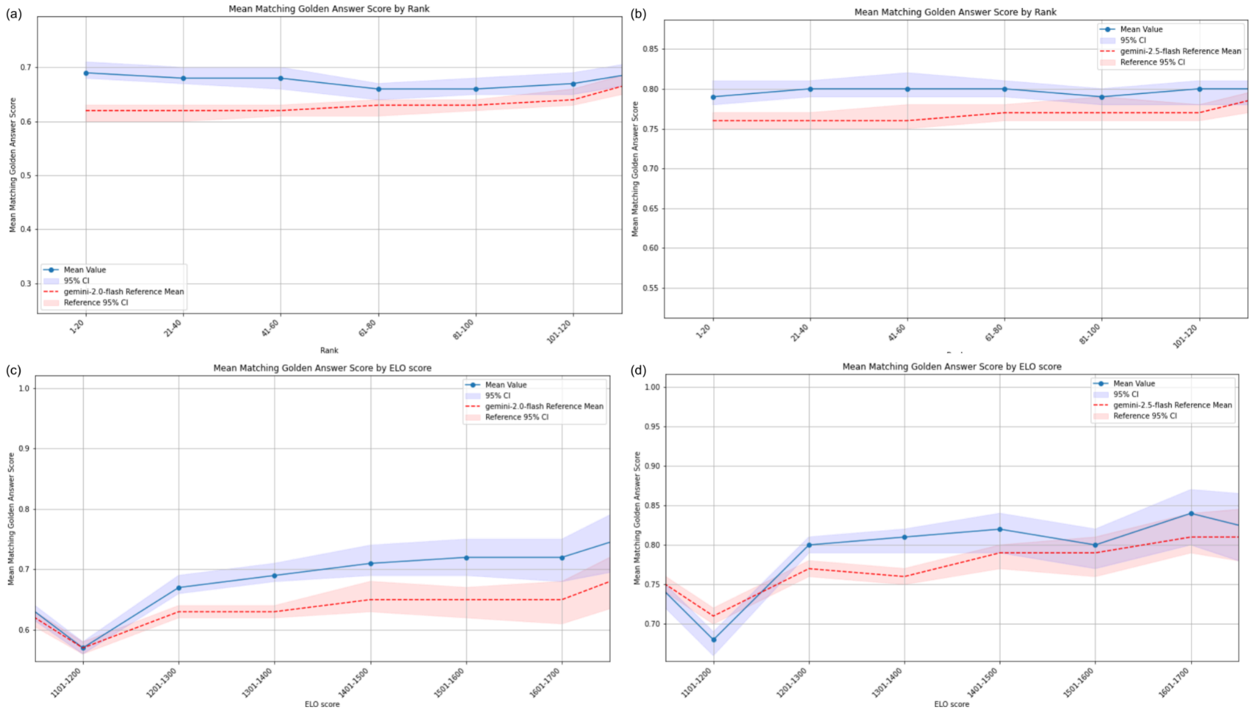

Supplementary Fig. 3 | Idea ranking and Elo rating versus average accuracy, using Gemini Flash 2.0 and 2.5. (a) Idea ranking versus accuracy, Gemini Flash 2.0. (b) Idea ranking versus accuracy, Gemini Flash 2.5. (c) Idea Elo rating versus accuracy, Gemini Flash 2.0. (d) Idea Elo rating versus accuracy, Gemini Flash 2.5.

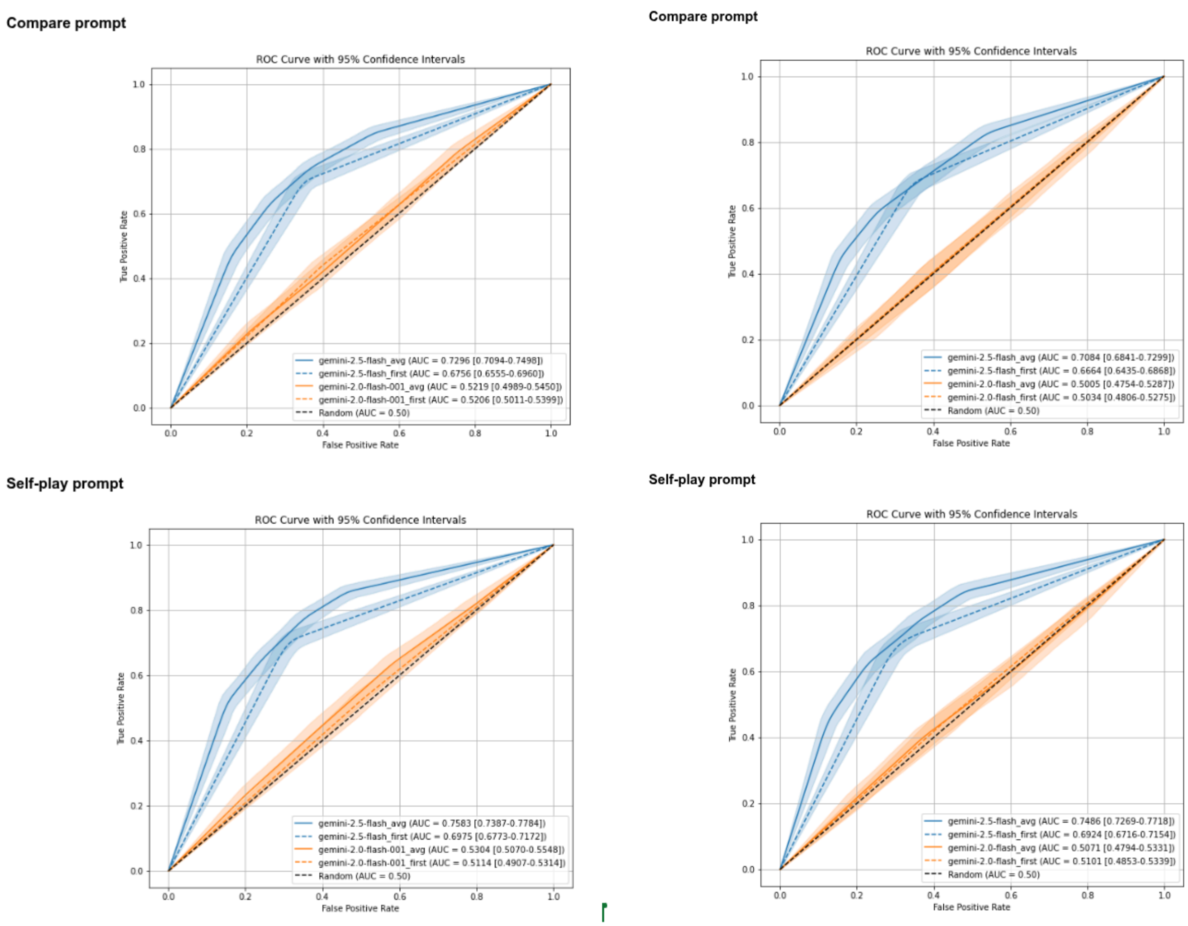

Supplementary Fig. 4 | The comparison between the Ranking agent using the simple comparison prompt versus the self-play debate prompt. (avg: AUC computed using the average 4 match predictions, 2 in AB and 2 in BA; first: AUC computed using the first of 4 predictions)

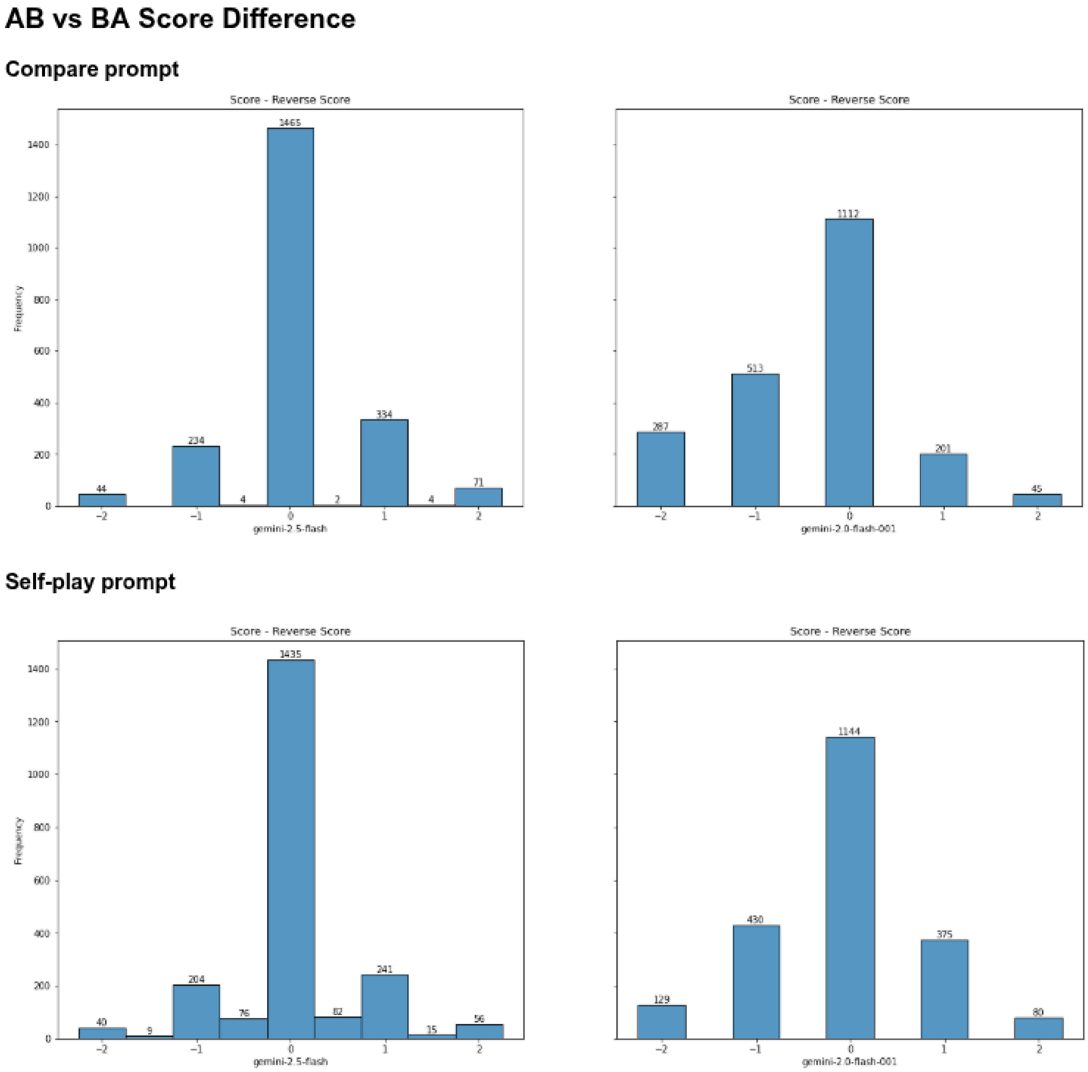

Supplementary Fig. 5 | The comparison between swapping the position. We found the self-play debate prompt has stronger robustness than the simple comparison prompt regarding the position bias.

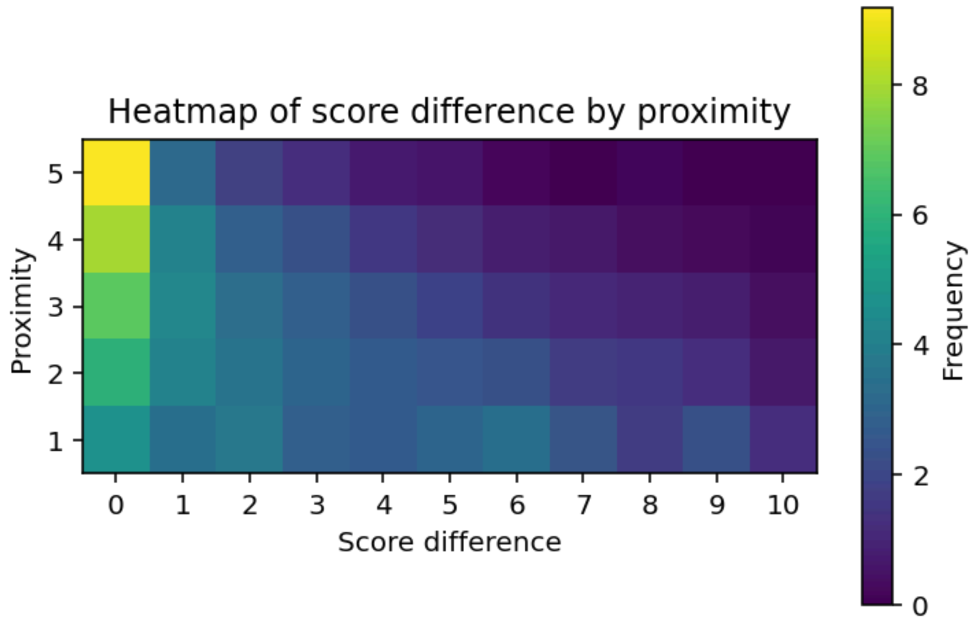

Supplementary Fig. 6 | Idea proximity computed by Proximity agent versus the score difference.

(a)

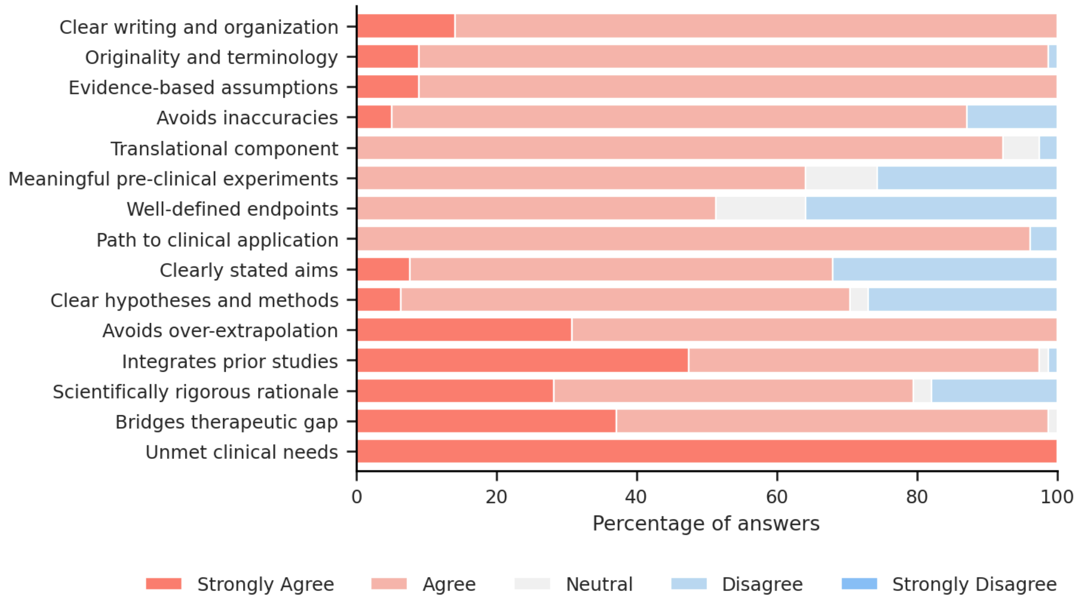

(b)

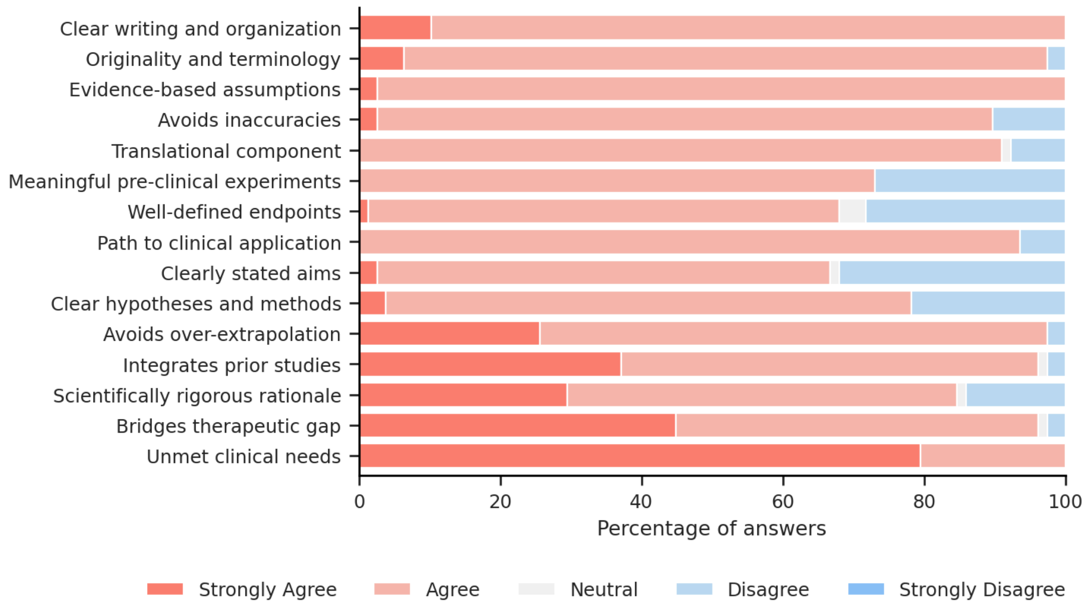

Supplementary Fig. 7 | Clinical expert evaluation for Co-Scientist generated drug repurposing hypotheses in the NIH Specific Aims Page format. Two batches of expert hematologists and oncologists independently reviewed the same 78 drug repurposing research proposals, which Co-Scientist had formatted as NIH Specific Aims Pages. The evaluation followed an adapted NIH grant proposal evaluation rubric, detailed in the Supplementary Note 4.3. Overall, two groups of experts judged the Specific Aims proposals from Co-Scientist to be of high quality across all axes of the rubric. (a) and (b) represent two separate groups of expert evaluation results.

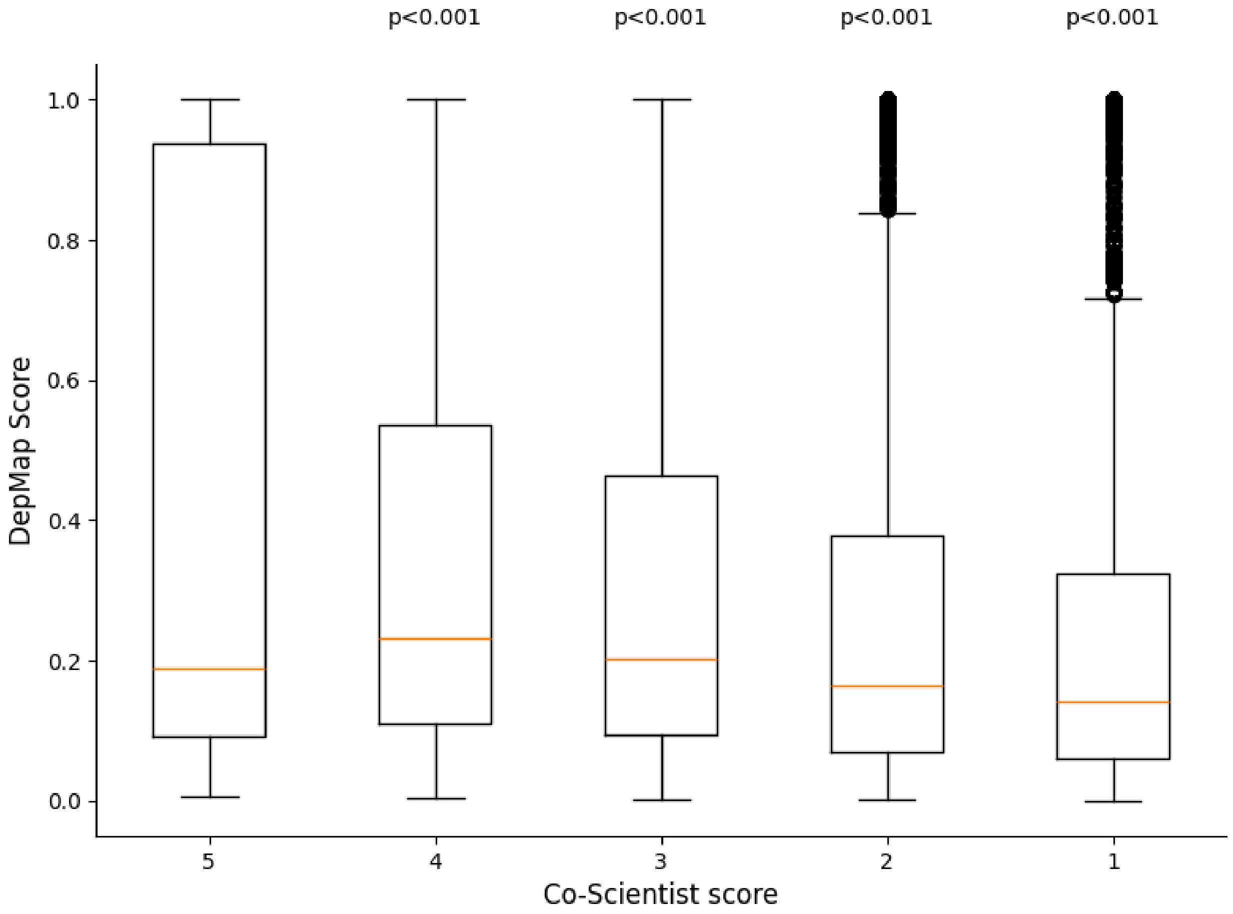

Supplementary Fig. 8 | Co-Scientist review score is concordant with the DepMap score. We demonstrate the distribution of the DepMap score across five Co-Scientist review score groups. Groups with higher Co-Scientist scores tended to have higher DepMap scores. Box plots show the median (center line), first and third quartiles (box limits), and 1.5 times the interquartile range (whiskers). All pairwise comparisons between the group of Co-Scientist score of 5 and each of the other groups are statistically significant (two-sided Wilcoxon rank-sum test without multiple comparison adjustments; exact *P* values: score 5 vs 4, $P = 2.423 \times 10^{-9}$; score 5 vs 3, $P < 10^{-10}$; score 5 vs 2, $P < 10^{-10}$; score 5 vs 1, $P < 10^{-10}$). Sample sizes are n = 202 (score 5), n = 1576 (score 4), n = 6473 (score 3), n = 17404 (score 2), n = 15041 (score 1) drug-cancer pairs respectively.

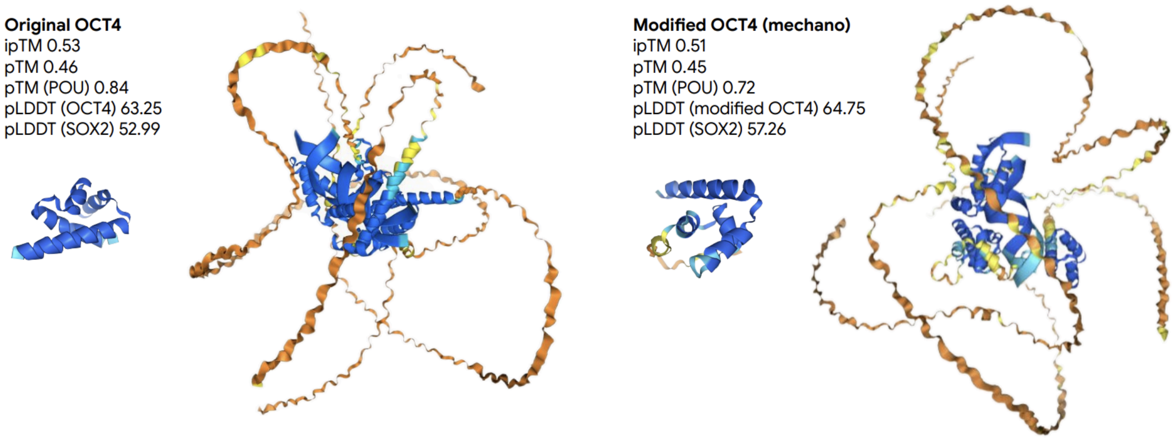

Supplementary Fig. 9 | AlphaFold predicted protein 3D structure and metrics for original OCT4 and Co-Scientist suggested modifications. (left panel) original OCT4 sequence with SOX2 and DNA binding (right panel) modified OCT4 sequence with SOX2 and DNA binding. The left 3D structure in each panel is the POU domain of the corresponding OCT4 sequence. The predicted template modeling (pTM) score, the interface predicted template modeling (ipTM), and predicted local distance difference test (pLDDT) are derived from the AlphaFold outputs.

Supplementary tables

Supplementary Table 1 | Summary of the ablation analysis.

Agent	Ablation	Key findings	Metrics
Generation Agent	Analyzed the contribution of different generation strategies to correct hypotheses.	A diverse set of generation strategies is critical for producing correct hypotheses, with different strategies proving more effective depending on the task.	Contribution scores highlight top strategies per benchmark.
Reflection agent with external search tool use	Compared novelty and correctness reviews with (full, with search) and without (initial, no search) access to the search tool, using a dataset of published (non-novel, correct) ideas.	Access to external search is critical for accurate novelty assessment and significantly improves correctness evaluation. It grounds the model, preventing it from incorrectly rating existing ideas as novel.	On the rediscovery benchmark, search improved novelty scores from 6.14 (incorrectly novel) to 2.38 (correctly non-novel) and correctness scores from 7.4 to 8.46. AUC for predicting correctness on GPQA also increased from 0.643 to 0.651 (Flash 2.0).
Ranking agent prompting strategy	Compared the simple comparison prompt vs. the scientific debate prompt for pairwise idea comparison.	The scientific debate prompt is significantly more accurate for comparing high-quality ideas.	On top-ranked ideas, AUC for correctly identifying the better idea was noticeably higher for the scientific debate prompt.
Ranking agent bias	Assessed score stability by comparing A vs. B and B vs. A matches for both prompt types.	The scientific debate prompt significantly reduces positional bias (second player advantage), leading to more stable and reliable evaluations.	Histograms of score differences show a highly symmetric distribution around zero for the scientific debate prompt compared to the biased basic prompt.

Evolution Agent	Compared top-ranked baseline hypotheses vs. top-ranked improved hypotheses.	The iterative refinement process significantly boosts the quality and correctness of the final hypotheses.	On GPQA, precision increased from 70.9% to 75.4%. On the constructed dataset, average hypothesis quality score increased from 4.7 to 5.6.
Meta-review Agent	Compared correctness reviews with (full+recurrent) or without (full) meta-review guidance.	The meta-review agent consistently improves the quality of correctness reviews.	On the constructed dataset, AUC for predicting solution correctness increased from 0.521 to 0.597. On GPQA, AUC increased from 0.629 to 0.634 (Flash 2.5).
Proximity Agent	Correlated the agent's semantic proximity scores with the ground-truth quality score differences of hypothesis pairs.	The proximity score accurately reflects the semantic similarity of hypotheses, enabling effective clustering to ensure a diverse exploration of the problem space.	Strong correlation between high proximity scores (4-5) and low quality score differences, and vice versa.

Supplementary Table 2 | Count distribution of the cancer types in the Specific Aims drug repurposing proposals generated by Co-Scientist.

Repurposed Cancer	Count
Acute Myeloid Leukemia (LAML)	13
Colon adenocarcinoma (COAD)	10
Breast invasive carcinoma (BRCA)	10
Skin Cutaneous Melanoma (SKCM)	8
Lung adenocarcinoma (LUAD)	6
Head and Neck squamous cell carcinoma (HNSC)	5
Bladder Urothelial Carcinoma (BLCA)	5
Pancreatic adenocarcinoma (PAAD)	4
Stomach adenocarcinoma (STAD)	3
Rectum adenocarcinoma (READ)	3
Esophageal carcinoma (ESCA)	3
Uveal Melanoma (UVM)	2
Uterine Corpus Endometrial Carcinoma (UCEC)	2
Prostate adenocarcinoma (PRAD)	2
Lung squamous cell carcinoma (LUSC)	1
Cervical squamous cell carcinoma and endocervical adenocarcinoma (CESC)	1
Grand Total	78

Supplementary Table 3 | TCGA cancer type. We consider all cancer types except CNTL, FPPP, and MISC while exploring the drug repurposing candidates with preclinical evidence. *Not a cancer type.

Cancer Name Abbreviation	Cancer Name
LAML	Acute myeloid leukemia
ACC	Adrenocortical carcinoma
BLCA	Bladder urothelial carcinoma
LGG	Brain lower grade glioma
BRCA	Breast invasive carcinoma
CESC	Cervical squamous cell carcinoma and endocervical adenocarcinoma
CHOL	Cholangiocarcinoma
LCML	Chronic myelogenous leukemia
COAD	Colon adenocarcinoma
CNTL*	Controls
ESCA	Esophageal carcinoma
FPPP*	FFPE Pilot Phase II
GBM	Glioblastoma multiforme
HNSC	Head and neck squamous cell carcinoma
KICH	Kidney chromophobe
KIRC	Kidney renal clear cell carcinoma
KIRP	Kidney renal papillary cell carcinoma
LIHC	Liver hepatocellular carcinoma
LUAD	Lung adenocarcinoma
LUSC	Lung squamous cell carcinoma
DLBC	Lymphoid neoplasm diffuse large B-cell lymphoma
MESO	Mesothelioma
MISC*	Miscellaneous
OV	Ovarian serous cystadenocarcinoma
PAAD	Pancreatic adenocarcinoma
PCPG	Pheochromocytoma and paraganglioma
PRAD	Prostate adenocarcinoma
READ	Rectum adenocarcinoma
SARC	Sarcoma

SKCM	Skin cutaneous melanoma
STAD	Stomach adenocarcinoma
TGCT	Testicular germ cell tumors
THYM	Thymoma
THCA	Thyroid carcinoma
UCS	Uterine carcinosarcoma
UCEC	Uterine corpus endometrial carcinoma
UVM	Uveal melanoma

Supplementary Table 4 | Co-Scientist curated list of human AML cell lines for AML drug repurposing studies. The list below details Co-Scientist recommended AML cell lines, the specific AML subtype reflected by each cell-line, key molecular features, and their specific utility in drug repurposing studies. MOLM-13, HL-60, and NOMO-1, representing three AML subtypes, are used in the study (boldfaced).

**Cell Line Name**	**AML Subtype (FAB/WHO Classification)**	**Key Genetic Mutations**	**Justification for Use in Drug Repurposing**
MV4-11	AML with t(4;11)(q21;q23); KMT2A-AFF1	FLT3-ITD, KMT2A (MLL) rearrangement (t(4;11)). Some studies have detected low-frequency TP53 mutations.	Represents a high-risk AML subtype. The presence of FLT3-ITD makes it an essential model for testing FLT3 inhibitors and overcoming resistance. The KMT2A rearrangement also provides a target for the novel menin inhibitors.
**MOLM-13**	**AML with t(9;11)(p22;q23); KMT2A-MLLT3**	**FLT3-ITD, KMT2A (MLL) rearrangement (t(9;11)), possible RAS mutations.**	**Similar to MV4-11, it is a key model for FLT3-ITD targeted therapies. The different KMT2A translocation compared to other lines allows for broader screening of epigenetic modifiers.**
OCI-AML3	AML with myelodysplasia-related changes (AML-M4)	NPM1 (Type A) mutation, DNMT3A (R882C) mutation.	Represents one of the most common genetic subtypes of AML. The NPM1 mutation makes it a crucial model for testing therapies targeting nuclear-cytoplasmic transport and other associated pathways. The DNMT3A mutation allows for the screening of epigenetic modifiers.
**HL-60**	**Originally classified as M3, but lacks the characteristic t(15;17) and is now considered AML M2.**	**TP53 deletion, NRAS (Q61L) mutation, MYC amplification.**	**A classic AML cell line used for studying myeloid differentiation. Its TP53 deficient status makes it valuable for testing drugs that are effective in p53-null cancers. The NRAS mutation provides a target for signaling pathway inhibitors.**

OCI-AML2	AML with myelodysplasia-related changes (AML M4)	DNMT3A (R635W) mutation. Some reports indicate low-frequency TP53 mutations.	As a cell line with a DNMT3A mutation but wild-type NPM1 and FLT3, it serves as an excellent control to dissect the specific effects of DNMT3A-targeted drugs.
THP-1	Acute monocytic leukemia (AML M5a)	KMT2A (MLL) rearrangement (t(9;11)), TP53 and PTEN mutations/deletions.	Represents monocytic leukemia and is a good model for studying MLL-rearranged AML. The presence of TP53 and PTEN alterations makes it suitable for testing drugs that target these pathways or exploit synthetic lethality.
Kasumi-1	AML with t(8;21)(q22;q22); RUNX1-RUNX1T1	RUNX1-RUNX1T1 (AML1-ETO) fusion, c-Kit (N822K) mutation, TP53 (R248Q) mutation.	A key model for core-binding factor (CBF) AML. The RUNX1-RUNX1T1 fusion is a target for epigenetic drugs, and the activating c-Kit mutation makes it ideal for testing tyrosine kinase inhibitors.
**NOMO-1**	**Acute monocytic leukemia (AML M5a)**	**KMT2A (MLL) rearrangement (t(9;11)), KRAS (G13D), and TP53 mutations.**	**Another important model for MLL-rearranged AML, specifically of the monocytic subtype. The co-occurrence of KRAS and TP53 mutations with the MLL rearrangement allows for the study of drugs targeting multiple oncogenic pathways.**
EOL-1	Eosinophilic leukemia	FIP1L1-PDGFRA fusion, KMT2A (MLL) partial tandem duplication.	Represents a rare subtype of myeloid leukemia characterized by eosinophilia. The FIP1L1-PDGFRA fusion makes it highly sensitive to tyrosine kinase inhibitors like imatinib, serving as a positive control and a model for studying resistance mechanisms.

Supplementary references

1. Rein, D. *et al.* GPQA: A graduate-level Google-proof Q&A benchmark. in *First Conference on Language Modeling* (2024).
2. Sexauer, A. N. *et al.* DHODH: a promising target in the treatment of T-cell acute lymphoblastic leukemia. *Blood Adv* **7**, 6685–6701 (2023).
3. Broad. DepMap. *DepMap Public 24Q2* (2024).
4. Arafeh, R., Shibue, T., Dempster, J. M., Hahn, W. C. & Vazquez, F. The present and future of the Cancer Dependency Map. *Nat. Rev. Cancer* **25**, 59–73 (2025).
5. van Herpen, C. M. L. *et al.* Biomarker results from a phase II study of MEK1/2 inhibitor binimetinib (MEK162) in patients with advanced *NRAS*- or *BRAF*-mutated melanoma. *Oncotarget* **10**, 1850–1859 (2019).
6. Ball, B. J. *et al.* The prognosis and durable clearance of RAS mutations in patients with acute myeloid leukemia receiving induction chemotherapy. *Am J Hematol* **96**, E171–E175 (2021).
7. Verstovsek, S. & Komrokji, R. S. A comprehensive review of pacritinib in myelofibrosis. *Future Oncol* **11**, 2819–2830 (2015).
8. Perrone, S., Ottone, T., Zhdanovskaya, N. & Molica, M. How acute myeloid leukemia (AML) escapes from FMS-related tyrosine kinase 3 (FLT3) inhibitors? Still an overrated complication? *Cancer Drug Resist* **6**, 223–238 (2023).
9. Bosman, M. C. J., Schuringa, J. J. & Vellenga, E. Constitutive NF-κB activation in AML: Causes and treatment strategies. *Crit Rev Oncol Hematol* **98**, 35–44 (2016).
10. Hou, S. *et al.* PHF6 maintains acute myeloid leukemia via regulating NF-κB signaling pathway. *Leukemia* **37**, 1626–1637 (2023).

11. Banker, D. E. *et al.* Cholesterol synthesis and import contribute to protective cholesterol increments in acute myeloid leukemia cells. *Blood* **104**, 1816–1824 (2004).

12. Brehmer, D. *et al.* Discovery and Pharmacological Characterization of JNJ-64619178, a Novel Small-Molecule Inhibitor of PRMT5 with Potent Antitumor Activity. *Mol Cancer Ther* **20**, 2317–2328 (2021).

13. Haque, T. *et al.* Phase 1 study of JNJ-64619178, a protein arginine methyltransferase 5 inhibitor, in patients with lower-risk myelodysplastic syndromes. *Leuk Res* **134**, 107390 (2023).

14. Ramaswami, R. *et al.* A phase I/II study of abemaciclib, a CDK4/6 inhibitor, in participants with HIV-associated and HIV-negative Kaposi sarcoma. *J. Clin. Oncol.* **43**, 11505–11505 (2025).

15. Sebaugh, J. L. Guidelines for accurate EC50/IC50 estimation. *Pharm Stat* **10**, 128–134 (2011).

16. Kampen, K. R. *et al.* Insights in dynamic kinome reprogramming as a consequence of MEK inhibition in MLL-rearranged AML. *Leukemia* **28**, 589–599 (2014).

17. Scheidegger, N. *et al.* Combining menin and MEK inhibition to target poor prognostic KMT2A-rearranged RAS pathway-mutant acute leukemia. *Blood* **142**, 166 (2023).

18. Shrader-Frechette, K. S. *Ethics of Scientific Research*. (Rowman & Littlefield Publishers, 1994).

19. Resnik, D. B. *The Ethics of Science: An Introduction*. (Routledge, 2005).

20. Rollin, B. E. *Science and Ethics*. (Cambridge University Press, 2006).

21. Alasuutari, P., Bickman, L. & Brannen, J. *The SAGE Handbook of Social Research Methods*. (SAGE, 2008).

22. Menapace, M. Scientific Ethics: A New Approach. *Sci Eng Ethics* **25**, 1193–1216 (2019).

23. Edel, A. *Science and the Structure of Ethics*. (Routledge, 2018).

24. Miller, S. & Selgelid, M. J. Ethical and philosophical consideration of the dual-use dilemma in the biological sciences. *Sci Eng Ethics* **13**, 523–580 (2007).

25. Selgelid, M. J. Governance of dual-use research: an ethical dilemma. *Bull World Health Organ* **87**, 720–723 (2009).

26. Pustovit, S. V. & Williams, E. D. Philosophical aspects of dual use technologies. *Sci Eng Ethics* **16**, 17–31 (2010).

27. Forge, J. A note on the definition of 'dual use'. *Sci Eng Ethics* **16**, 111–118 (2010).

28. Kuhlau, F., Höglund, A. T., Eriksson, S. & Evers, K. The ethics of disseminating dual-use knowledge. *Research Ethics* **9**, 6–19 (2013).

29. Shaw, S. & Barrett, G. Research governance: regulating risk and reducing harm? *J R Soc Med* **99**, 14–19 (2006).

30. Rothstein, H., Irving, P., Walden, T. & Yearsley, R. The risks of risk-based regulation: insights from the environmental policy domain. *Environ Int* **32**, 1056–1065 (2006).

31. Ludlow, K., Bowman, D. M., Gatof, J. & Bennett, M. G. Regulating emerging and future technologies in the present. *NanoEthics* **9**, 151–163 (2015).

32. Verschraegen, G. Regulating scientific research: A constitutional moment? *Journal of Law and Society* **45**, S163–S184 (2018).

33. Jobin, A., Ienca, M. & Vayena, E. The global landscape of AI ethics guidelines. *Nature machine intelligence* **1**, 389–399 (2019).

34. Wansley, M. T. Regulation of emerging risks. *Vand. L. Rev.* **69**, 401 (2016).

35. Gabriel, I. *et al.* The ethics of advanced AI assistants. *arXiv preprint arXiv:2404.16244*

(2024).

36. Tang, X. *et al.* Prioritizing safeguarding over autonomy: Risks of LLM agents for science. in *ICLR 2024 Workshop on Large Language Model (LLM) Agents* (2024).

37. Shevlane, T. *et al.* Model evaluation for extreme risks. *arXiv preprint arXiv:2305.15324* (2023).

38. Bova, P., Di Stefano, A. & Han, T. A. Quantifying detection rates for dangerous capabilities: a theoretical model of dangerous capability evaluations. *arXiv preprint arXiv:2412.15433* (2024).

39. Phuong, M. *et al.* Evaluating frontier models for dangerous capabilities. *arXiv preprint arXiv:2403.13793* (2024).

40. Sabour, S. *et al.* Human decision-making is susceptible to AI-driven manipulation. *arXiv preprint arXiv:2502.07663* (2025).

41. Ke, P. *et al.* CritiqueLLM: Towards an informative critique generation model for evaluation of large language model generation. in *Proceedings of the 62nd Annual Meeting of the Association for Computational Linguistics (Volume 1: Long Papers)* 13034–13054 (2024).

42. Vu, T. *et al.* Foundational autoraters: Taming large language models for better automatic evaluation. *arXiv preprint arXiv:2407.10817* (2024).

43. Wei, H. *et al.* Systematic evaluation of LLM-as-a-judge in LLM alignment tasks: Explainable metrics and diverse prompt templates. *arXiv preprint arXiv:2408.13006* (2024).

44. Lan, T. *et al.* CriticEval: Evaluating large language model as critic. *arXiv preprint arXiv:2402.13764* (2024).

45. Zheng, L. *et al.* Judging LLM-as-a-judge with mt-bench and chatbot arena. *Advances in neural information processing systems* **36**, 46595–46623 (2023).

46. Li, D. *et al.* From generation to judgment: Opportunities and challenges of LLM-as-a-judge. in *Proceedings of the 2025 Conference on Empirical Methods in Natural Language Processing* 2757–2791 (2025).

47. Gou, Z. *et al.* Critic: Large language models can self-correct with tool-interactive critiquing. *arXiv preprint arXiv:2305.11738* (2023).

48. Chen, D. *et al.* MLLM-as-a-judge: Assessing multimodal LLM-as-a-judge with vision-language benchmark. in *Forty-first International Conference on Machine Learning* (2024).

49. Szymanski, A. *et al.* Limitations of the LLM-as-a-judge approach for evaluating LLM outputs in expert knowledge tasks. in *Proceedings of the 30th International Conference on Intelligent User Interfaces* 952–966 (2025).

50. Shayegani, E. *et al.* Survey of vulnerabilities in large language models revealed by adversarial attacks. *arXiv preprint arXiv:2310.10844* (2023).

51. He, J. & Vechev, M. Large language models for code: Security hardening and adversarial testing. in *Proceedings of the 2023 ACM SIGSAC Conference on Computer and Communications Security* 1865–1879 (2023).

52. Zhu, K. *et al.* PromptBench: Towards evaluating the robustness of large language models on adversarial prompts. *Proceedings of the 1st ACM workshop on large AI systems and models with privacy and safety analysis* (2023).

53. Fu, X. *et al.* Misusing tools in large language models with visual adversarial examples. *arXiv preprint arXiv:2310.03185* (2023).

54. Zhang, Z. *et al.* Defending large language models against jailbreaking attacks through goal prioritization. in *Proceedings of the 62nd Annual Meeting of the Association for*

Computational Linguistics (Volume 1: Long Papers) 8865–8887 (2024).

55. Chao, P. *et al.* JailbreakBench: An open robustness benchmark for jailbreaking large language models. *Advances in Neural Information Processing Systems* **37**, 55005–55029 (2024).
56. Zhao, Y. *et al.* On evaluating adversarial robustness of large vision-language models. *Advances in Neural Information Processing Systems* **36**, 54111–54138 (2023).
57. Ma, X. *et al.* Safety at scale: A comprehensive survey of large model safety. *arXiv preprint arXiv:2502.05206* (2025).
58. Zou, A. *et al.* Universal and transferable adversarial attacks on aligned language models. *arXiv preprint arXiv:2307.15043* (2023).
59. Lapid, R., Langberg, R. & Sipper, M. Open sesame! universal black-box jailbreaking of large language models. *Applied Sciences* **14**, 7150 (2024).
60. Wang, J. *et al.* Adversarial demonstration attacks on large language models. *arXiv preprint arXiv:2305.14950* (2023).
61. Zhou, X., Qiang, Y., Zade, S. Z., Khanduri, P. & Zhu, D. Hijacking large language models via adversarial in-context learning. *arXiv preprint arXiv:2311.09948* (2023).
62. Qi, X. *et al.* Visual adversarial examples jailbreak aligned large language models. in *Proceedings of the AAAI conference on artificial intelligence* vol. 38 21527–21536 (2024).
63. Chao, P. *et al.* Jailbreaking black box large language models in twenty queries. in *2025 IEEE Conference on Secure and Trustworthy Machine Learning (SaTML)* 23–42 (2025).
64. Wang, Z. *et al.* Foot in the door: Understanding large language model jailbreaking via cognitive psychology. *arXiv preprint arXiv:2402.15690* (2024).
65. Zhang, J. *et al.* Adversarial prompt tuning for vision-language models. in *European*

conference on computer vision 56–72 (Springer, 2024).

66. Shneiderman, B. Bridging the gap between ethics and practice: guidelines for reliable, safe, and trustworthy human-centered AI systems. *ACM Transactions on Interactive Intelligent Systems (TiiS)* **10**, 1–31 (2020).
67. Hubinger, E. Anthropic: Responsible Scaling Policy. *SuperIntelligence-Robotics-Safety & Alignment* **2**, (2025).
68. Wang, Y. *et al.* Large Language Model is Secretly a Protein Sequence Optimizer. *arXiv preprint arXiv:2501.09274* (2025).
69. Jumper, J. *et al.* Highly accurate protein structure prediction with AlphaFold. *Nature* **596**, 583–589 (2021).
70. Takahashi, K. *et al.* Induction of pluripotent stem cells from adult human fibroblasts by defined factors. *Cell* **131**, 861–872 (2007).
71. Lin, Z. *et al.* Language models of protein sequences at the scale of evolution enable accurate structure prediction. *BioRxiv* **2022**, 500902 (2022).
72. Baek, M. *et al.* Accurate prediction of protein structures and interactions using a three-track neural network. *Science* **373**, 871–876 (2021).
73. Bommasani, R. *et al.* On the opportunities and risks of foundation models. *arXiv preprint arXiv:2108.07258*.
74. Erhan, D., Courville, A., Bengio, Y. & Vincent, P. Why does unsupervised pre-training help deep learning? in *Proceedings of the thirteenth international conference on artificial intelligence and statistics* 201–208 (JMLR Workshop and Conference Proceedings, 2010).
75. Radford, A., Narasimhan, K., Salimans, T., Sutskever, I. & Others. Improving language understanding by generative pre-training. *Available online:*

https://cdn.openai.com/research-covers/language-unsupervised/language_understanding_paper.pdf (2018). (accessed on 17 February 2025)

76. Team, G. *et al.* Gemini: a family of highly capable multimodal models. *arXiv preprint arXiv:2312. 11805* (2023).
77. Achiam, J. *et al.* GPT-4 technical report. *arXiv preprint arXiv:2303.08774* (2023).
78. Chowdhery, A. *et al.* PaLM: Scaling language modeling with pathways. *arXiv preprint arXiv:2204.02311* (2022).
79. Anil, R. *et al.* PaLM 2 technical report. *arXiv preprint arXiv:2305.10403* (2023).
80. Wei, J. *et al.* Chain-of-thought prompting elicits reasoning in large language models. *Advances in neural information processing systems* **35**, 24824–24837 (2022).
81. Kahneman, D. *Thinking, Fast and Slow*. (Farrar, Straus and Giroux, 2011).
82. Silver, D. *et al.* Mastering the game of Go with deep neural networks and tree search. *Nature* **529**, 484–489 (2016).
83. Brown, N. & Sandholm, T. Superhuman AI for multiplayer poker. *Science* **365**, 885–890 (2019).
84. Yao, S. *et al.* Tree of thoughts: Deliberate problem solving with large language models. *Advances in neural information processing systems* **36**, 11809–11822 (2023).
85. Zelikman, E., Wu, Y., Mu, J. & Goodman, N. Star: Bootstrapping reasoning with reasoning. *Advances in Neural Information Processing Systems* **35**, 15476–15488 (2022).
86. Chen, L. *et al.* Are more LLM calls all you need? towards the scaling properties of compound AI systems. *Advances in Neural Information Processing Systems* **37**, 45767–45790 (2024).
87. Snell, C., Lee, J., Xu, K. & Kumar, A. Scaling LLM test-time compute optimally can be

more effective than scaling model parameters. *arXiv preprint arXiv:2408.03314* (2024).

88. Brown, B, et al. Large language monkeys: Scaling inference compute with repeated sampling. *arXiv preprint arXiv:2407.21787* (2024).
89. Muennighoff, N. *et al.* s1: Simple test-time scaling. *arXiv preprint arXiv:2501.19393* (2025).
90. Tu, T. *et al.* Towards conversational diagnostic artificial intelligence. *Nature* **642**, 442–450 (2025).
91. Guo, D. *et al.* Deepseek-R1: Incentivizing reasoning capability in LLM via reinforcement learning. *arXiv preprint arXiv:2501.12948* (2025).
92. Jumper, J. *et al.* Highly accurate protein structure prediction with AlphaFold. *Nature* **596**, 583–589 (2021).
93. Wong, F. *et al.* Discovery of a structural class of antibiotics with explainable deep learning. *Nature* **626**, 177–185 (2024).
94. Zambaldi, V. *et al.* De novo design of high-affinity protein binders with AlphaProteo. *arXiv preprint arXiv:2409.08022* (2024).
95. Merchant, A. *et al.* Scaling deep learning for materials discovery. *Nature* **624**, 80–85 (2023).
96. Lu, C. *et al.* The AI scientist: Towards fully automated open-ended scientific discovery. *arXiv preprint arXiv:2408.06292* (2024).
97. Schmidgall, S. *et al.* Agent laboratory: Using LLM agents as research assistants. *arXiv preprint arXiv:2501.04227* (2025).
98. Liang, W. *et al.* Can large language models provide useful feedback on research papers? A large-scale empirical analysis. *NEJM AI* **1**, AIoa2400196 (2024).

99. Skarlinski, M. D. *et al.* Language agents achieve superhuman synthesis of scientific knowledge. *arXiv preprint arXiv:2409.13740* (2024).

100. Zhou, Y. *et al*. Hypothesis generation with large language models. *Proceedings of the 1st Workshop on NLP for Science (NLP4Science)* 117–139 (2024).

101. Ifargan, T., Hafner, L., Kern, M., Alcalay, O. & Kishony, R. Autonomous LLM-driven research—from data to human-verifiable research papers. *NEJM AI* **2**, AIoa2400555 (2025).

102. Swanson, K., Wu, W., Bulaong, N. L., Pak, J. E. & Zou, J. The Virtual Lab: AI Agents Design New SARS-CoV-2 Nanobodies with Experimental Validation. *bioRxiv 2024.11.11.623004* (2024).

103. Boiko, D. A., MacKnight, R., Kline, B. & Gomes, G. Autonomous chemical research with large language models. *Nature* **624**, 570–578 (2023).

104. Singhal, K. *et al.* Large language models encode clinical knowledge. *Nature* **620**, 172–180 (2023).

105. Saab, K. *et al.* Capabilities of Gemini models in medicine. *arXiv preprint arXiv:2404.18416* (2024).

106. Taylor, R. *et al.* Galactica: A large language model for science. *arXiv preprint arXiv:2211.09085* (2022).

107. Chaves, J. M. Z. *et al.* Tx-LLM: A large language model for therapeutics. *arXiv preprint arXiv:2406.06316* (2024).

108. Tu, T. *et al.* Genetic discovery enabled by a large language model. *bioRxiv 2023.11.09.566468* (2023).

109. Nguyen, E. *et al.* Sequence modeling and design from molecular to genome scale with Evo. *Science* **386**, eado9336 (2024).

110. Lin, Z. *et al.* Evolutionary-scale prediction of atomic-level protein structure with a language model. *Science* **379**, 1123–1130 (2023).

111. Ruffolo, J. A. *et al.* Design of highly functional genome editors by modeling the universe of CRISPR-Cas sequences. *bioRxiv 2024.04.22.590591* (2024).

112. Shaw, P. *et al.* ProtEx: a retrieval-augmented approach for protein function prediction. *bioRxiv 2024.05.30.596539* (2024).

113. Pushpakom, S. *et al.* Drug repurposing: progress, challenges and recommendations. *Nat Rev Drug Discov* **18**, 41–58 (2019).

114. Krishnamurthy, N., Grimshaw, A. A., Axson, S. A., Choe, S. H. & Miller, J. E. Drug repurposing: a systematic review on root causes, barriers and facilitators. *BMC Health Serv Res* **22**, 970 (2022).

115. Zitnik, M., Agrawal, M. & Leskovec, J. Modeling polypharmacy side effects with graph convolutional networks. *Bioinformatics* **34**, i457–i466 (2018).

116. Morselli Gysi, D. *et al.* Network medicine framework for identifying drug-repurposing opportunities for COVID-19. *Proc Natl Acad Sci USA* **118**, (2021).

117. Huang, K. *et al.* A foundation model for clinician-centered drug repurposing. *Nat Med* **30**, 3601–3613 (2024).
