# Supplementary material for: Accelerating scientific discovery with Co-Scientist
Source: Nature. 2026 May 19;655(8122):487–96. doi: 10.1038/s41586-026-10644-y (PMC13345910; doi:10.1038/s41586-026-10644-y)
Supplement: Supplementary file 2 — Reporting Summary [file 41586_2026_10644_MOESM2_ESM.pdf]

nature portfolio

Corresponding author(s): Juraj Gottweis, Wei-Hung Weng, Pushmeet Kohli, Annalisa Pawlosky, Alan Karthikesalingam, Vivek Natarajan

Last updated by author(s): May 8, 2026

Reporting Summary

Nature Portfolio wishes to improve the reproducibility of the work that we publish. This form provides structure for consistency and transparency in reporting. For further information on Nature Portfolio policies, see our Editorial Policies and the Editorial Policy Checklist.

Statistics

For all statistical analyses, confirm that the following items are present in the figure legend, table legend, main text, or Methods section.

n/a	Confirmed	
☐	☒	The exact sample size (*n*) for each experimental group/condition, given as a discrete number and unit of measurement
☐	☒	A statement on whether measurements were taken from distinct samples or whether the same sample was measured repeatedly
☐	☒	The statistical test(s) used AND whether they are one- or two-sided<br>*Only common tests should be described solely by name; describe more complex techniques in the Methods section.*
☒	☐	A description of all covariates tested
☒	☐	A description of any assumptions or corrections, such as tests of normality and adjustment for multiple comparisons
☐	☒	A full description of the statistical parameters including central tendency (e.g. means) or other basic estimates (e.g. regression coefficient) AND variation (e.g. standard deviation) or associated estimates of uncertainty (e.g. confidence intervals)
☐	☒	For null hypothesis testing, the test statistic (e.g. *F*, *t*, *r*) with confidence intervals, effect sizes, degrees of freedom and *P* value noted<br>*Give P values as exact values whenever suitable.*
☒	☐	For Bayesian analysis, information on the choice of priors and Markov chain Monte Carlo settings
☒	☐	For hierarchical and complex designs, identification of the appropriate level for tests and full reporting of outcomes
☒	☐	Estimates of effect sizes (e.g. Cohen's *d*, Pearson's *r*), indicating how they were calculated

Our web collection on statistics for biologists contains articles on many of the points above.

Software and code

Policy information about availability of computer code

Data collection	The system, algorithms and scripts were implemented using Python 3.11.7. Scientific literature and data were collected via web search APIs and internal data pipelines compatible with the Gemini 2.0 model infrastructure. Data collection for in vitro cell viability was performed using a multimode microplate reader.
Data analysis	The full source code for the Co-Scientist system is not publicly available. The foundational LLM model, Gemini, used in Co-Scientist is publicly available via APIs, Google AI studio, the Gemini App and other surfaces. The analysis codes were developed using Python (version 3.11.7). Data handling and visualization were performed using pandas (version 2.1.4), numpy (version 1.26.4), seaborn (version 0.12.2), and matplotlib (version 3.8.0). For in vitro validations, dose-response curve fitting and IC50 estimation were performed using GraphPad Prism (version 10.6.0, GraphPad Software). Synergy analysis and Combination Index calculations were performed using Julius AI statistical software (accessed November 2025).

For manuscripts utilizing custom algorithms or software that are central to the research but not yet described in published literature, software must be made available to editors and reviewers. We strongly encourage code deposition in a community repository (e.g. GitHub). See the Nature Portfolio guidelines for submitting code & software for further information.

Data

Policy information about availability of data

All manuscripts must include a data availability statement. This statement should provide the following information, where applicable:

- Accession codes, unique identifiers, or web links for publicly available datasets
- A description of any restrictions on data availability
- For clinical datasets or third party data, please ensure that the statement adheres to our policy

Except for the three real-world validation tasks (drug repurposing for AML, novel target discovery for liver fibrosis, mechanism explanation of gene transfer evolution), the remaining datasets used for development, benchmarking and evaluation of the AI systems are open source or otherwise accessible publicly with permissions. Specifically, the GPQA diamond dataset is publicly available at Hugging Face (https://huggingface.co/datasets/Idavidrein/gpqa). The Cancer Dependency Map (DepMap) Q2 2024 data used for computational sanity checks is publicly available at the DepMap portal (https://depmap.org/portal/). The curated drug targets dataset from the Open Targets Platform is available at https://platform.opentargets.org/downloads.

Research involving human participants, their data, or biological material

Policy information about studies with human participants or human data. See also policy information about sex, gender (identity/presentation), and sexual orientation and race, ethnicity and racism.

Reporting on sex and gender	N/A
Reporting on race, ethnicity, or other socially relevant groupings	N/A
Population characteristics	N/A
Recruitment	N/A
Ethics oversight	N/A

Note that full information on the approval of the study protocol must also be provided in the manuscript.

Field-specific reporting

Please select the one below that is the best fit for your research. If you are not sure, read the appropriate sections before making your selection.

☒ Life sciences ☐ Behavioural & social sciences ☐ Ecological, evolutionary & environmental sciences

For a reference copy of the document with all sections, see nature.com/documents/nr-reporting-summary-flat.pdf

Life sciences study design

All studies must disclose on these points even when the disclosure is negative.

Sample size	No statistical methods were used to predetermine sample sizes. For the computational evaluations, the sample size of 203 diverse research goals, 15 expert-curated goals, and 11 goals for LLM-as-a-judge preference were chosen to ensure a broad representation of scientific domains, robust statistical averaging across varying complexity, and to mitigate evaluator bias. For the in vitro validations, five distinct AML cell lines (MOLM-13, KG-1a, HL-60, NOMO-1, and TK6) were tested to model the molecular and genetic heterogeneity of the disease. Experiments were performed in biological triplicates (n=3). This sample size was not predetermined by statistical methods but was chosen based on widely accepted standard practices for preliminary in vitro dose-response viability screening. Given the large effect sizes typical of such preliminary pharmacological assays, three independent biological replicates provide the necessary degrees of freedom to calculate standard deviation, assess assay consistency, and robustly fit non-linear regression curves for IC50 estimation.
Data exclusions	No data were excluded from the computational or in vitro laboratory analyses.
Replication	All in vitro dose-response (single-agent screening) and cell viability assays (fixed-ratio combination treatments) were performed in independent biological triplicates (n=3). For computational hypothesis generation and LLM-as-a-judge evaluations, the system generated responses or evaluations independently across multiple discrete runs. All attempts at replication (both in vitro and computational) were successful. The resulting in vitro data were presented as the mean ± standard deviation of these triplicates.
Randomization	Randomization was not relevant for the in vitro experiments, as these involved standardized dose-response testing where specified drug concentrations were deterministically applied to cell cultures. For the human evaluation of the Co-Scientist and other LLMs, outputs were assessed by experts, the model outputs were presented randomly.
Blinding	The human expert evaluation of the LLM-generated outputs was explicitly blinded, ensuring that independent domain experts were completely unaware of which model generated the hypotheses they were scoring. Blinding was not applicable to the in vitro cell viability screening assays because these experiments involve standardized, automated readouts (absorbance at 490 nm using a multimode microplate

reader) that are objective and not subject to investigator interpretation bias. Furthermore, knowledge of the drug identity is required by the experimenter to prepare the appropriate molar concentration ranges and dilution series for accurate IC50 determination.

Reporting for specific materials, systems and methods

We require information from authors about some types of materials, experimental systems and methods used in many studies. Here, indicate whether each material, system or method listed is relevant to your study. If you are not sure if a list item applies to your research, read the appropriate section before selecting a response.

Materials & experimental systems

n/a	Involved in the study	
☒	☐	Antibodies
☒	☐	Eukaryotic cell lines
☒	☐	Palaeontology and archaeology
☒	☐	Animals and other organisms
☒	☐	Clinical data
☐	☒	Dual use research of concern
☒	☐	Plants

Methods

n/a	Involved in the study	
☒	☐	ChIP-seq
☒	☐	Flow cytometry
☒	☐	MRI-based neuroimaging

Dual use research of concern

Policy information about dual use research of concern

Hazards

Could the accidental, deliberate or reckless misuse of agents or technologies generated in the work, or the application of information presented in the manuscript, pose a threat to:

No	Yes	
☒	☐	Public health
☒	☐	National security
☒	☐	Crops and/or livestock
☒	☐	Ecosystems
☒	☐	Any other significant area

Experiments of concern

Does the work involve any of these experiments of concern:

No	Yes	
☒	☐	Demonstrate how to render a vaccine ineffective
☒	☐	Confer resistance to therapeutically useful antibiotics or antiviral agents
☒	☐	Enhance the virulence of a pathogen or render a nonpathogen virulent
☒	☐	Increase transmissibility of a pathogen
☒	☐	Alter the host range of a pathogen
☒	☐	Enable evasion of diagnostic/detection modalities
☒	☐	Enable the weaponization of a biological agent or toxin
☒	☐	Any other potentially harmful combination of experiments and agents

Plants

Seed stocks	*Report on the source of all seed stocks or other plant material used. If applicable, state the seed stock centre and catalogue number. If plant specimens were collected from the field, describe the collection location, date and sampling procedures.*
Novel plant genotypes	*Describe the methods by which all novel plant genotypes were produced. This includes those generated by transgenic approaches, gene editing, chemical/radiation-based mutagenesis and hybridization. For transgenic lines, describe the transformation method, the number of independent lines analyzed and the generation upon which experiments were performed. For gene-edited lines, describe the editor used, the endogenous sequence targeted for editing, the targeting guide RNA sequence (if applicable) and how the editor was applied.*
Authentication	*Describe any authentication procedures for each seed stock used or novel genotype generated. Describe any experiments used to assess the effect of a mutation and, where applicable, how potential secondary effects (e.g. second site T-DNA insertions, mosiacism, off-target gene editing) were examined.*
